# Supplementary figures and images for: Detection of opening motion characteristics in DC circuit breakers based on machine vision (part 2 of 2)
Source: PLoS One. 2025 Feb 3;20(2):e0312253. doi: 10.1371/journal.pone.0312253 (PMC11790151; doi:10.1371/journal.pone.0312253)

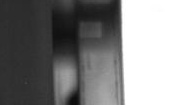

Supplement: S1 File — The relevant data can be obtained from the supporting information. The supporting information is mainly the image data analyzed in this paper. (ZIP) [file pone.0312253.s001.zip › supporting information/repulsion disc/fen20.jpg]

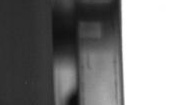

Supplement: S1 File — The relevant data can be obtained from the supporting information. The supporting information is mainly the image data analyzed in this paper. (ZIP) [file pone.0312253.s001.zip › supporting information/repulsion disc/fen21.jpg]

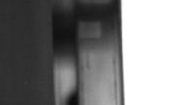

Supplement: S1 File — The relevant data can be obtained from the supporting information. The supporting information is mainly the image data analyzed in this paper. (ZIP) [file pone.0312253.s001.zip › supporting information/repulsion disc/fen22.jpg]

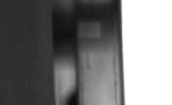

Supplement: S1 File — The relevant data can be obtained from the supporting information. The supporting information is mainly the image data analyzed in this paper. (ZIP) [file pone.0312253.s001.zip › supporting information/repulsion disc/fen23.jpg]

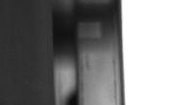

Supplement: S1 File — The relevant data can be obtained from the supporting information. The supporting information is mainly the image data analyzed in this paper. (ZIP) [file pone.0312253.s001.zip › supporting information/repulsion disc/fen24.jpg]

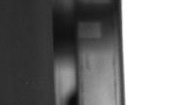

Supplement: S1 File — The relevant data can be obtained from the supporting information. The supporting information is mainly the image data analyzed in this paper. (ZIP) [file pone.0312253.s001.zip › supporting information/repulsion disc/fen25.jpg]

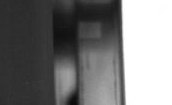

Supplement: S1 File — The relevant data can be obtained from the supporting information. The supporting information is mainly the image data analyzed in this paper. (ZIP) [file pone.0312253.s001.zip › supporting information/repulsion disc/fen26.jpg]

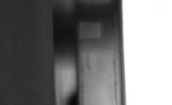

Supplement: S1 File — The relevant data can be obtained from the supporting information. The supporting information is mainly the image data analyzed in this paper. (ZIP) [file pone.0312253.s001.zip › supporting information/repulsion disc/fen27.jpg]

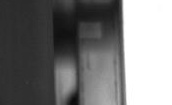

Supplement: S1 File — The relevant data can be obtained from the supporting information. The supporting information is mainly the image data analyzed in this paper. (ZIP) [file pone.0312253.s001.zip › supporting information/repulsion disc/fen28.jpg]

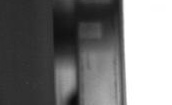

Supplement: S1 File — The relevant data can be obtained from the supporting information. The supporting information is mainly the image data analyzed in this paper. (ZIP) [file pone.0312253.s001.zip › supporting information/repulsion disc/fen29.jpg]

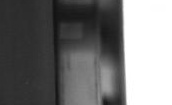

Supplement: S1 File — The relevant data can be obtained from the supporting information. The supporting information is mainly the image data analyzed in this paper. (ZIP) [file pone.0312253.s001.zip › supporting information/repulsion disc/fen3.jpg]

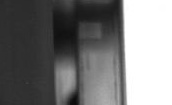

Supplement: S1 File — The relevant data can be obtained from the supporting information. The supporting information is mainly the image data analyzed in this paper. (ZIP) [file pone.0312253.s001.zip › supporting information/repulsion disc/fen30.jpg]

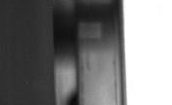

Supplement: S1 File — The relevant data can be obtained from the supporting information. The supporting information is mainly the image data analyzed in this paper. (ZIP) [file pone.0312253.s001.zip › supporting information/repulsion disc/fen31.jpg]

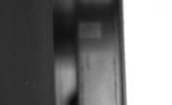

Supplement: S1 File — The relevant data can be obtained from the supporting information. The supporting information is mainly the image data analyzed in this paper. (ZIP) [file pone.0312253.s001.zip › supporting information/repulsion disc/fen32.jpg]

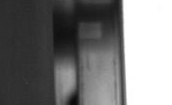

Supplement: S1 File — The relevant data can be obtained from the supporting information. The supporting information is mainly the image data analyzed in this paper. (ZIP) [file pone.0312253.s001.zip › supporting information/repulsion disc/fen33.jpg]

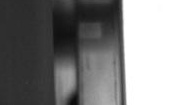

Supplement: S1 File — The relevant data can be obtained from the supporting information. The supporting information is mainly the image data analyzed in this paper. (ZIP) [file pone.0312253.s001.zip › supporting information/repulsion disc/fen34.jpg]

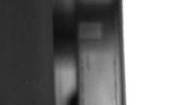

Supplement: S1 File — The relevant data can be obtained from the supporting information. The supporting information is mainly the image data analyzed in this paper. (ZIP) [file pone.0312253.s001.zip › supporting information/repulsion disc/fen35.jpg]

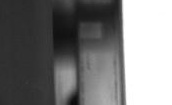

Supplement: S1 File — The relevant data can be obtained from the supporting information. The supporting information is mainly the image data analyzed in this paper. (ZIP) [file pone.0312253.s001.zip › supporting information/repulsion disc/fen36.jpg]

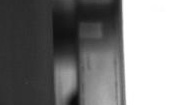

Supplement: S1 File — The relevant data can be obtained from the supporting information. The supporting information is mainly the image data analyzed in this paper. (ZIP) [file pone.0312253.s001.zip › supporting information/repulsion disc/fen37.jpg]

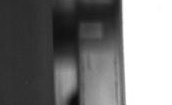

Supplement: S1 File — The relevant data can be obtained from the supporting information. The supporting information is mainly the image data analyzed in this paper. (ZIP) [file pone.0312253.s001.zip › supporting information/repulsion disc/fen38.jpg]

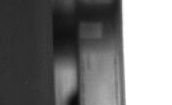

Supplement: S1 File — The relevant data can be obtained from the supporting information. The supporting information is mainly the image data analyzed in this paper. (ZIP) [file pone.0312253.s001.zip › supporting information/repulsion disc/fen39.jpg]

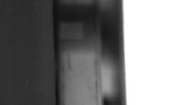

Supplement: S1 File — The relevant data can be obtained from the supporting information. The supporting information is mainly the image data analyzed in this paper. (ZIP) [file pone.0312253.s001.zip › supporting information/repulsion disc/fen4.jpg]

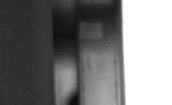

Supplement: S1 File — The relevant data can be obtained from the supporting information. The supporting information is mainly the image data analyzed in this paper. (ZIP) [file pone.0312253.s001.zip › supporting information/repulsion disc/fen40.jpg]

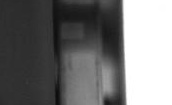

Supplement: S1 File — The relevant data can be obtained from the supporting information. The supporting information is mainly the image data analyzed in this paper. (ZIP) [file pone.0312253.s001.zip › supporting information/repulsion disc/fen5.jpg]

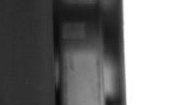

Supplement: S1 File — The relevant data can be obtained from the supporting information. The supporting information is mainly the image data analyzed in this paper. (ZIP) [file pone.0312253.s001.zip › supporting information/repulsion disc/fen6.jpg]

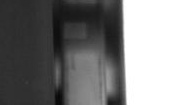

Supplement: S1 File — The relevant data can be obtained from the supporting information. The supporting information is mainly the image data analyzed in this paper. (ZIP) [file pone.0312253.s001.zip › supporting information/repulsion disc/fen7.jpg]

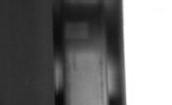

Supplement: S1 File — The relevant data can be obtained from the supporting information. The supporting information is mainly the image data analyzed in this paper. (ZIP) [file pone.0312253.s001.zip › supporting information/repulsion disc/fen8.jpg]

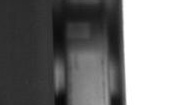

Supplement: S1 File — The relevant data can be obtained from the supporting information. The supporting information is mainly the image data analyzed in this paper. (ZIP) [file pone.0312253.s001.zip › supporting information/repulsion disc/fen9.jpg]
