# Supplementary figures and images for: Detection of opening motion characteristics in DC circuit breakers based on machine vision (part 1 of 2)
Source: PLoS One. 2025 Feb 3;20(2):e0312253. doi: 10.1371/journal.pone.0312253 (PMC11790151; doi:10.1371/journal.pone.0312253)

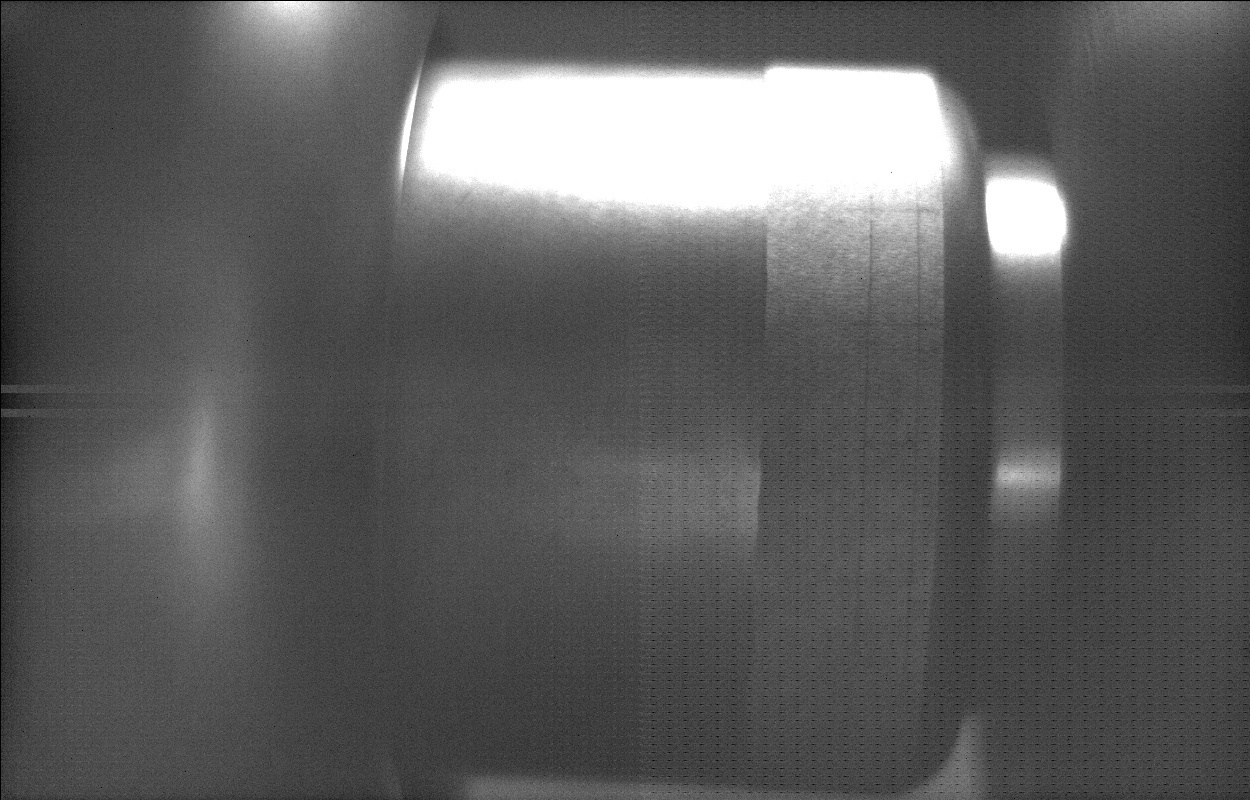

Supplement: S1 File — The relevant data can be obtained from the supporting information. The supporting information is mainly the image data analyzed in this paper. (ZIP) [file pone.0312253.s001.zip › supporting information/Nozzle/OPEN (1).jpg]

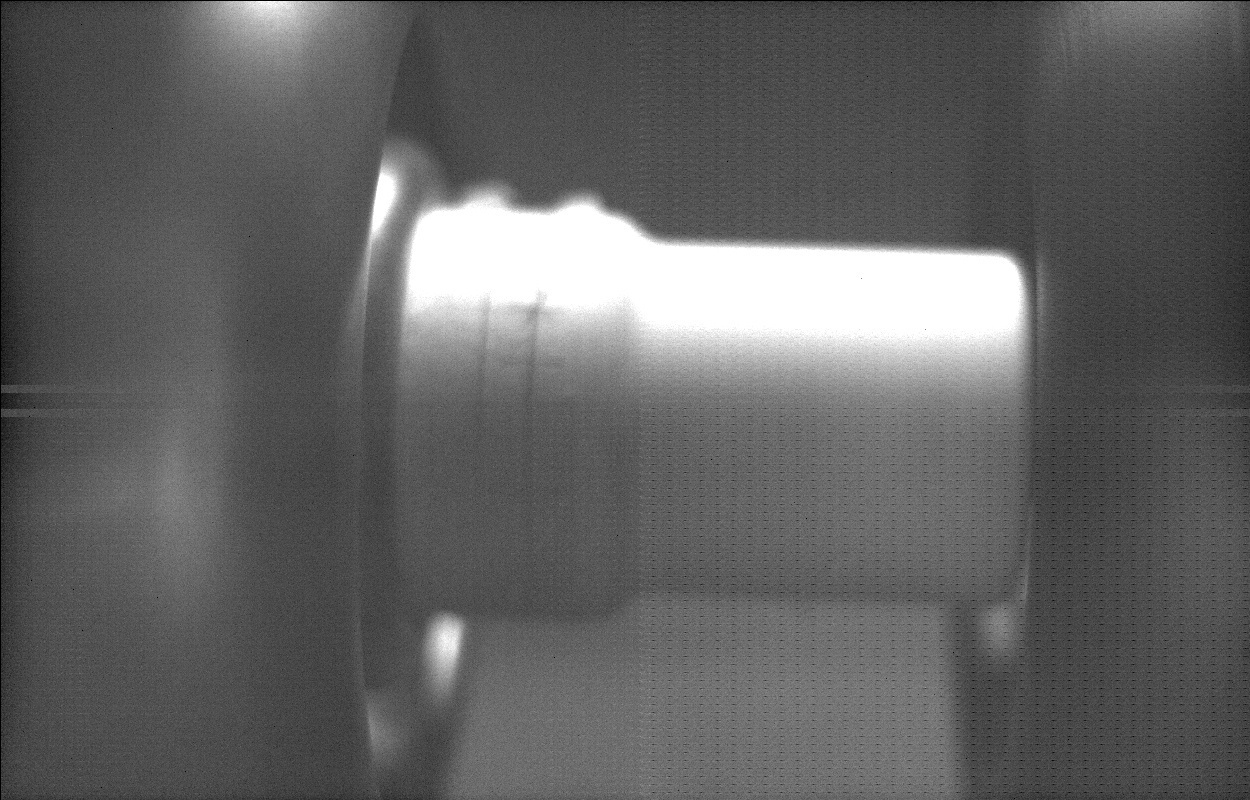

Supplement: S1 File — The relevant data can be obtained from the supporting information. The supporting information is mainly the image data analyzed in this paper. (ZIP) [file pone.0312253.s001.zip › supporting information/Nozzle/OPEN (10).jpg]

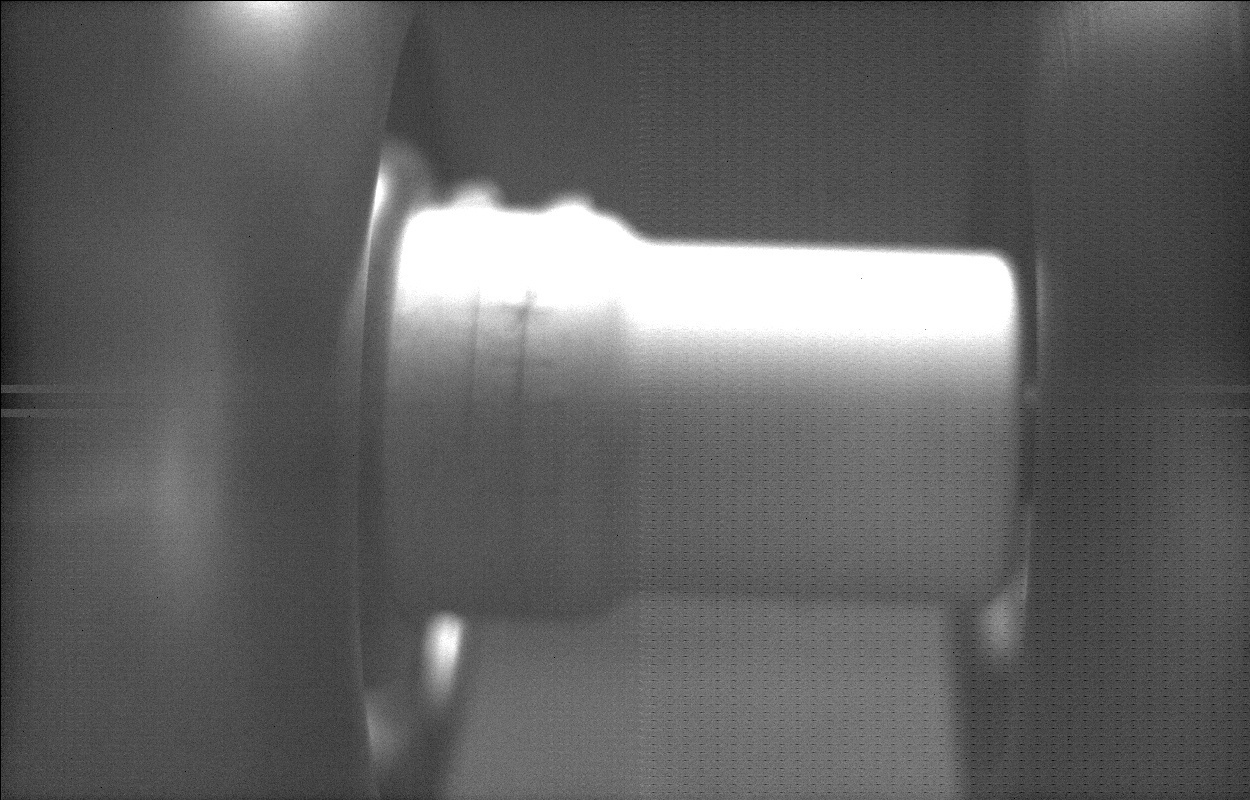

Supplement: S1 File — The relevant data can be obtained from the supporting information. The supporting information is mainly the image data analyzed in this paper. (ZIP) [file pone.0312253.s001.zip › supporting information/Nozzle/OPEN (11).jpg]

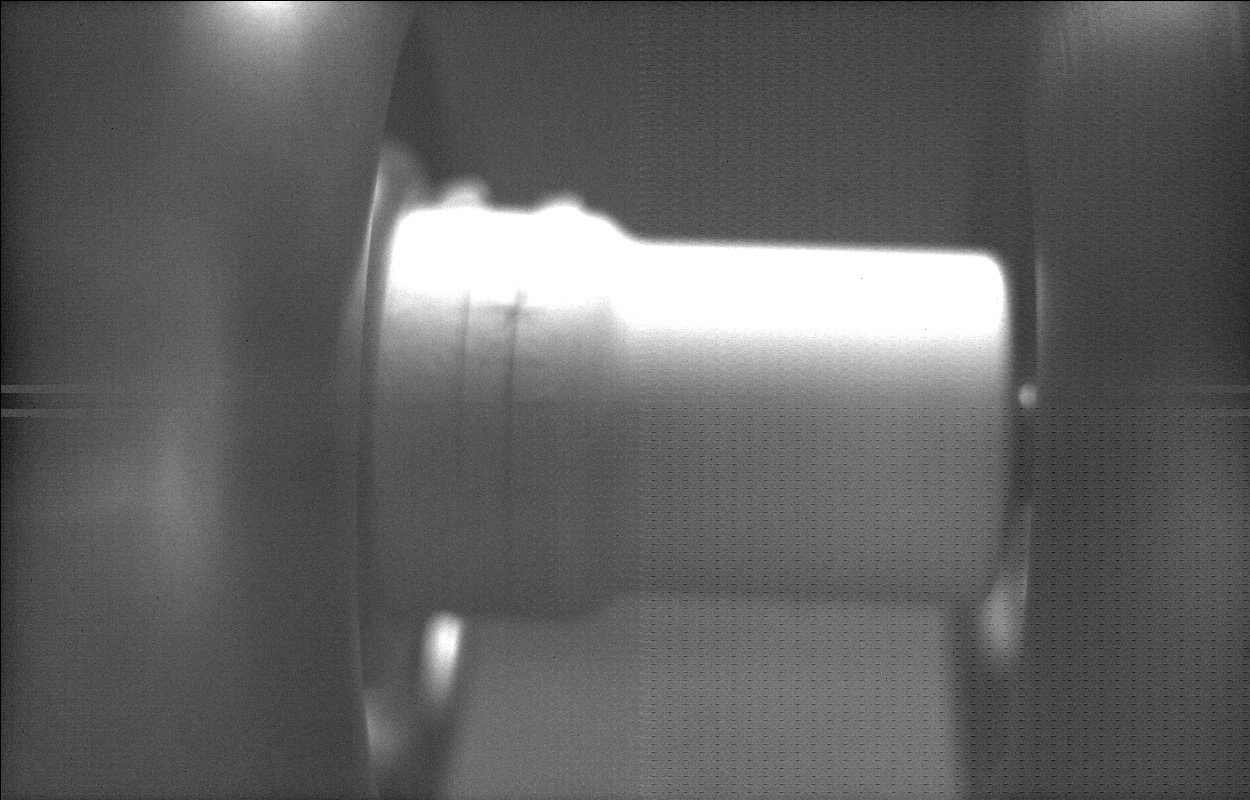

Supplement: S1 File — The relevant data can be obtained from the supporting information. The supporting information is mainly the image data analyzed in this paper. (ZIP) [file pone.0312253.s001.zip › supporting information/Nozzle/OPEN (12).jpg]

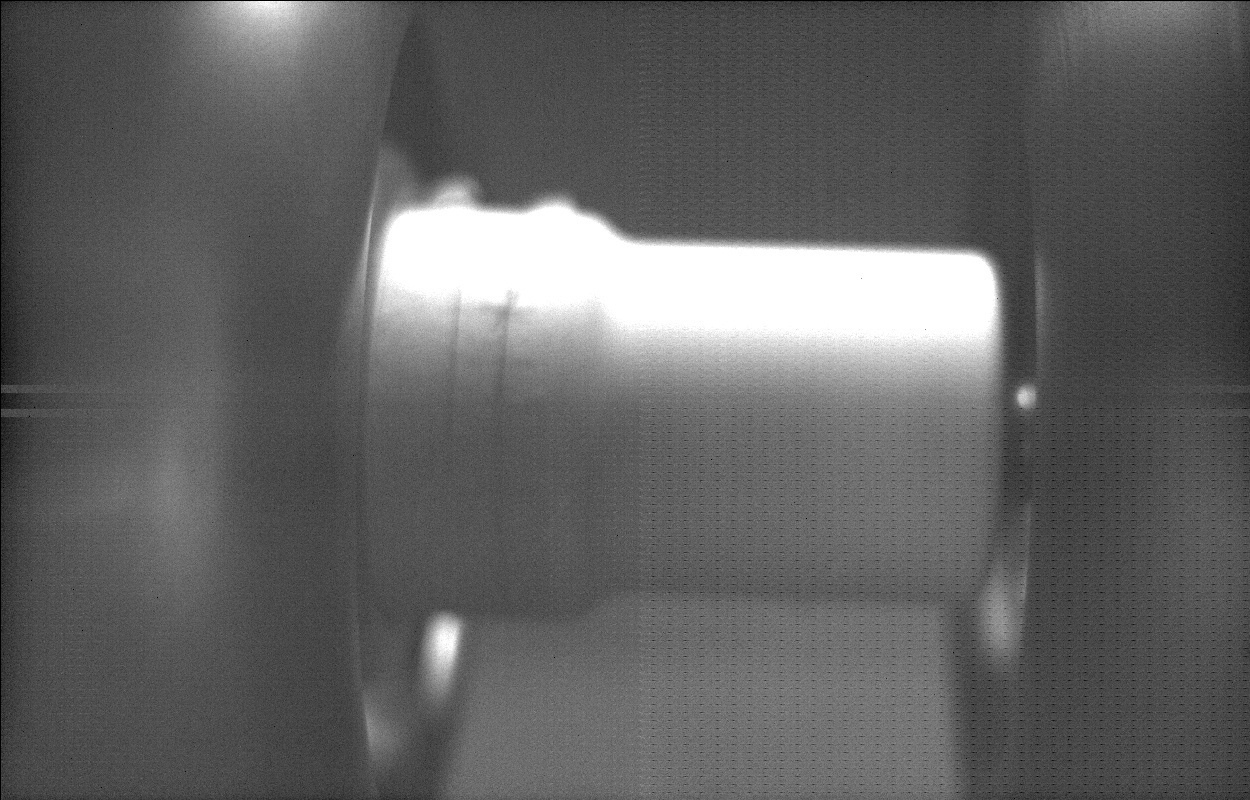

Supplement: S1 File — The relevant data can be obtained from the supporting information. The supporting information is mainly the image data analyzed in this paper. (ZIP) [file pone.0312253.s001.zip › supporting information/Nozzle/OPEN (13).jpg]

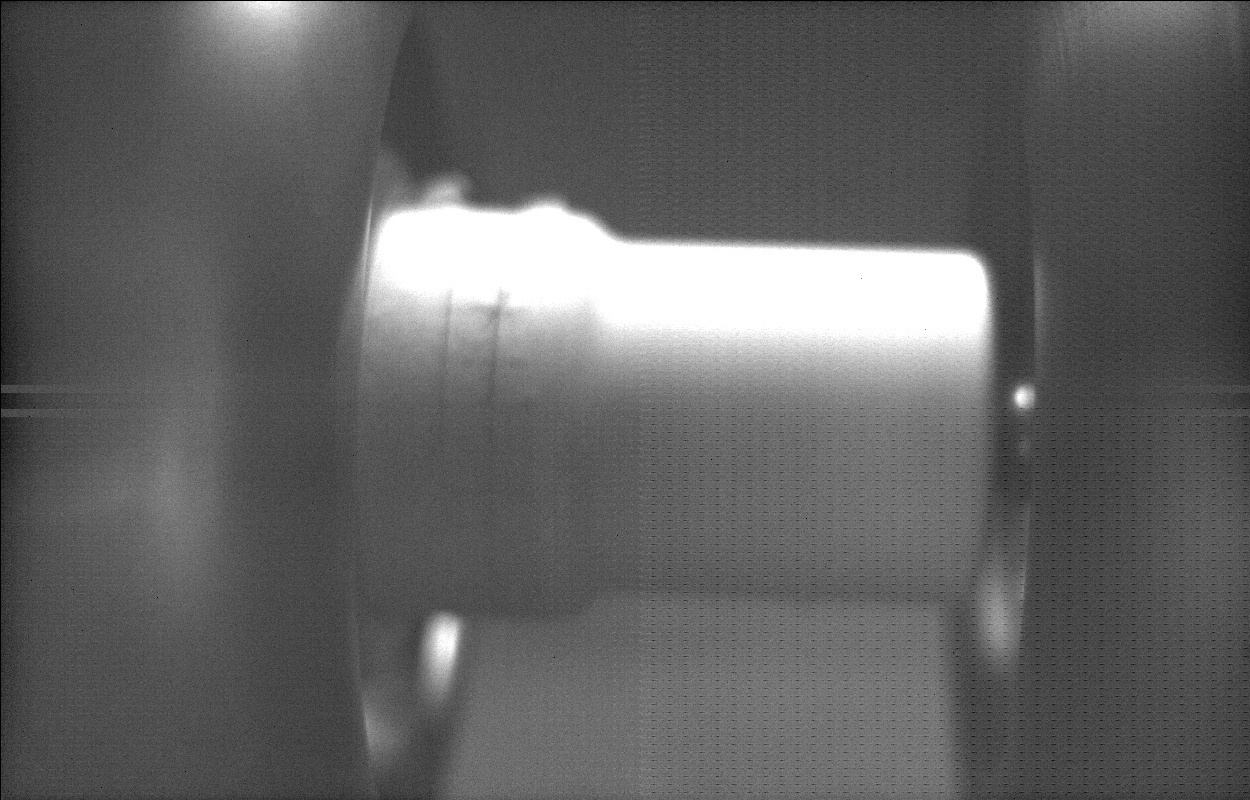

Supplement: S1 File — The relevant data can be obtained from the supporting information. The supporting information is mainly the image data analyzed in this paper. (ZIP) [file pone.0312253.s001.zip › supporting information/Nozzle/OPEN (14).jpg]

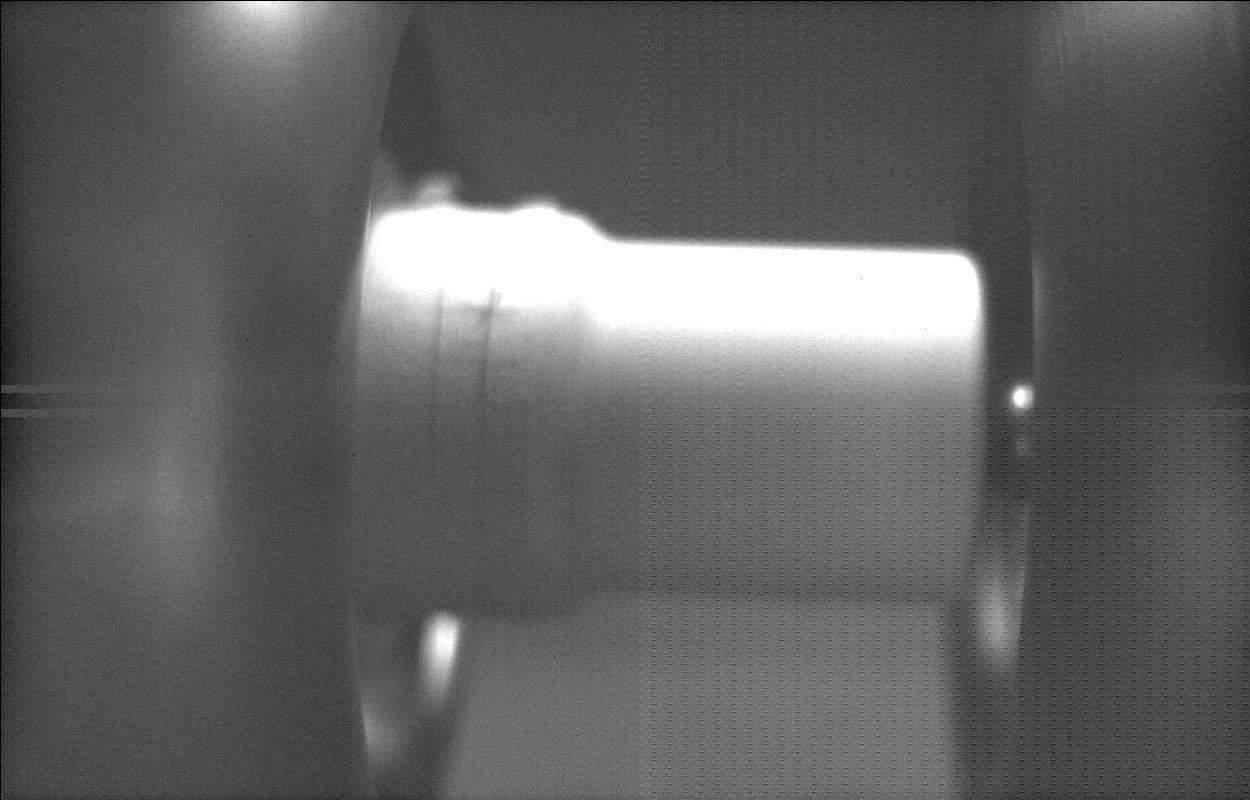

Supplement: S1 File — The relevant data can be obtained from the supporting information. The supporting information is mainly the image data analyzed in this paper. (ZIP) [file pone.0312253.s001.zip › supporting information/Nozzle/OPEN (15).jpg]

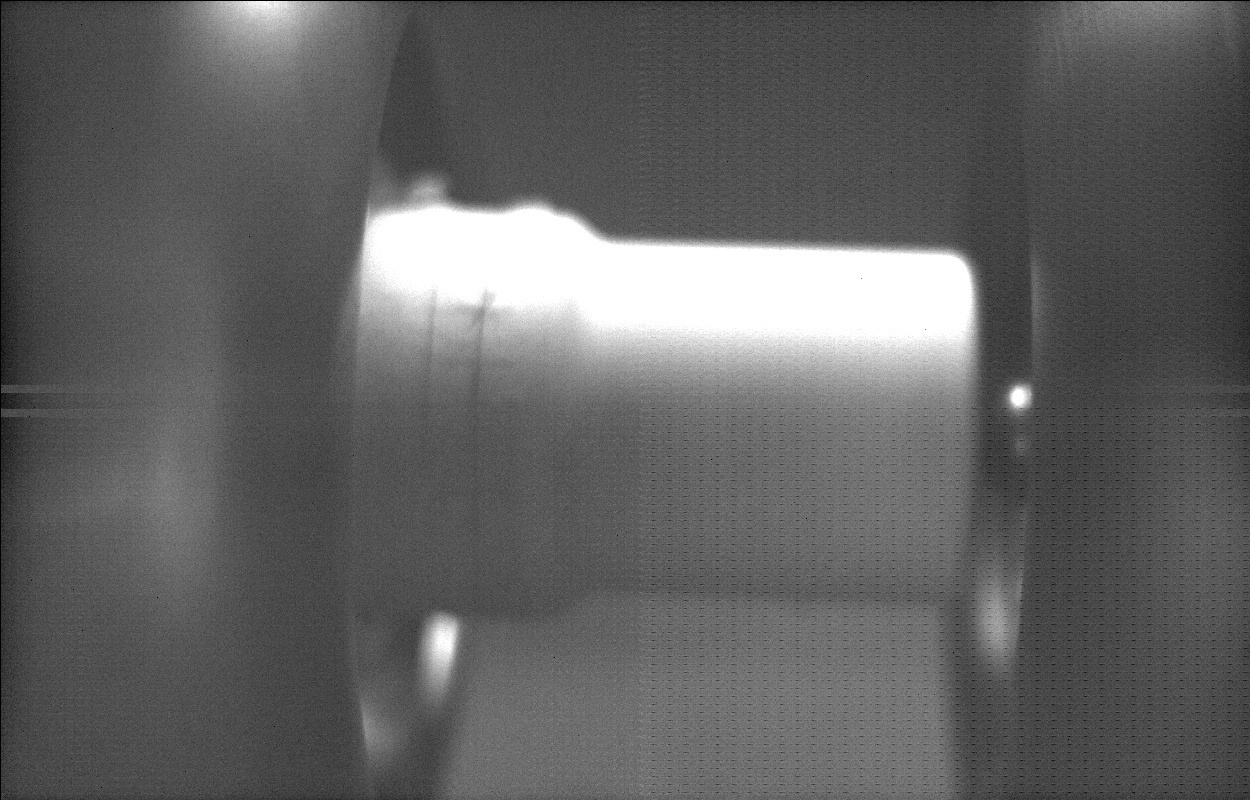

Supplement: S1 File — The relevant data can be obtained from the supporting information. The supporting information is mainly the image data analyzed in this paper. (ZIP) [file pone.0312253.s001.zip › supporting information/Nozzle/OPEN (16).jpg]

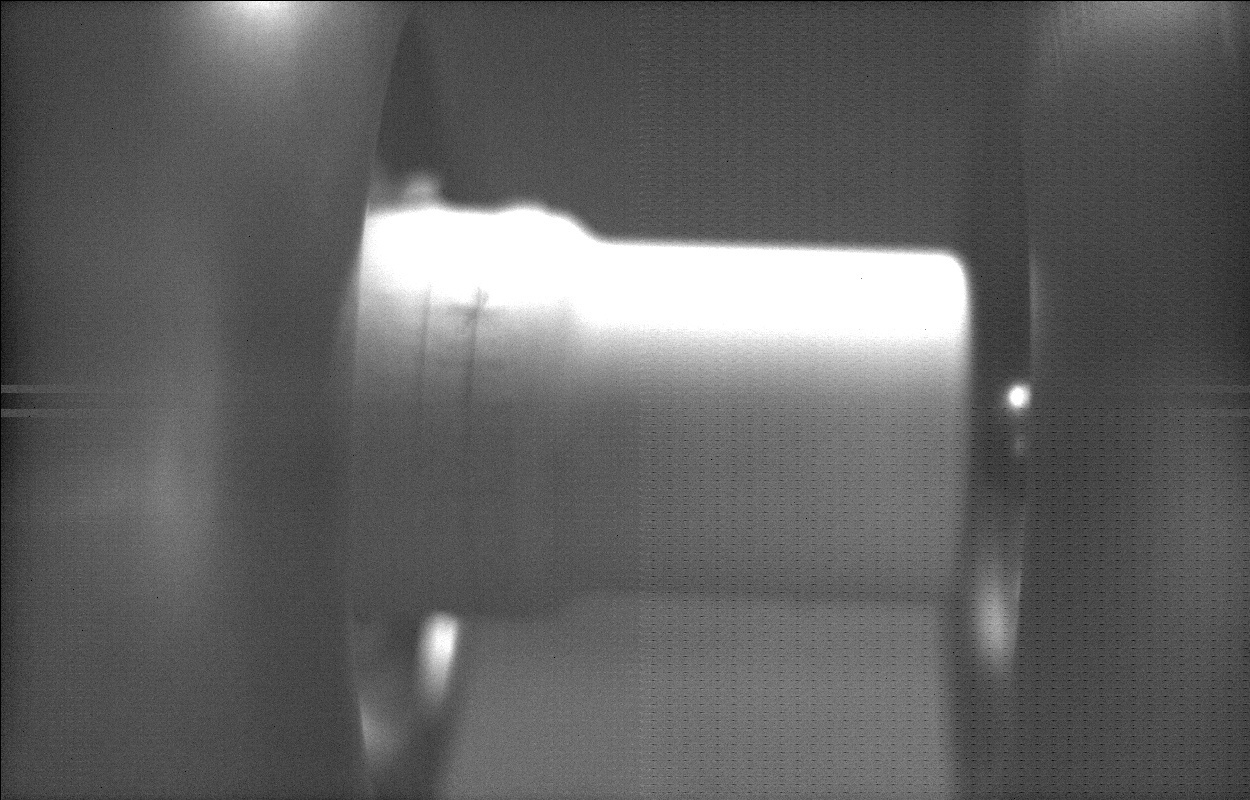

Supplement: S1 File — The relevant data can be obtained from the supporting information. The supporting information is mainly the image data analyzed in this paper. (ZIP) [file pone.0312253.s001.zip › supporting information/Nozzle/OPEN (17).jpg]

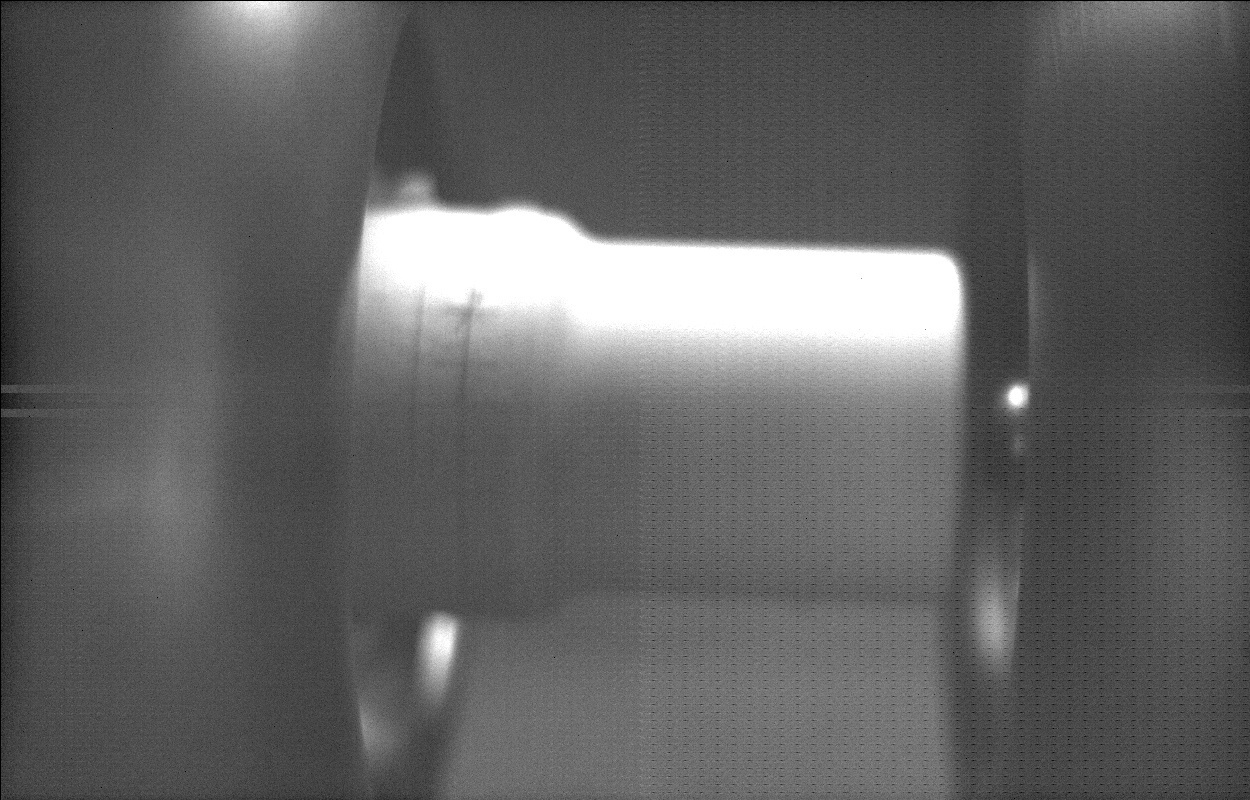

Supplement: S1 File — The relevant data can be obtained from the supporting information. The supporting information is mainly the image data analyzed in this paper. (ZIP) [file pone.0312253.s001.zip › supporting information/Nozzle/OPEN (18).jpg]

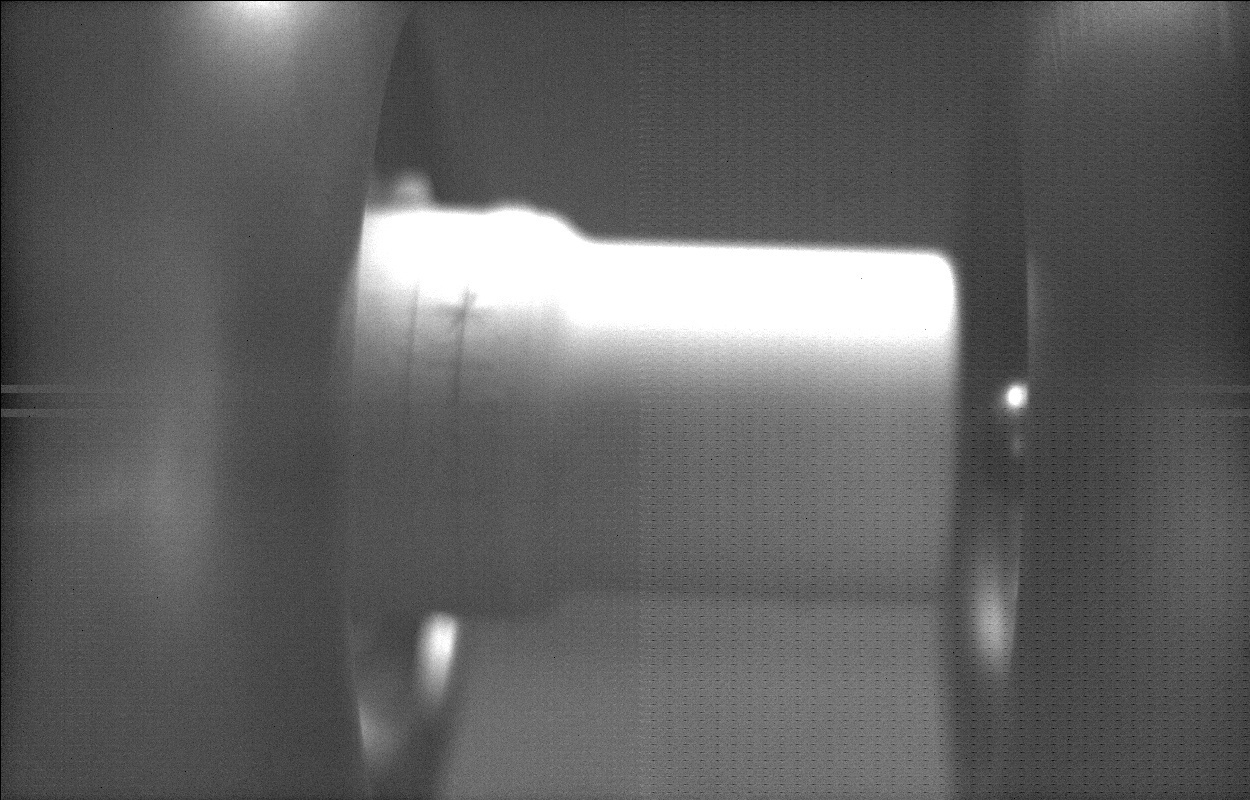

Supplement: S1 File — The relevant data can be obtained from the supporting information. The supporting information is mainly the image data analyzed in this paper. (ZIP) [file pone.0312253.s001.zip › supporting information/Nozzle/OPEN (19).jpg]

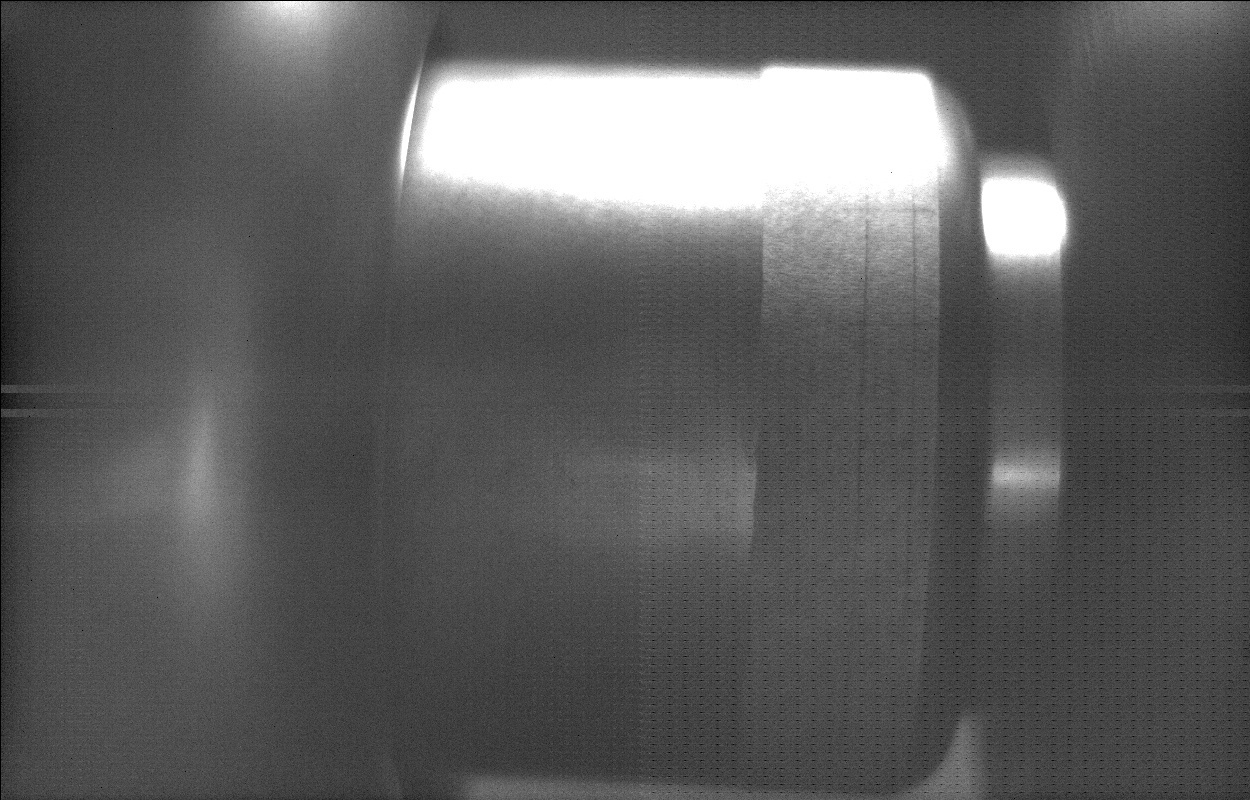

Supplement: S1 File — The relevant data can be obtained from the supporting information. The supporting information is mainly the image data analyzed in this paper. (ZIP) [file pone.0312253.s001.zip › supporting information/Nozzle/OPEN (2).jpg]

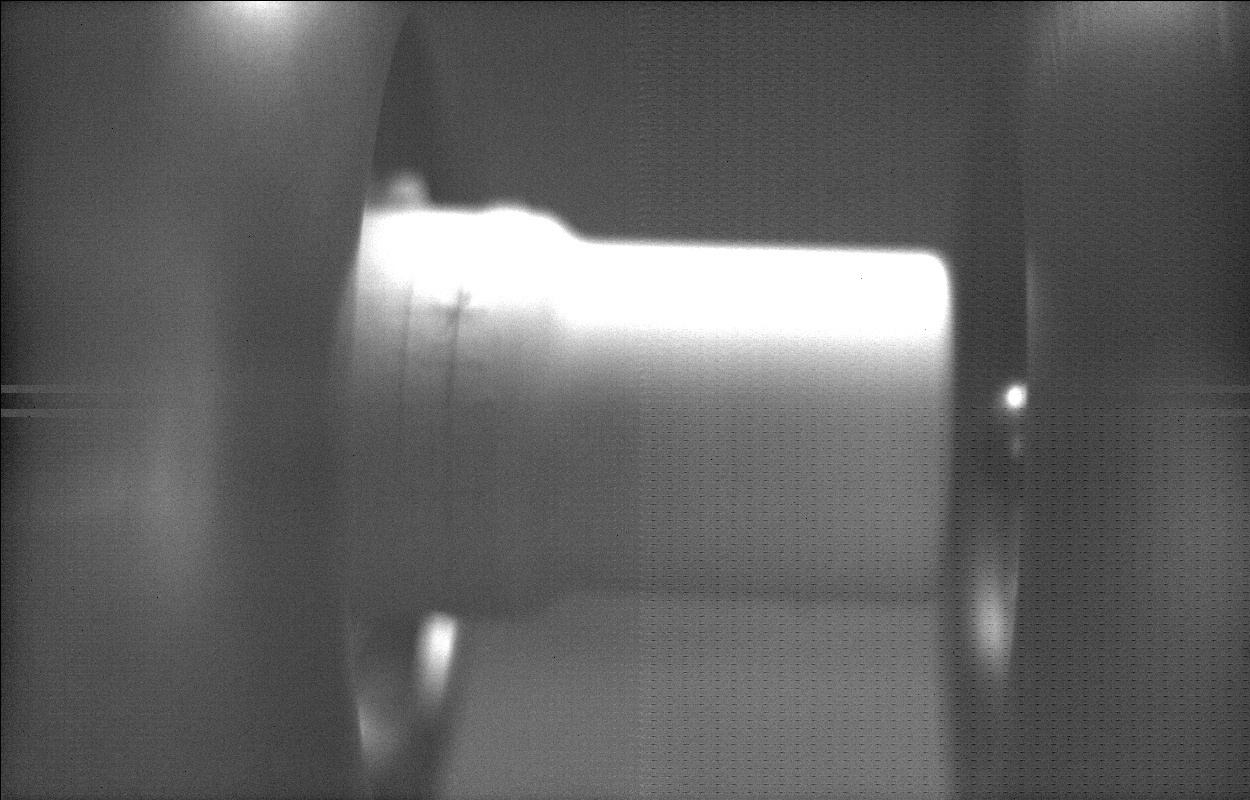

Supplement: S1 File — The relevant data can be obtained from the supporting information. The supporting information is mainly the image data analyzed in this paper. (ZIP) [file pone.0312253.s001.zip › supporting information/Nozzle/OPEN (20).jpg]

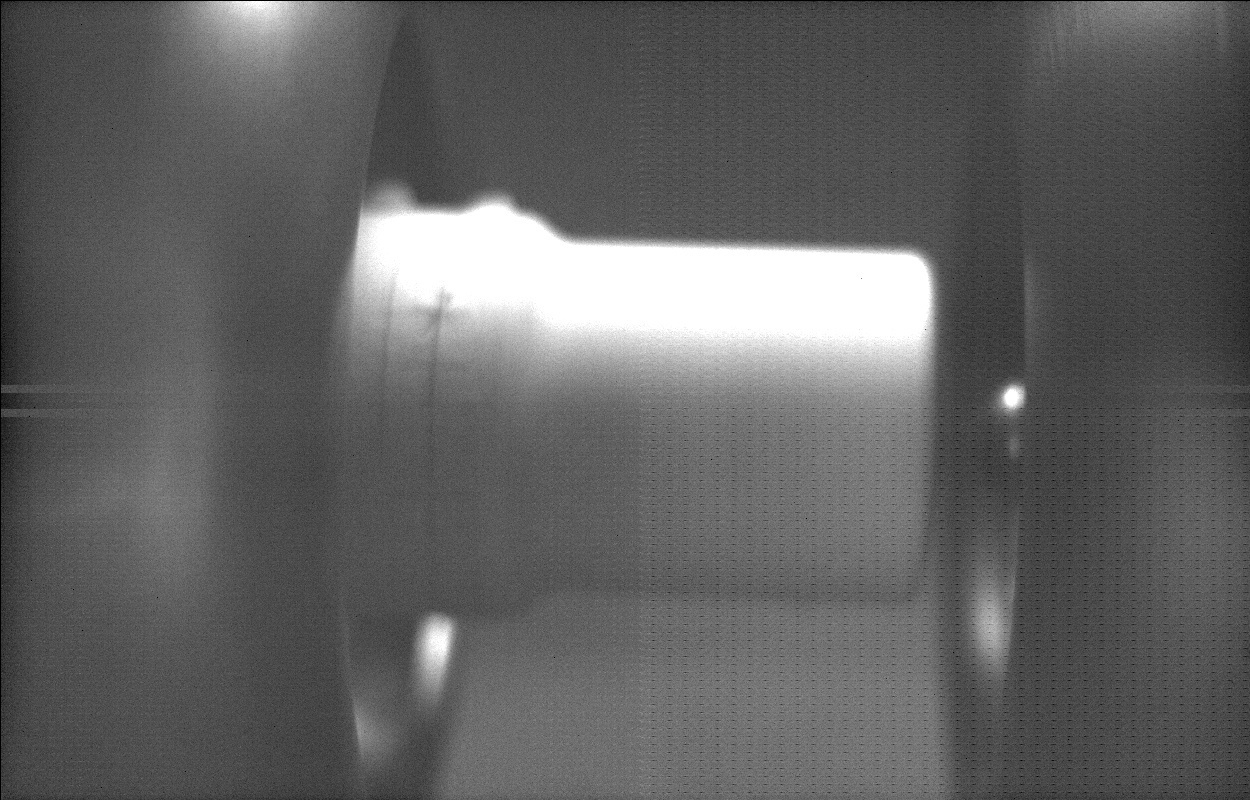

Supplement: S1 File — The relevant data can be obtained from the supporting information. The supporting information is mainly the image data analyzed in this paper. (ZIP) [file pone.0312253.s001.zip › supporting information/Nozzle/OPEN (21).jpg]

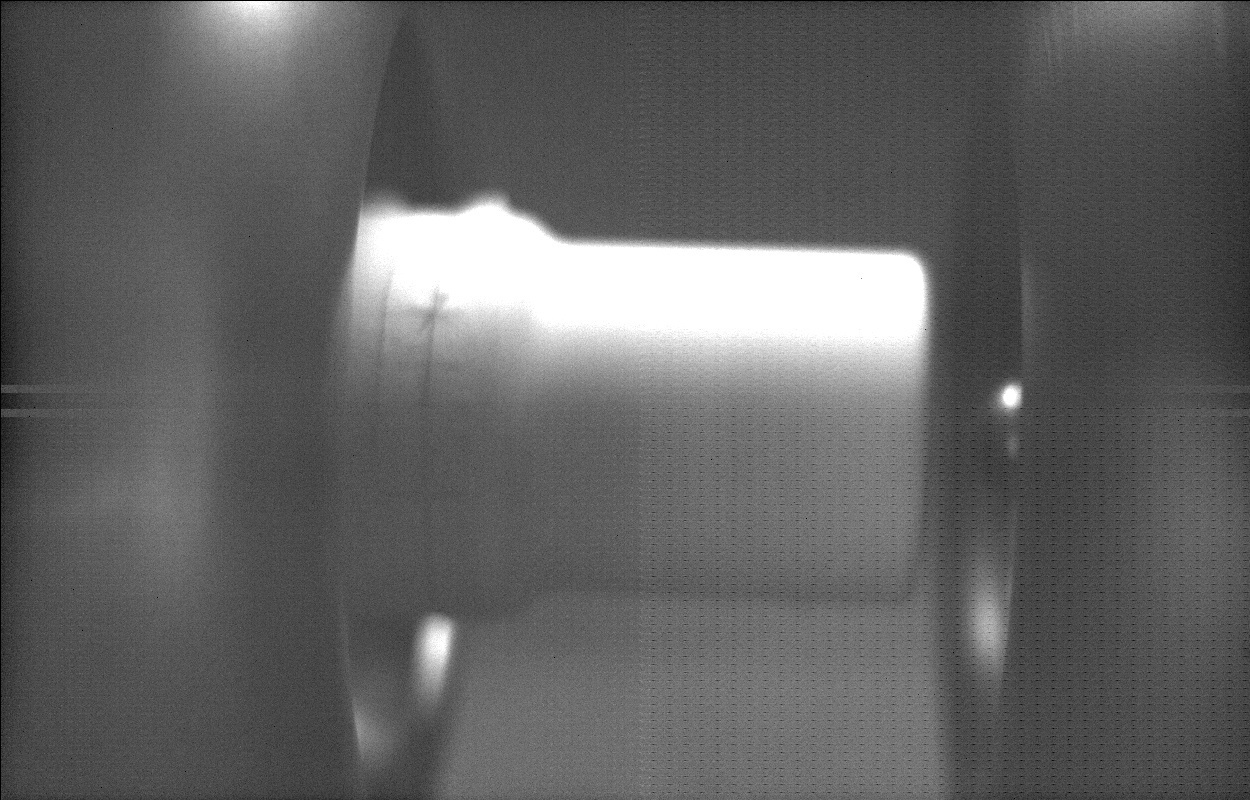

Supplement: S1 File — The relevant data can be obtained from the supporting information. The supporting information is mainly the image data analyzed in this paper. (ZIP) [file pone.0312253.s001.zip › supporting information/Nozzle/OPEN (22).jpg]

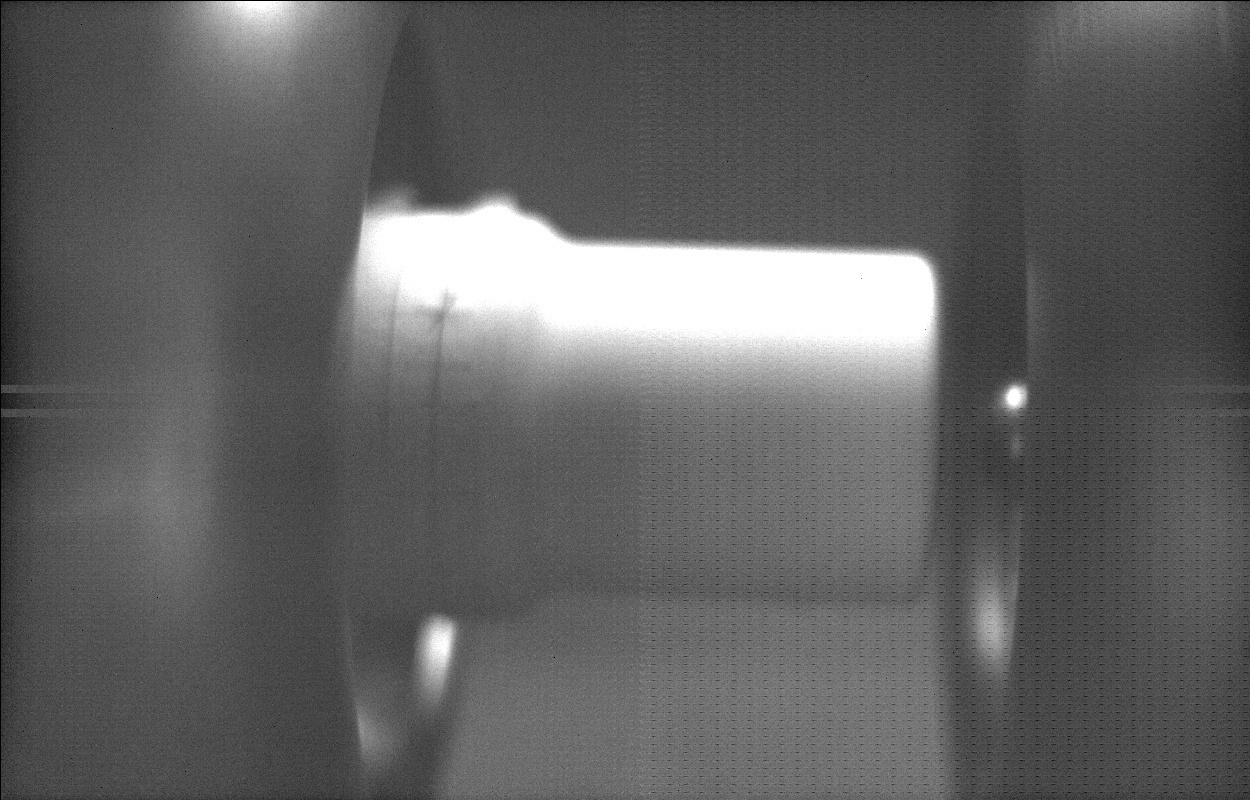

Supplement: S1 File — The relevant data can be obtained from the supporting information. The supporting information is mainly the image data analyzed in this paper. (ZIP) [file pone.0312253.s001.zip › supporting information/Nozzle/OPEN (23).jpg]

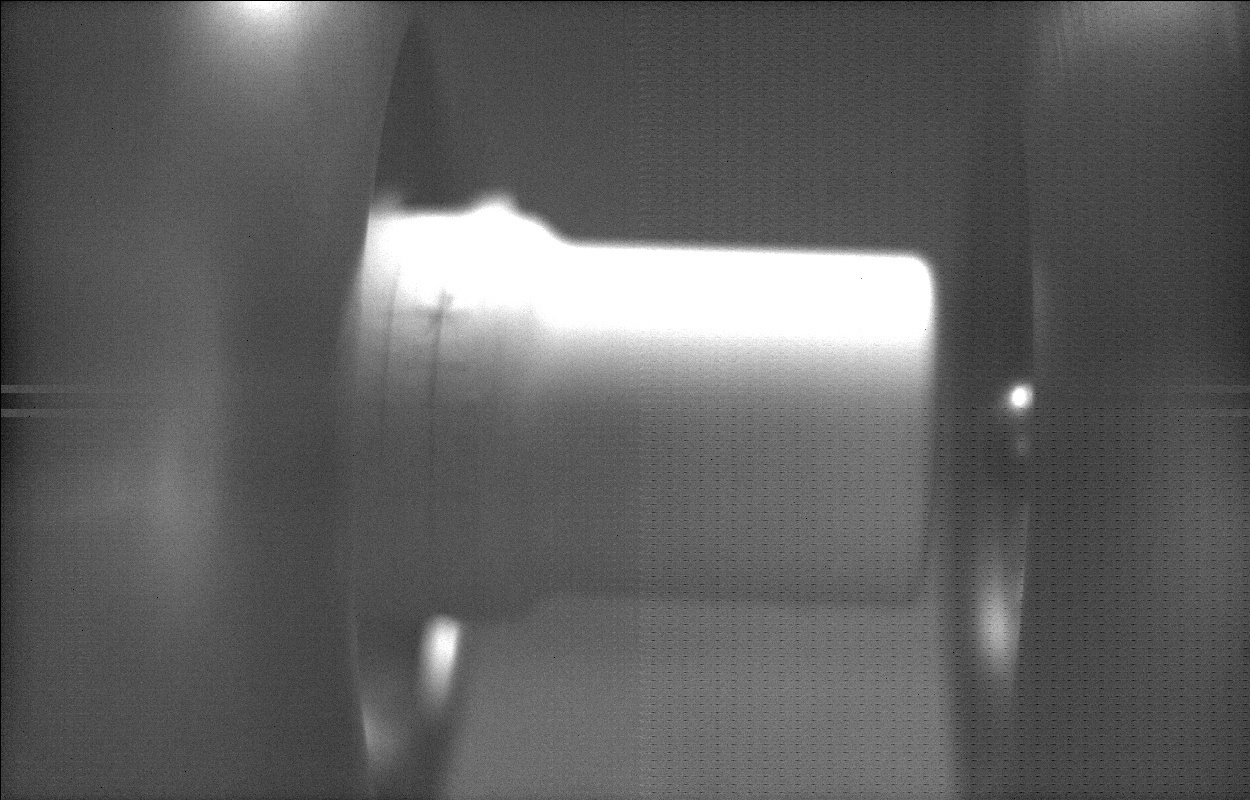

Supplement: S1 File — The relevant data can be obtained from the supporting information. The supporting information is mainly the image data analyzed in this paper. (ZIP) [file pone.0312253.s001.zip › supporting information/Nozzle/OPEN (24).jpg]

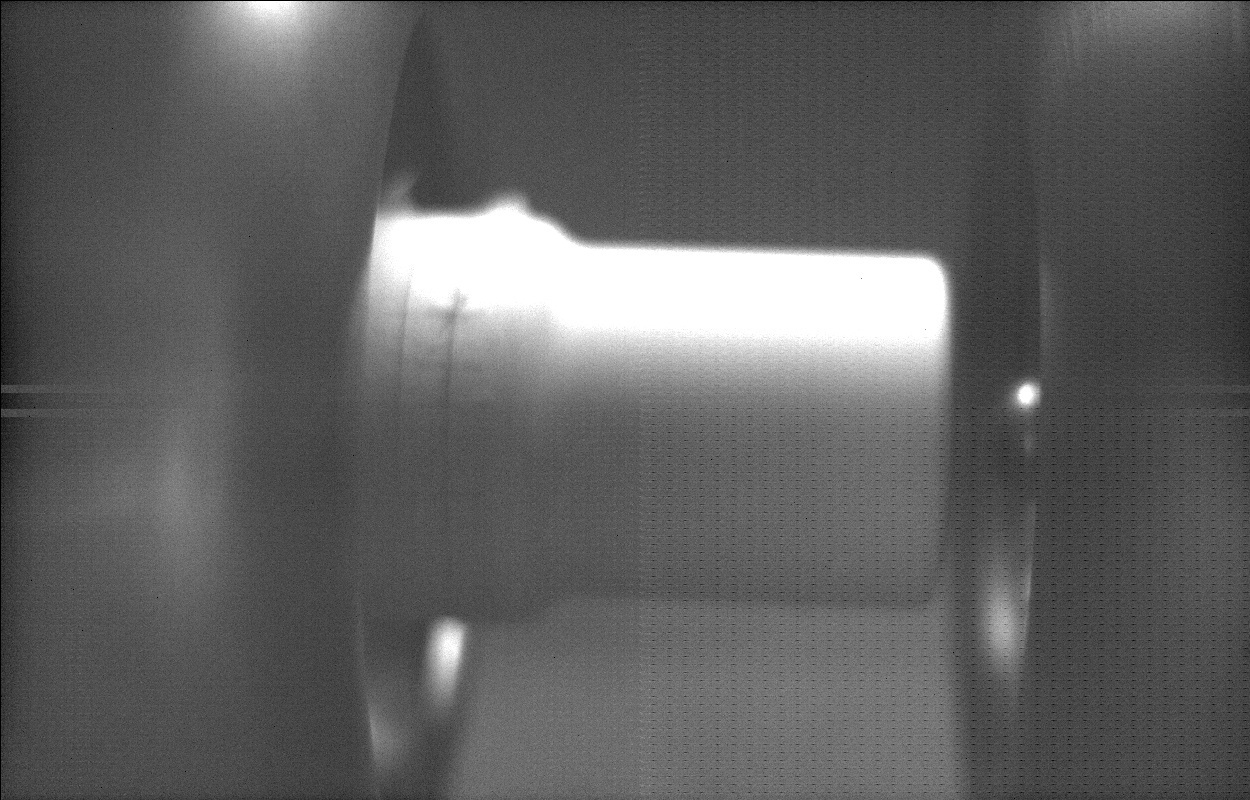

Supplement: S1 File — The relevant data can be obtained from the supporting information. The supporting information is mainly the image data analyzed in this paper. (ZIP) [file pone.0312253.s001.zip › supporting information/Nozzle/OPEN (25).jpg]

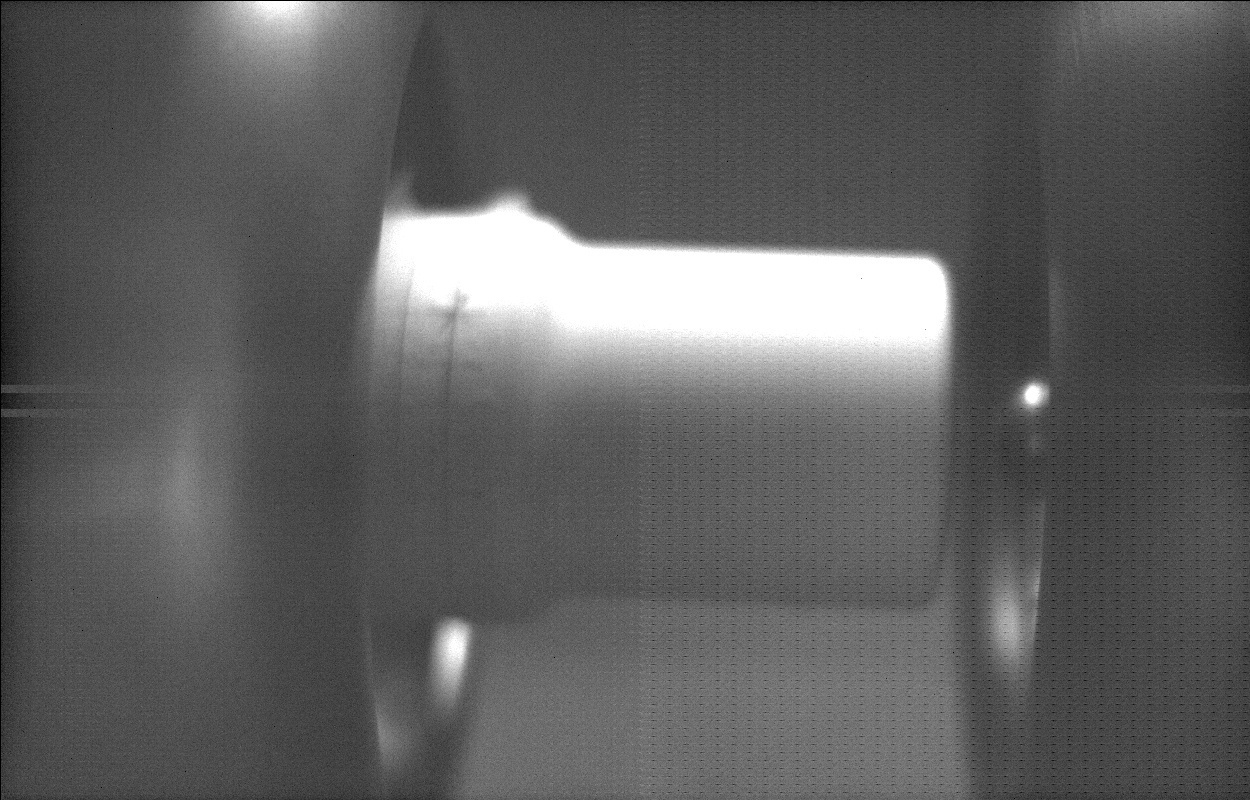

Supplement: S1 File — The relevant data can be obtained from the supporting information. The supporting information is mainly the image data analyzed in this paper. (ZIP) [file pone.0312253.s001.zip › supporting information/Nozzle/OPEN (26).jpg]

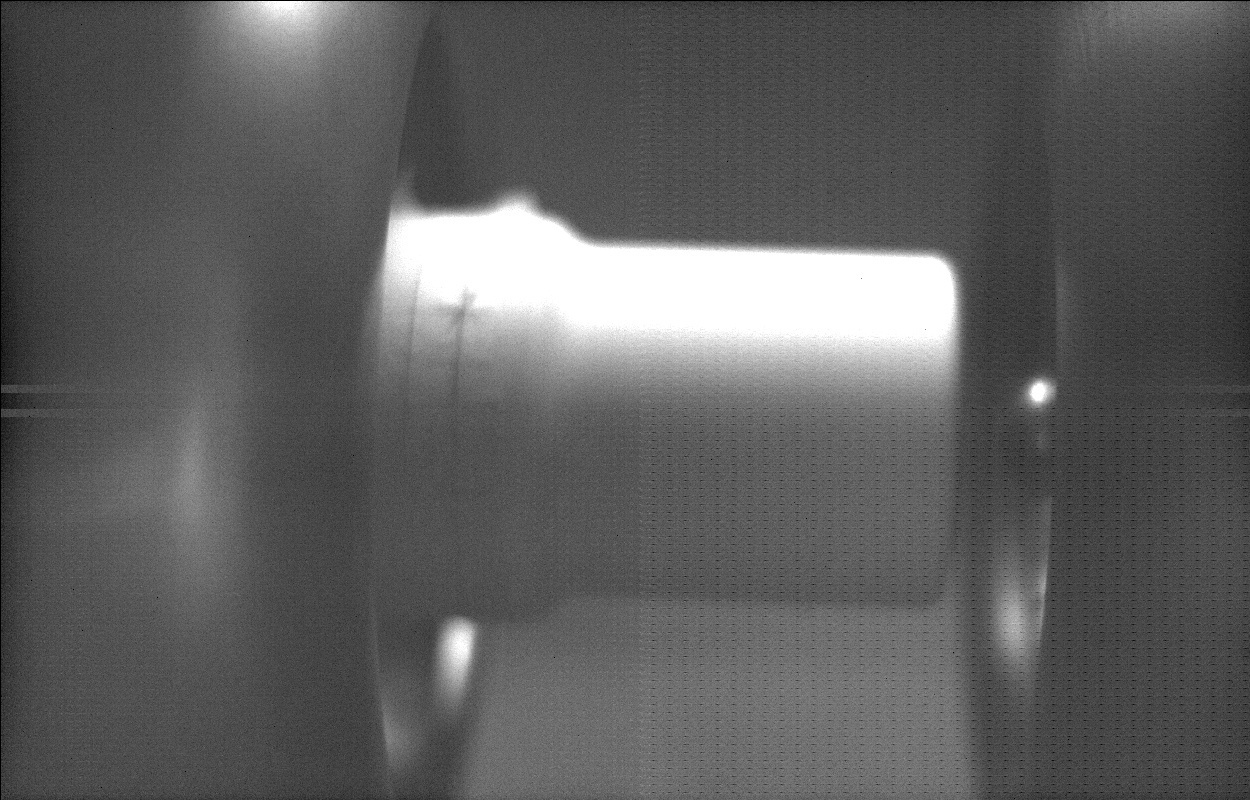

Supplement: S1 File — The relevant data can be obtained from the supporting information. The supporting information is mainly the image data analyzed in this paper. (ZIP) [file pone.0312253.s001.zip › supporting information/Nozzle/OPEN (27).jpg]

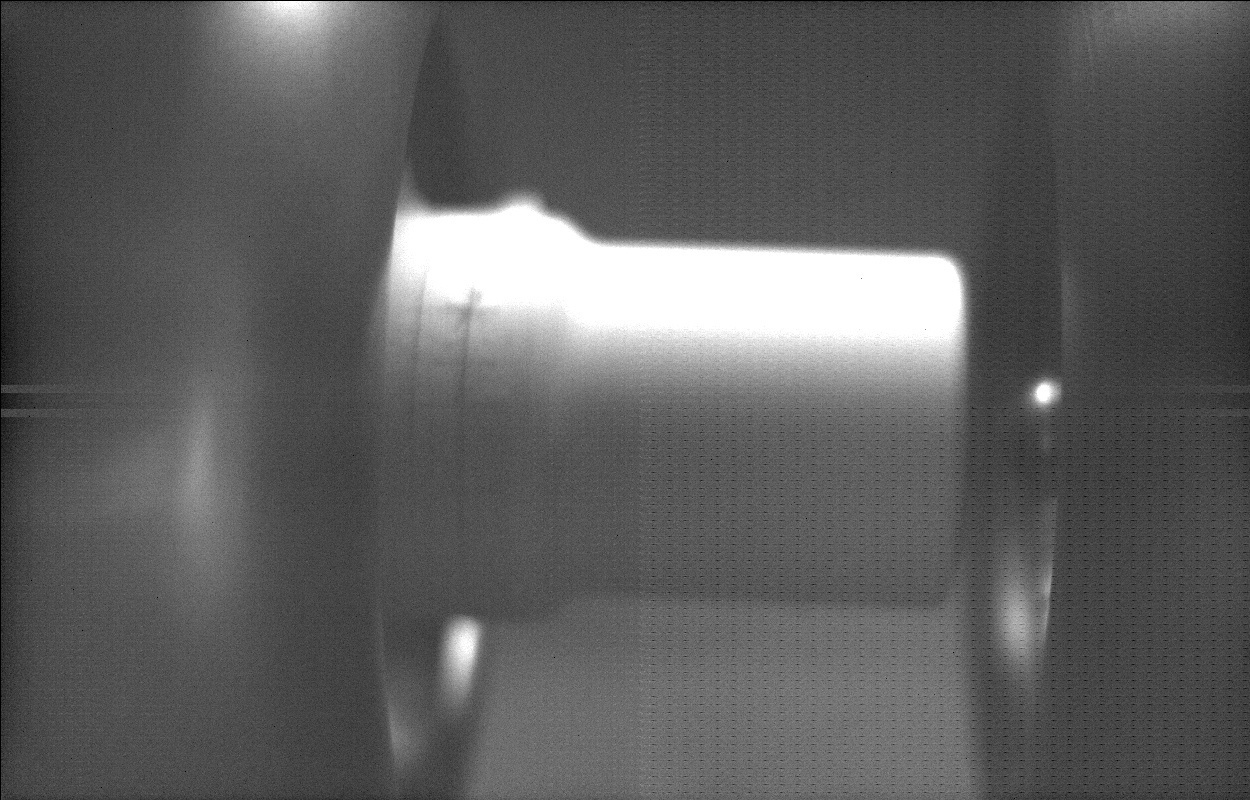

Supplement: S1 File — The relevant data can be obtained from the supporting information. The supporting information is mainly the image data analyzed in this paper. (ZIP) [file pone.0312253.s001.zip › supporting information/Nozzle/OPEN (28).jpg]

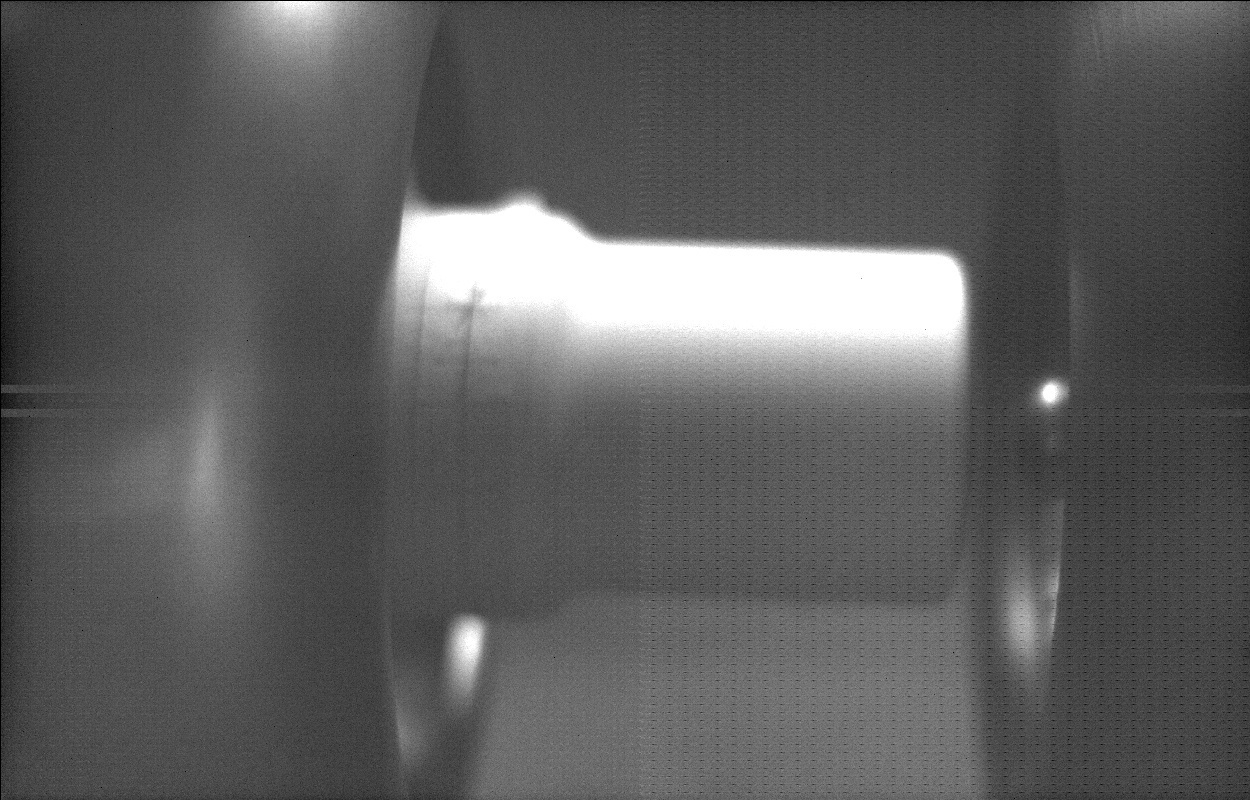

Supplement: S1 File — The relevant data can be obtained from the supporting information. The supporting information is mainly the image data analyzed in this paper. (ZIP) [file pone.0312253.s001.zip › supporting information/Nozzle/OPEN (29).jpg]

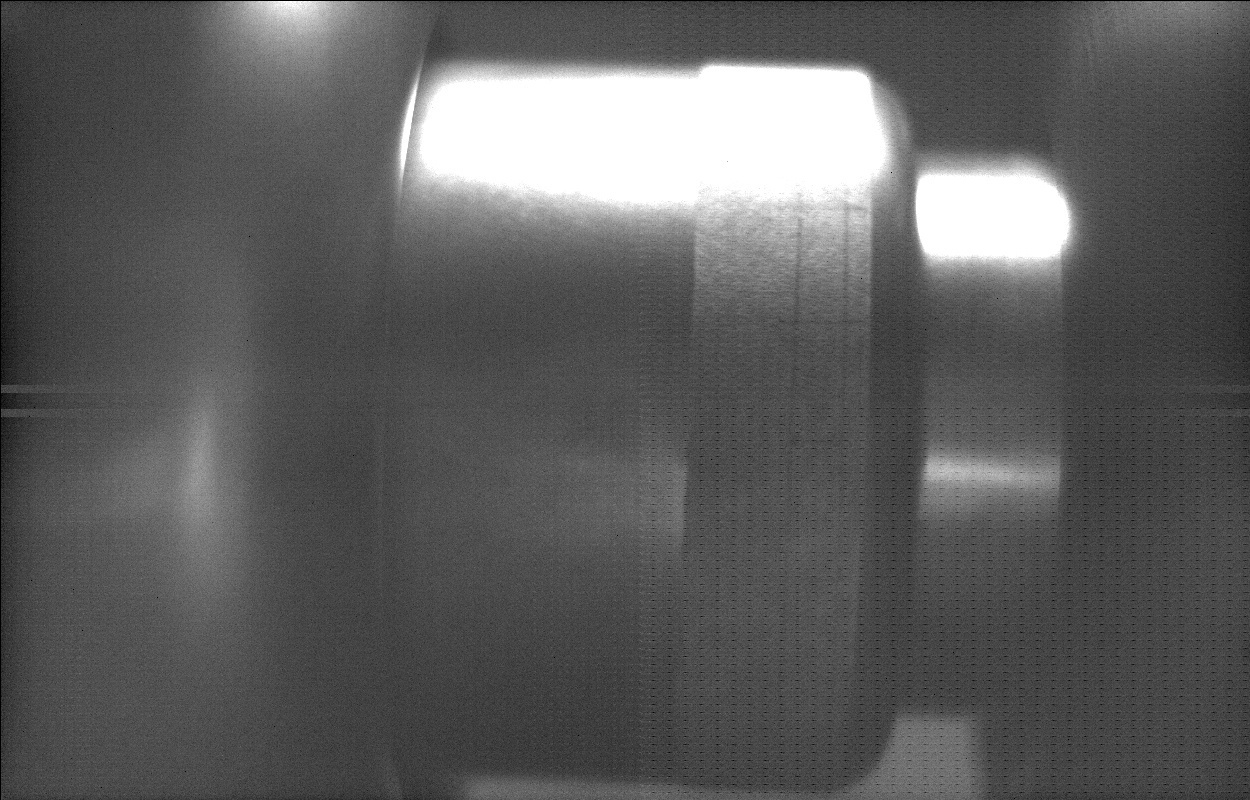

Supplement: S1 File — The relevant data can be obtained from the supporting information. The supporting information is mainly the image data analyzed in this paper. (ZIP) [file pone.0312253.s001.zip › supporting information/Nozzle/OPEN (3).jpg]

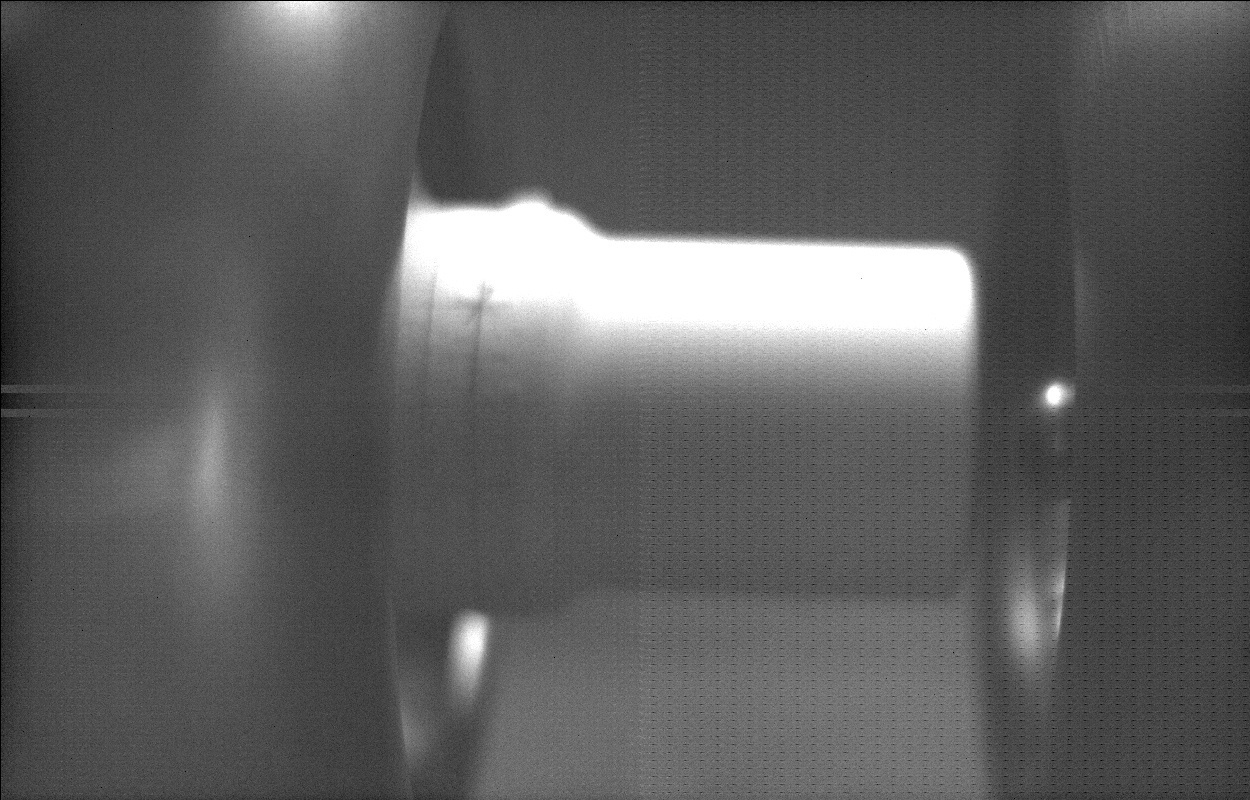

Supplement: S1 File — The relevant data can be obtained from the supporting information. The supporting information is mainly the image data analyzed in this paper. (ZIP) [file pone.0312253.s001.zip › supporting information/Nozzle/OPEN (30).jpg]

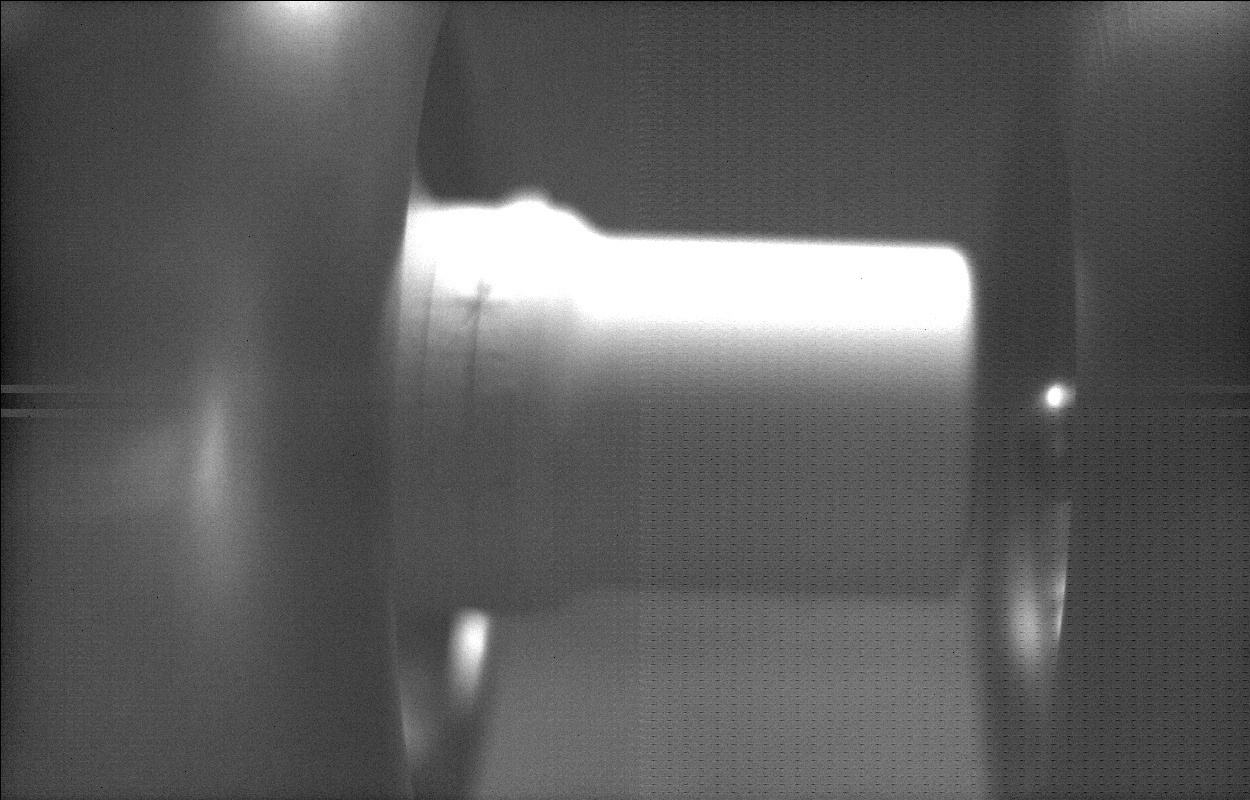

Supplement: S1 File — The relevant data can be obtained from the supporting information. The supporting information is mainly the image data analyzed in this paper. (ZIP) [file pone.0312253.s001.zip › supporting information/Nozzle/OPEN (31).jpg]

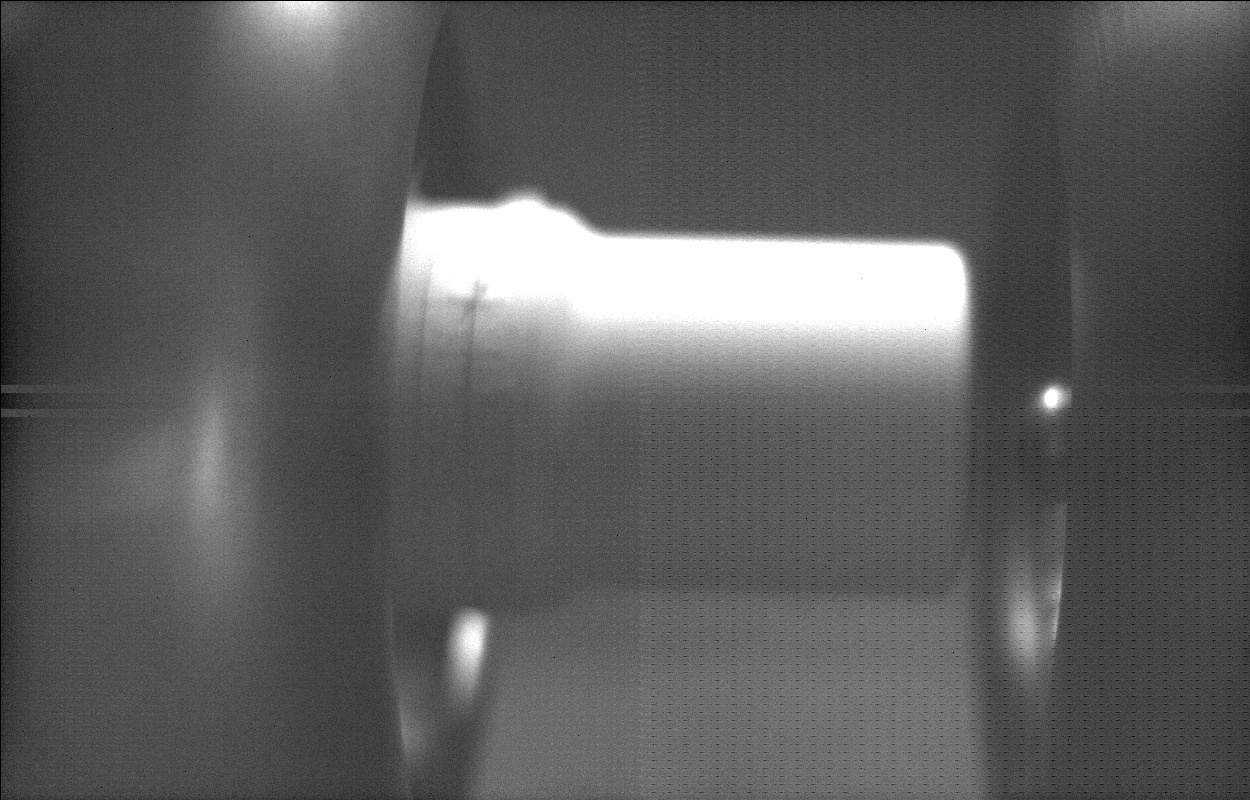

Supplement: S1 File — The relevant data can be obtained from the supporting information. The supporting information is mainly the image data analyzed in this paper. (ZIP) [file pone.0312253.s001.zip › supporting information/Nozzle/OPEN (32).jpg]

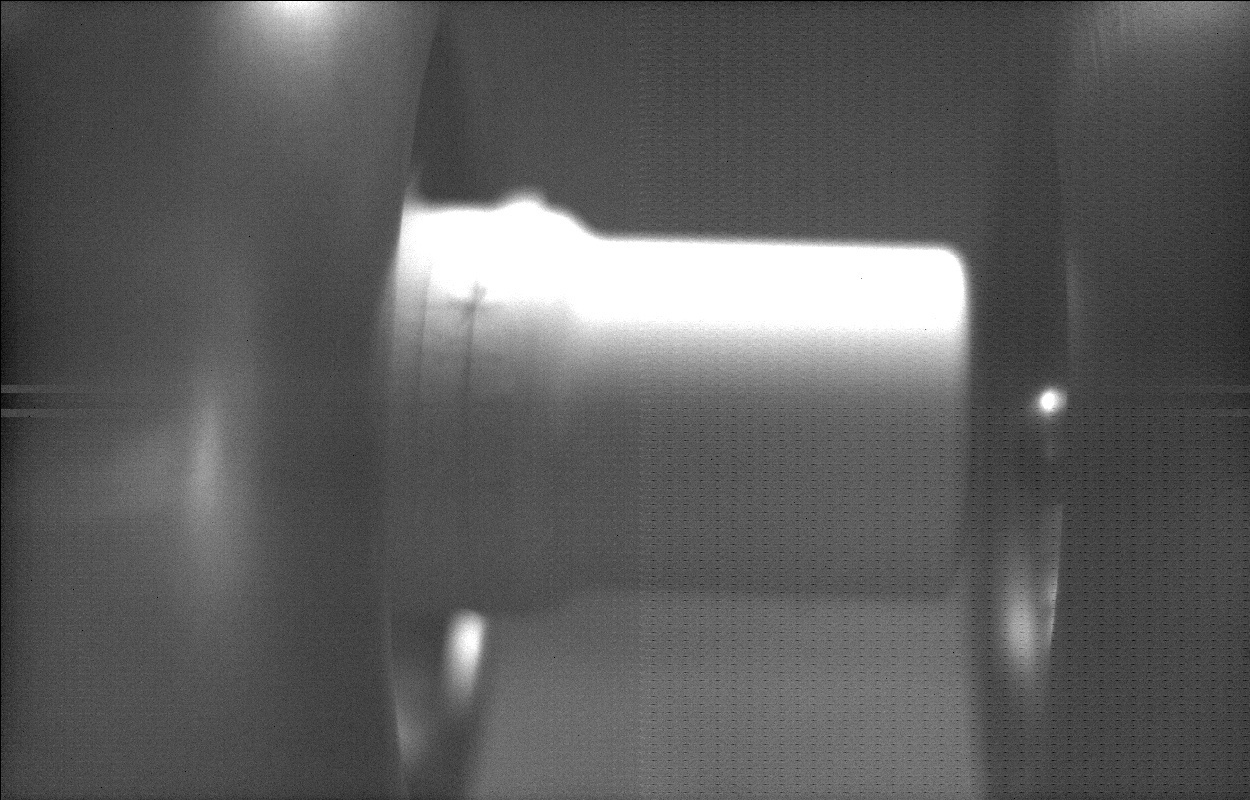

Supplement: S1 File — The relevant data can be obtained from the supporting information. The supporting information is mainly the image data analyzed in this paper. (ZIP) [file pone.0312253.s001.zip › supporting information/Nozzle/OPEN (33).jpg]

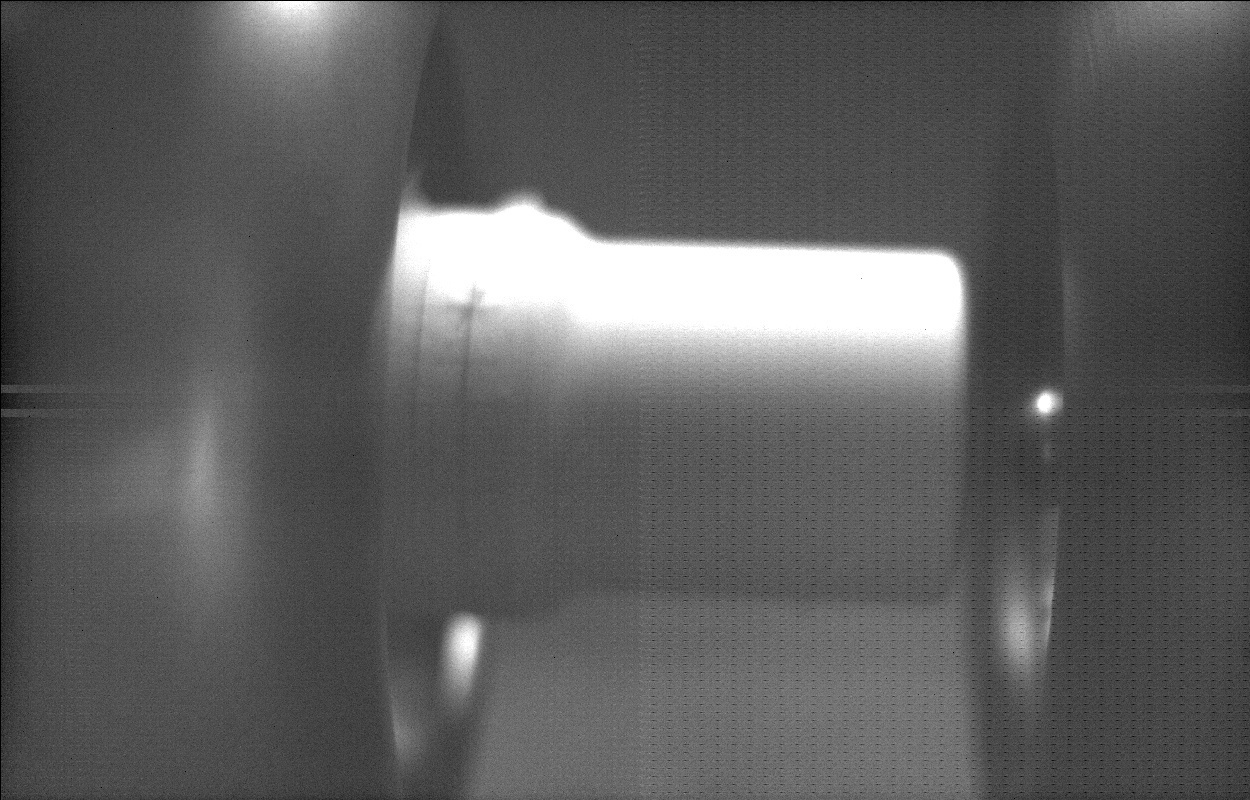

Supplement: S1 File — The relevant data can be obtained from the supporting information. The supporting information is mainly the image data analyzed in this paper. (ZIP) [file pone.0312253.s001.zip › supporting information/Nozzle/OPEN (34).jpg]

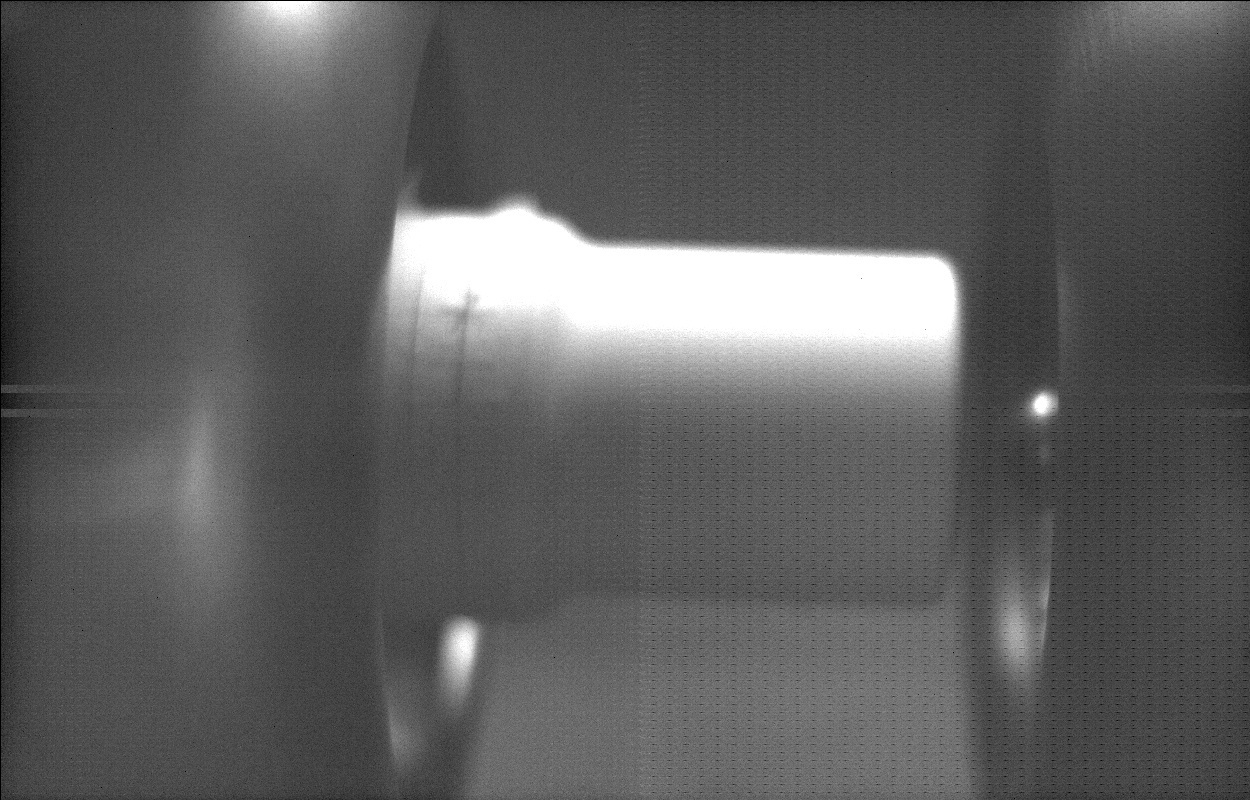

Supplement: S1 File — The relevant data can be obtained from the supporting information. The supporting information is mainly the image data analyzed in this paper. (ZIP) [file pone.0312253.s001.zip › supporting information/Nozzle/OPEN (35).jpg]

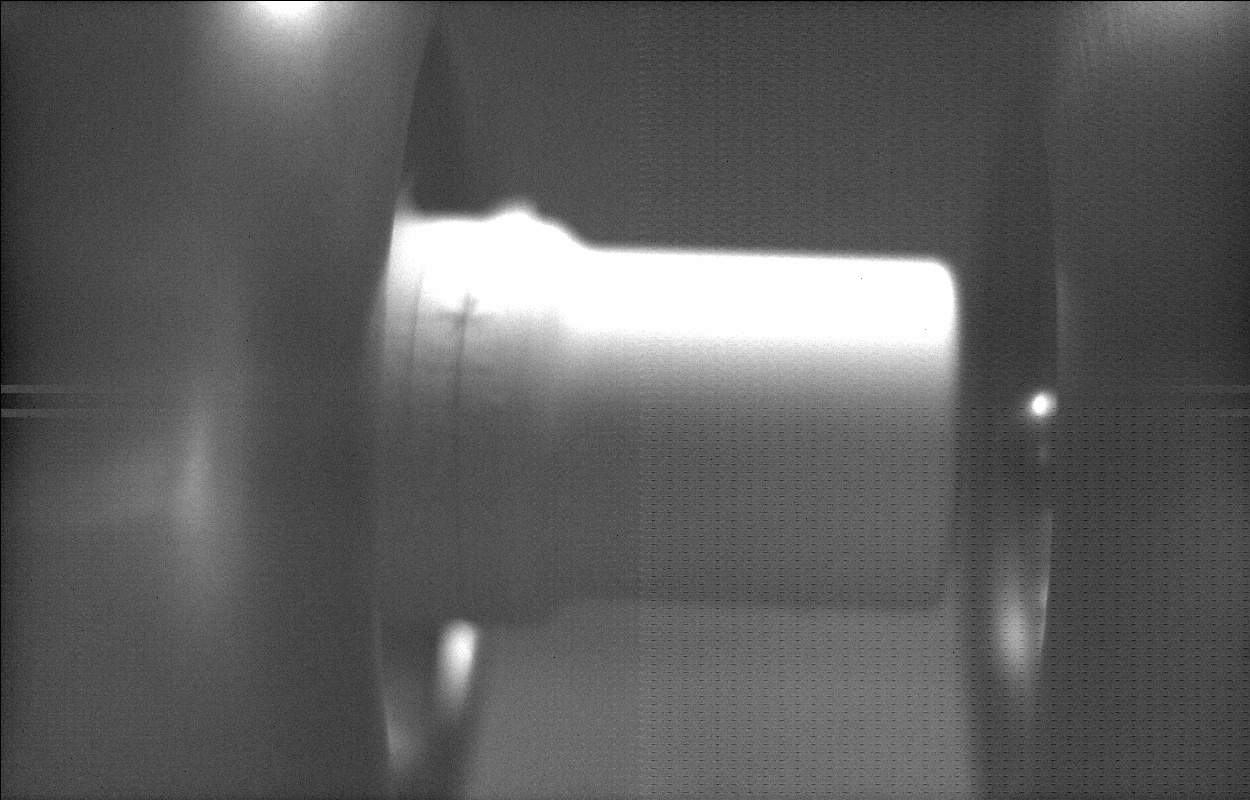

Supplement: S1 File — The relevant data can be obtained from the supporting information. The supporting information is mainly the image data analyzed in this paper. (ZIP) [file pone.0312253.s001.zip › supporting information/Nozzle/OPEN (36).jpg]

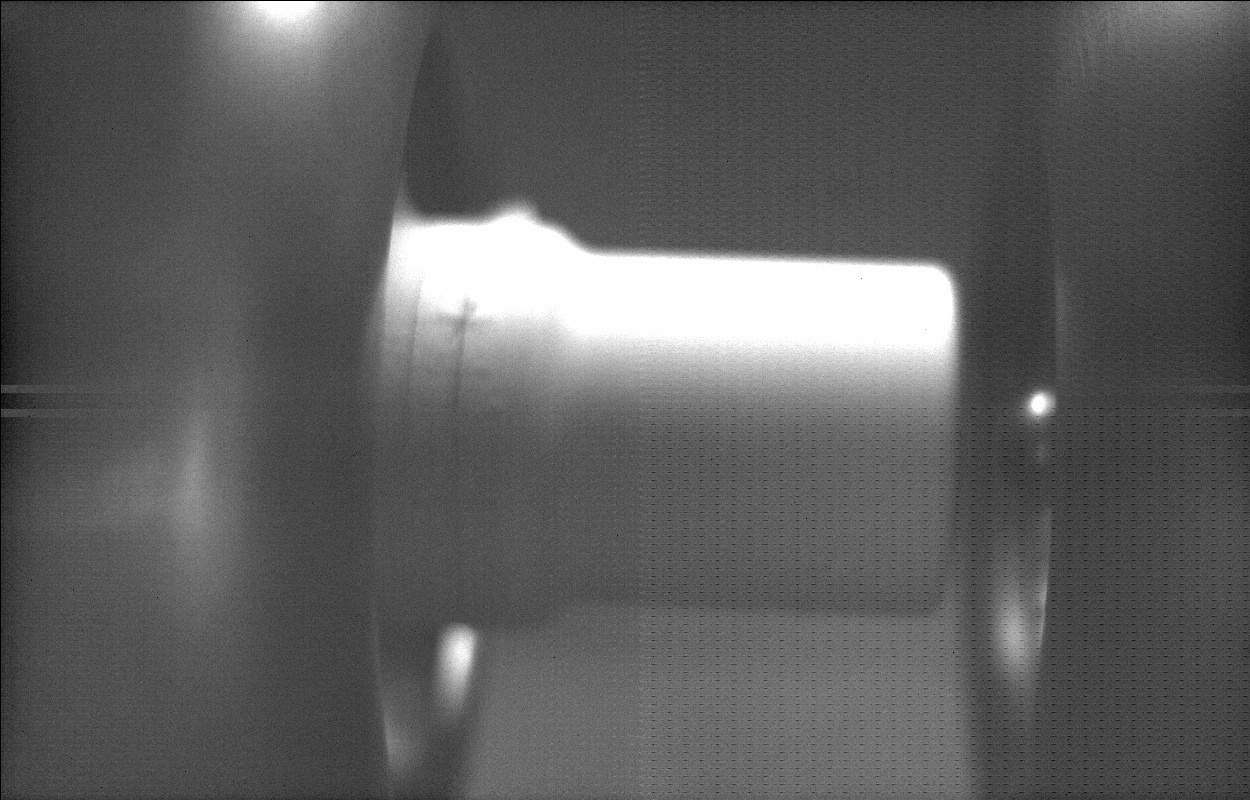

Supplement: S1 File — The relevant data can be obtained from the supporting information. The supporting information is mainly the image data analyzed in this paper. (ZIP) [file pone.0312253.s001.zip › supporting information/Nozzle/OPEN (37).jpg]

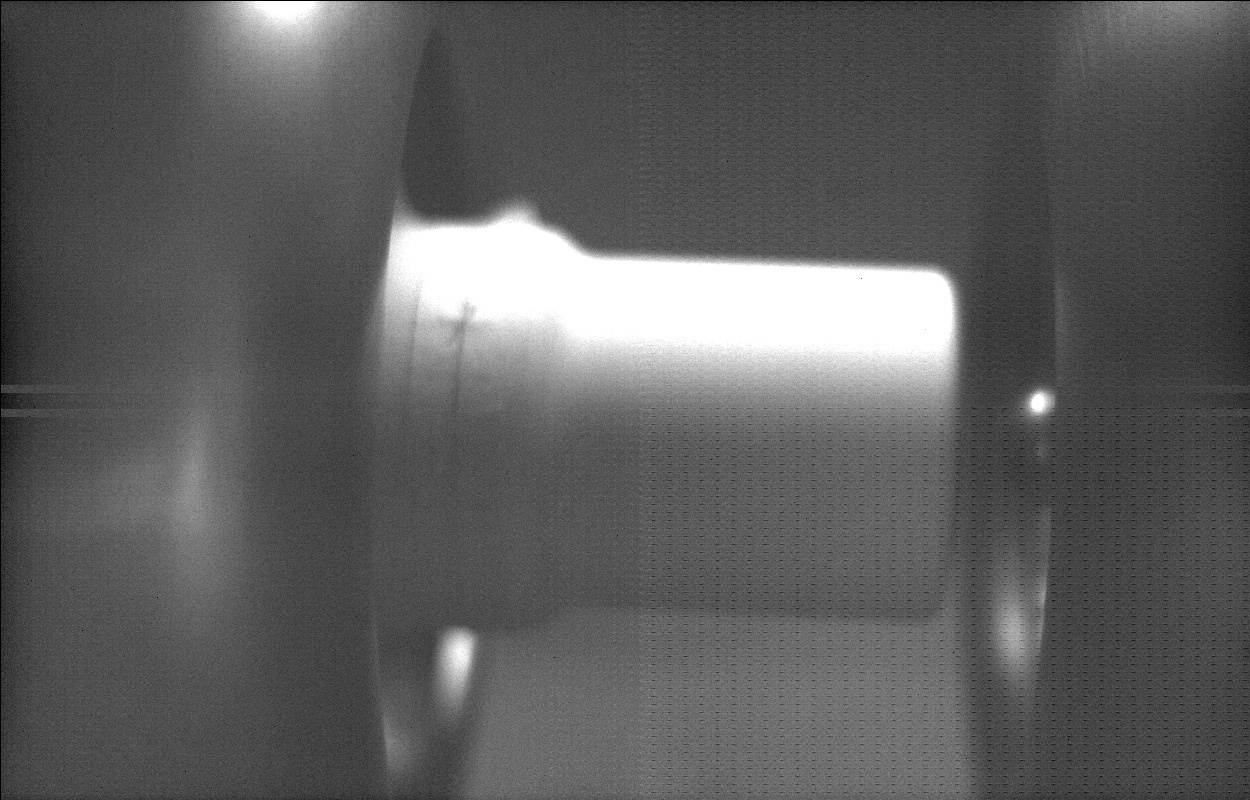

Supplement: S1 File — The relevant data can be obtained from the supporting information. The supporting information is mainly the image data analyzed in this paper. (ZIP) [file pone.0312253.s001.zip › supporting information/Nozzle/OPEN (38).jpg]

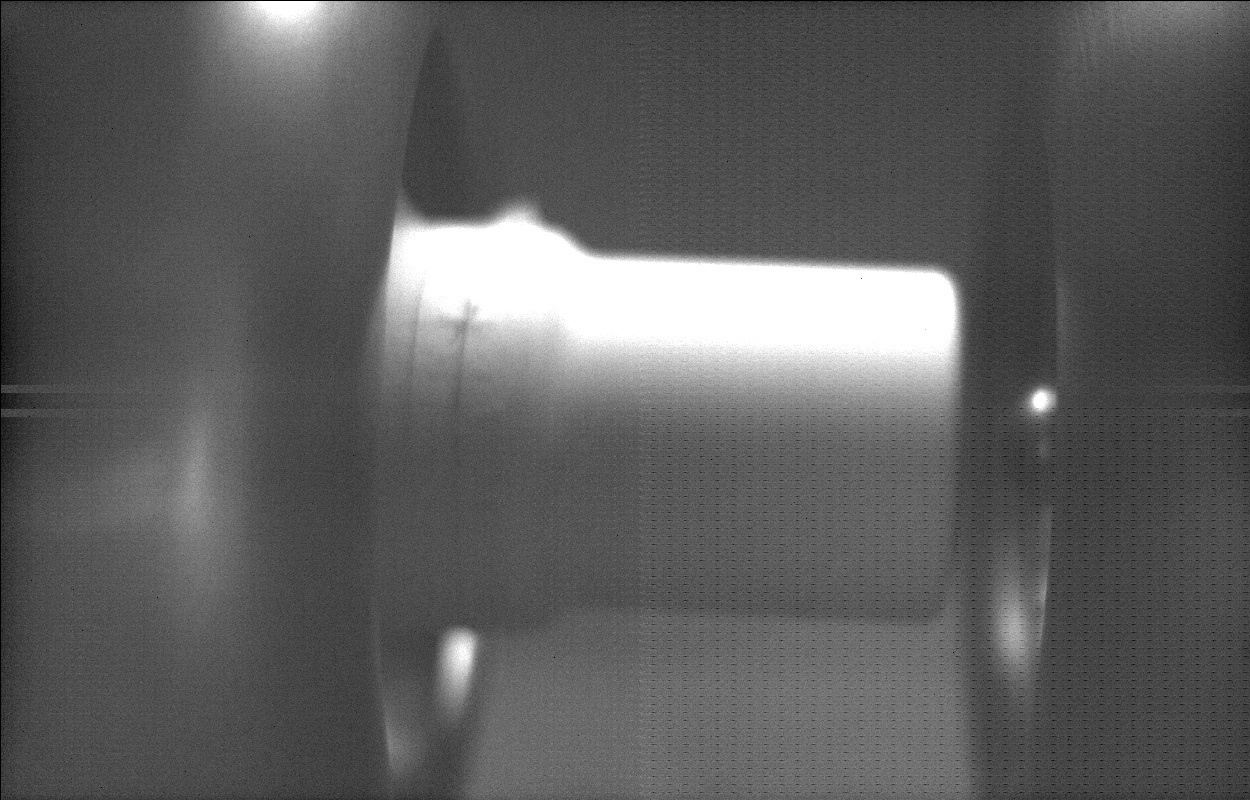

Supplement: S1 File — The relevant data can be obtained from the supporting information. The supporting information is mainly the image data analyzed in this paper. (ZIP) [file pone.0312253.s001.zip › supporting information/Nozzle/OPEN (39).jpg]

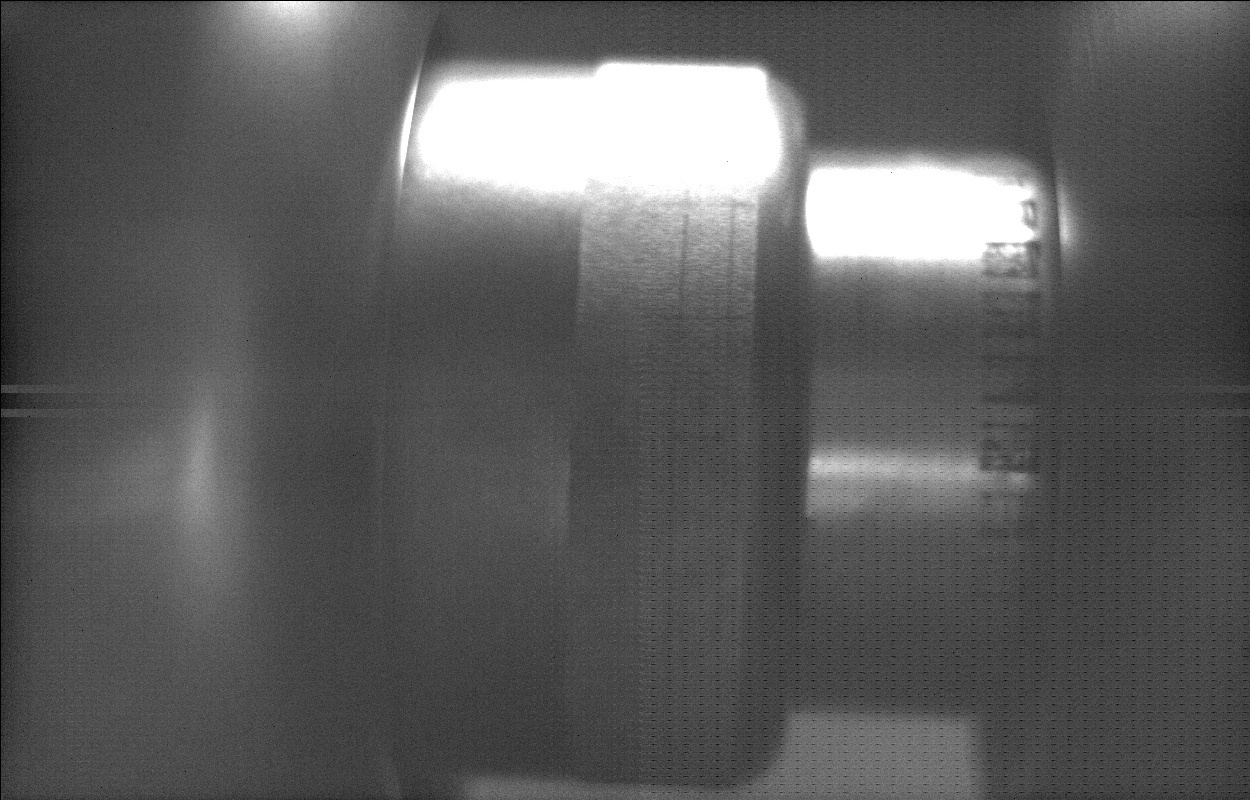

Supplement: S1 File — The relevant data can be obtained from the supporting information. The supporting information is mainly the image data analyzed in this paper. (ZIP) [file pone.0312253.s001.zip › supporting information/Nozzle/OPEN (4).jpg]

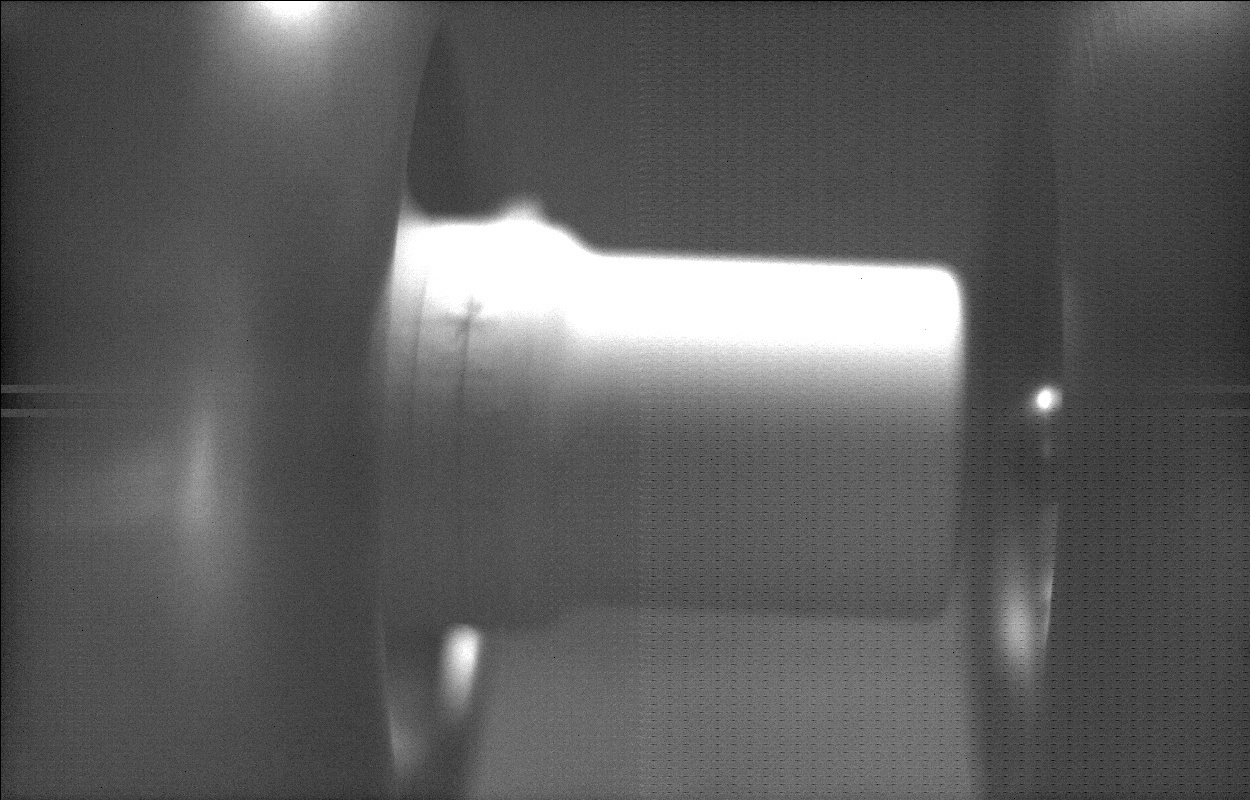

Supplement: S1 File — The relevant data can be obtained from the supporting information. The supporting information is mainly the image data analyzed in this paper. (ZIP) [file pone.0312253.s001.zip › supporting information/Nozzle/OPEN (40).jpg]

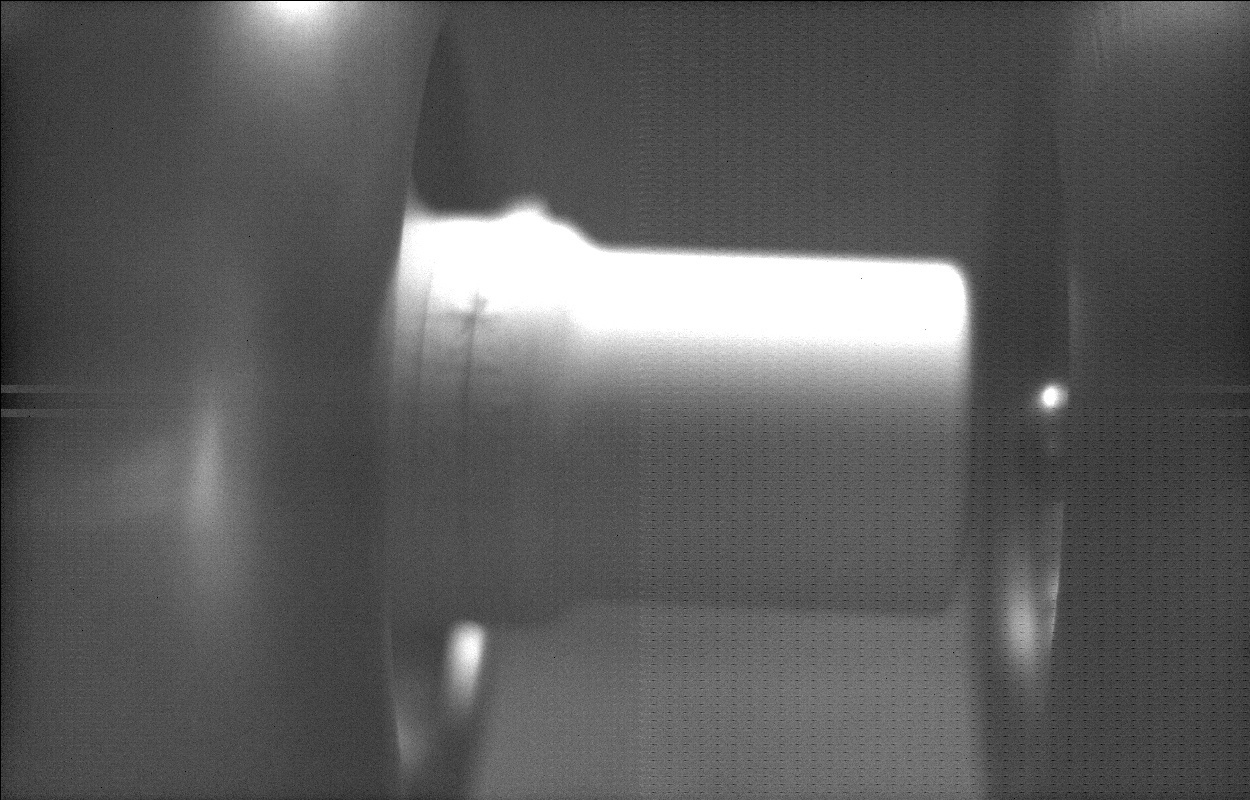

Supplement: S1 File — The relevant data can be obtained from the supporting information. The supporting information is mainly the image data analyzed in this paper. (ZIP) [file pone.0312253.s001.zip › supporting information/Nozzle/OPEN (41).jpg]

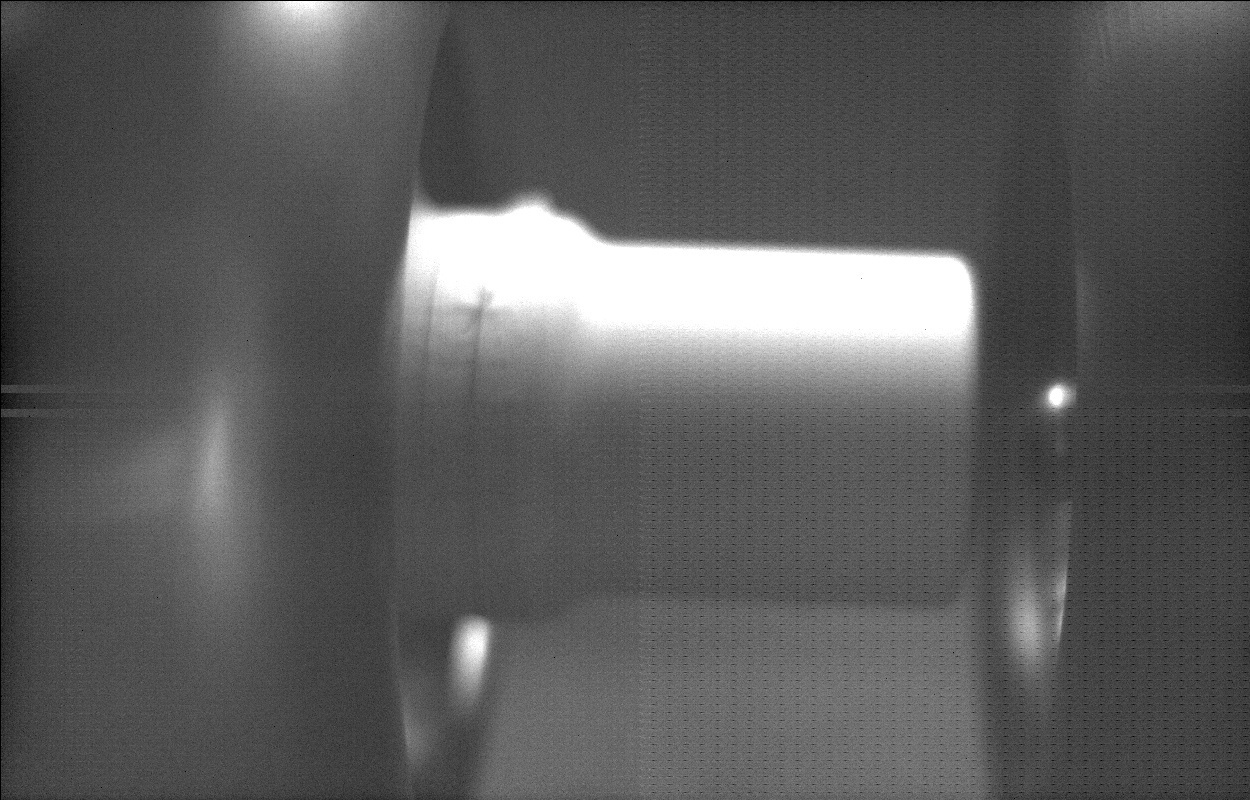

Supplement: S1 File — The relevant data can be obtained from the supporting information. The supporting information is mainly the image data analyzed in this paper. (ZIP) [file pone.0312253.s001.zip › supporting information/Nozzle/OPEN (42).jpg]

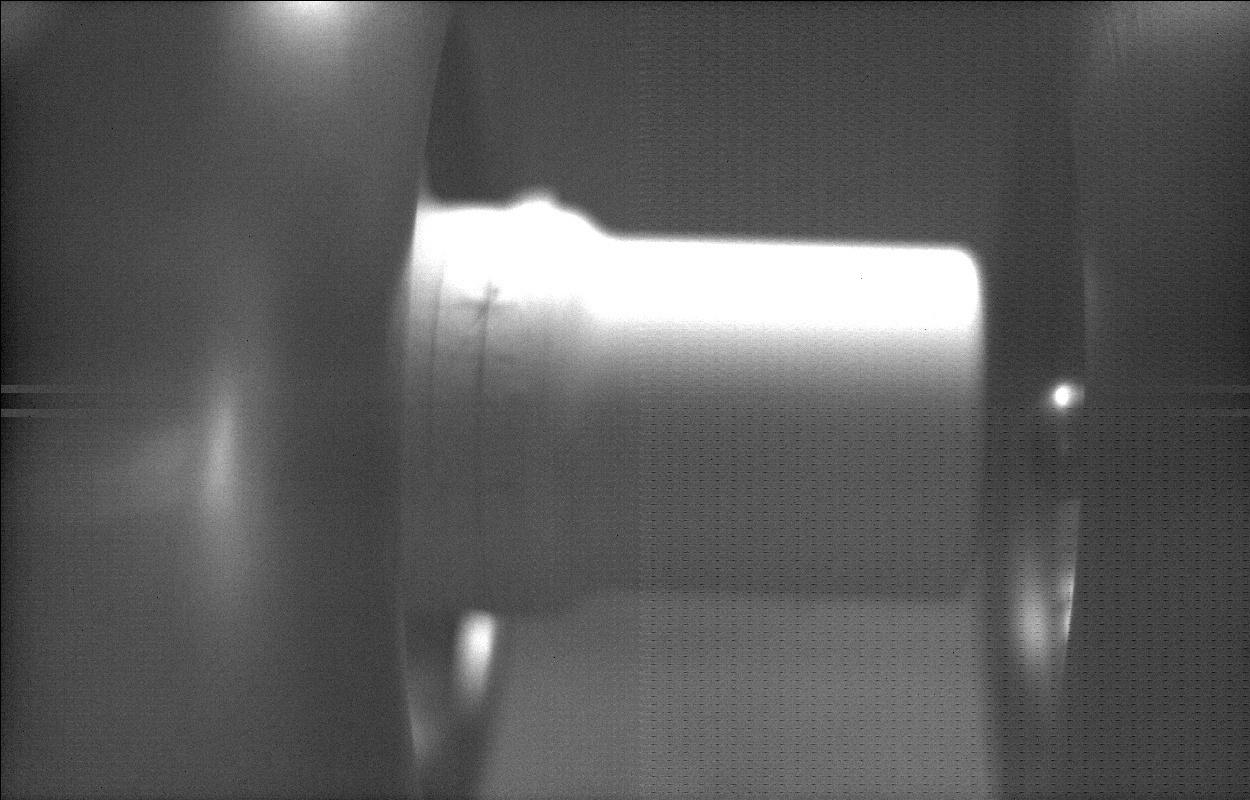

Supplement: S1 File — The relevant data can be obtained from the supporting information. The supporting information is mainly the image data analyzed in this paper. (ZIP) [file pone.0312253.s001.zip › supporting information/Nozzle/OPEN (43).jpg]

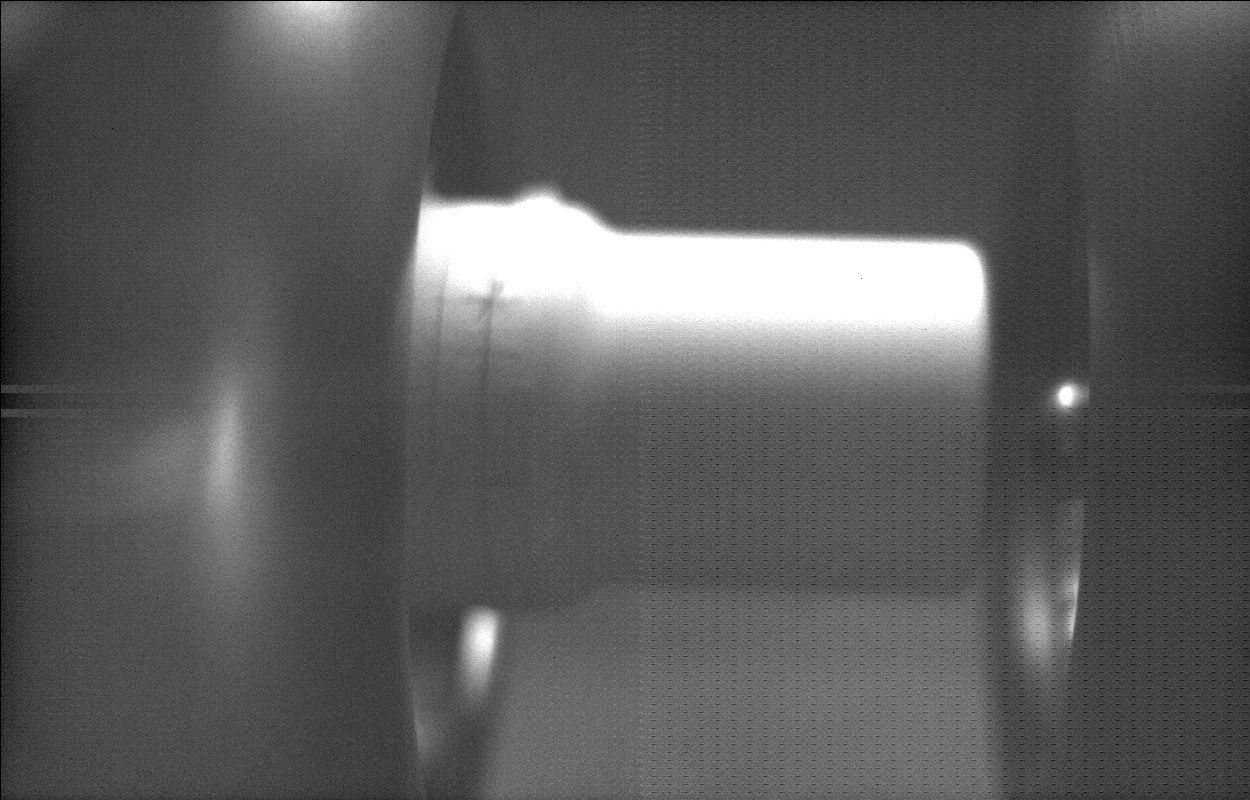

Supplement: S1 File — The relevant data can be obtained from the supporting information. The supporting information is mainly the image data analyzed in this paper. (ZIP) [file pone.0312253.s001.zip › supporting information/Nozzle/OPEN (44).jpg]

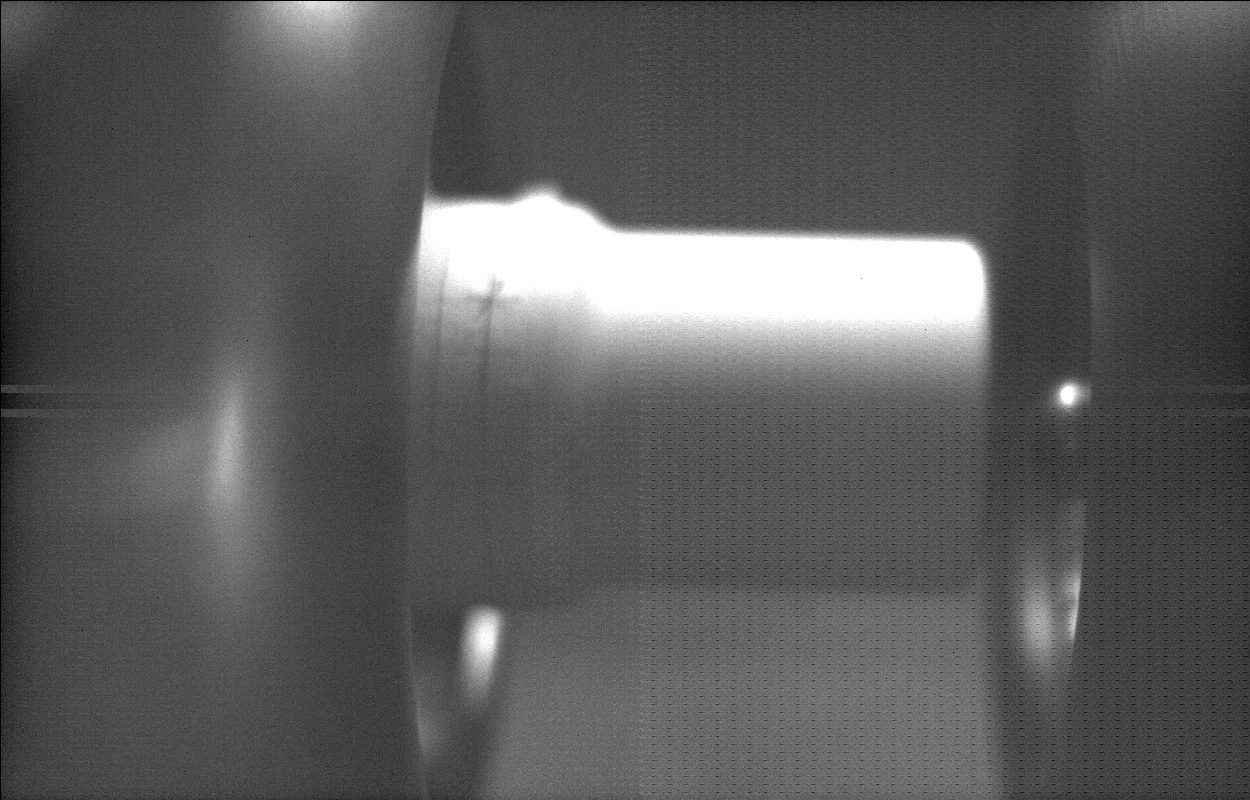

Supplement: S1 File — The relevant data can be obtained from the supporting information. The supporting information is mainly the image data analyzed in this paper. (ZIP) [file pone.0312253.s001.zip › supporting information/Nozzle/OPEN (45).jpg]

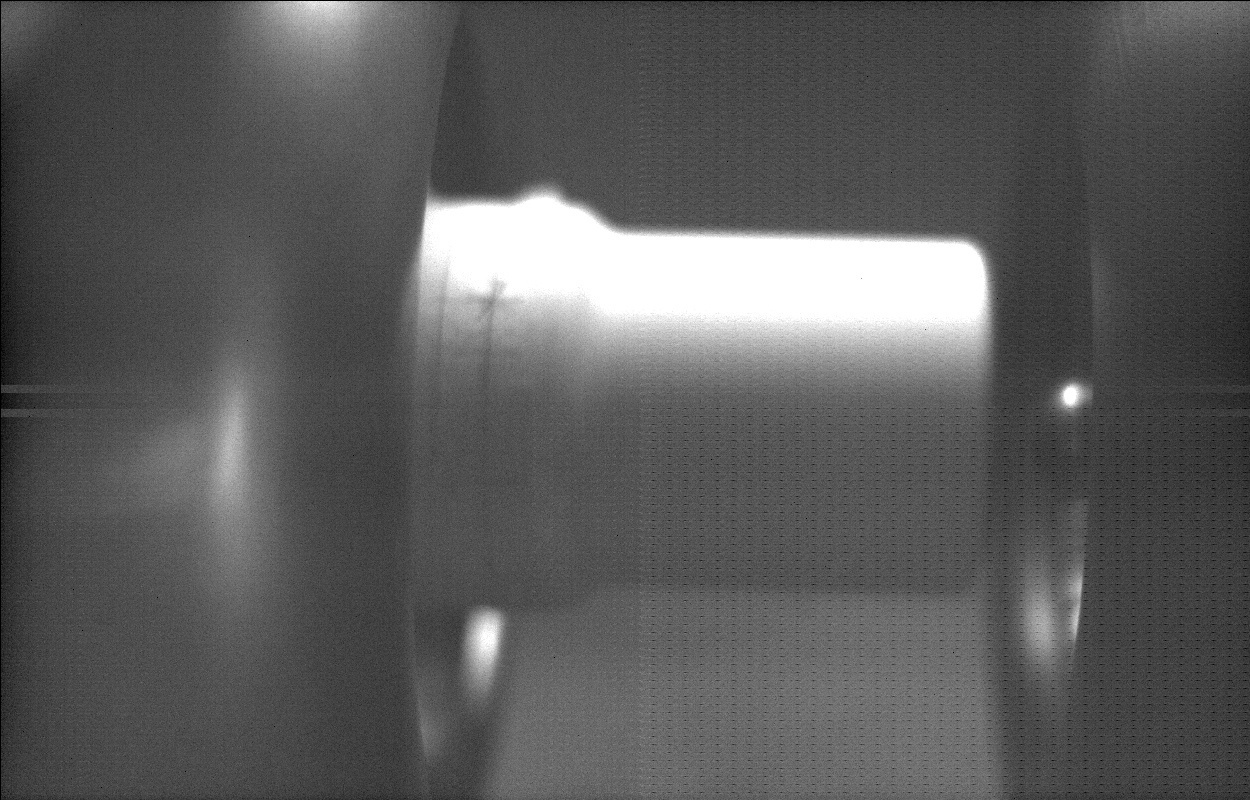

Supplement: S1 File — The relevant data can be obtained from the supporting information. The supporting information is mainly the image data analyzed in this paper. (ZIP) [file pone.0312253.s001.zip › supporting information/Nozzle/OPEN (46).jpg]

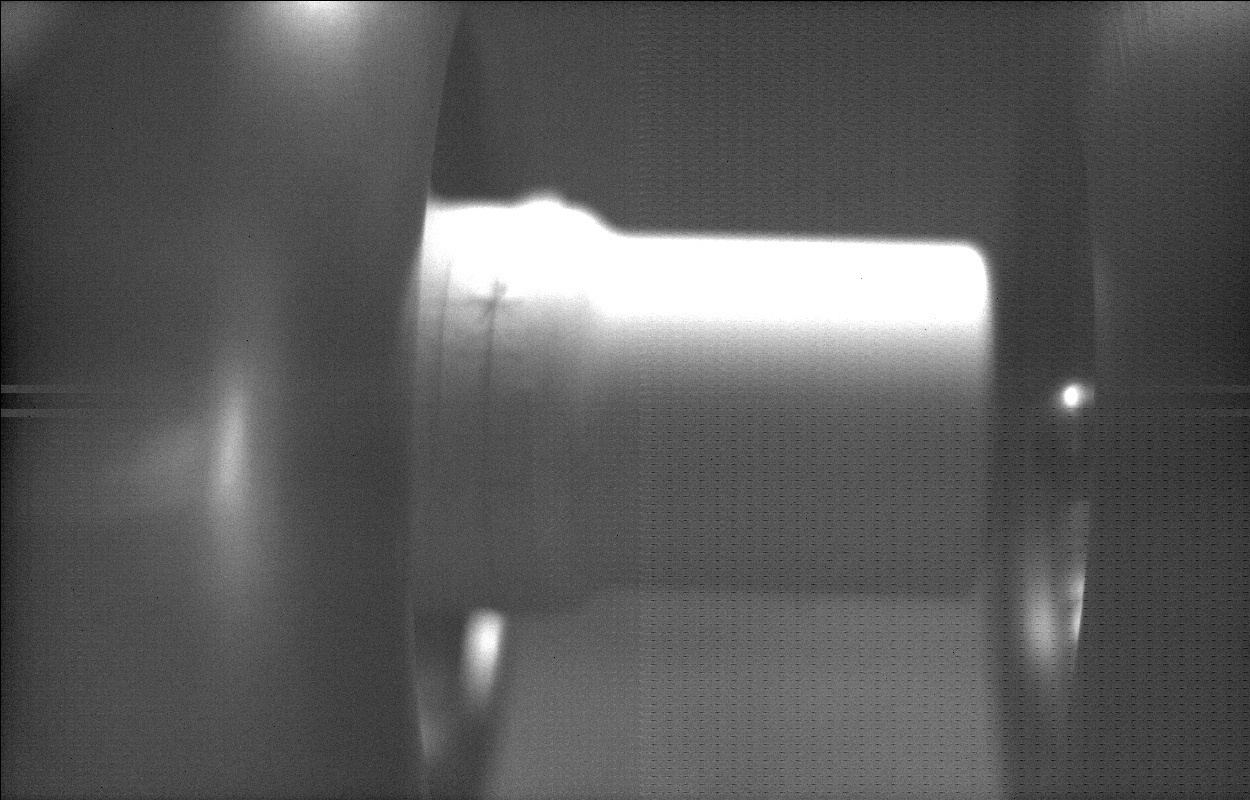

Supplement: S1 File — The relevant data can be obtained from the supporting information. The supporting information is mainly the image data analyzed in this paper. (ZIP) [file pone.0312253.s001.zip › supporting information/Nozzle/OPEN (47).jpg]

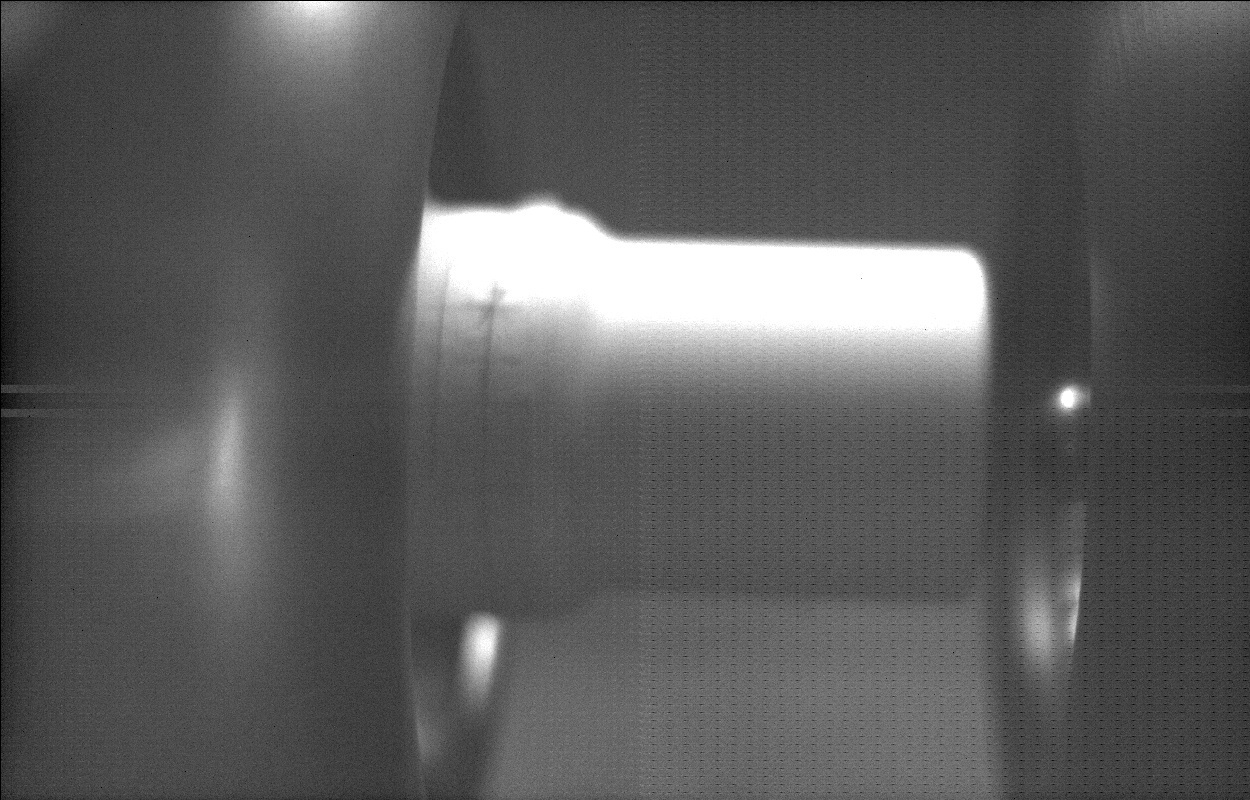

Supplement: S1 File — The relevant data can be obtained from the supporting information. The supporting information is mainly the image data analyzed in this paper. (ZIP) [file pone.0312253.s001.zip › supporting information/Nozzle/OPEN (48).jpg]

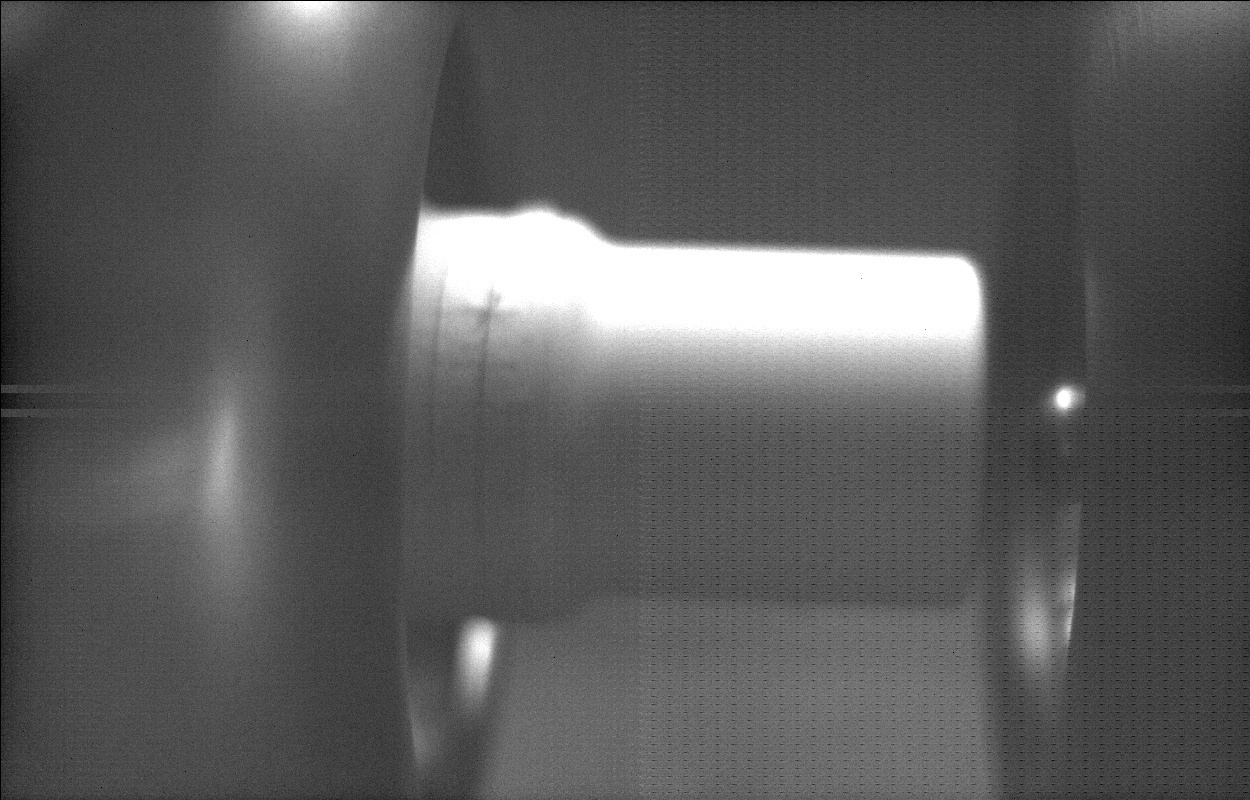

Supplement: S1 File — The relevant data can be obtained from the supporting information. The supporting information is mainly the image data analyzed in this paper. (ZIP) [file pone.0312253.s001.zip › supporting information/Nozzle/OPEN (49).jpg]

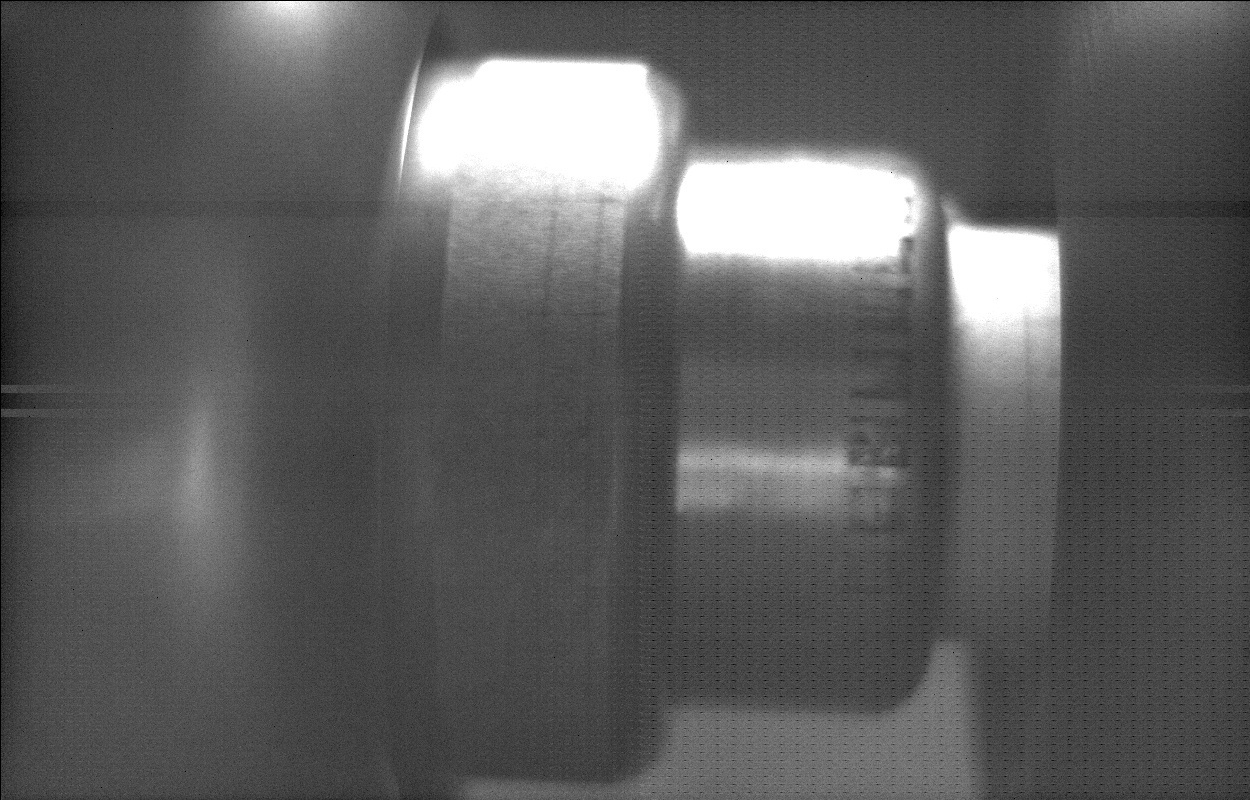

Supplement: S1 File — The relevant data can be obtained from the supporting information. The supporting information is mainly the image data analyzed in this paper. (ZIP) [file pone.0312253.s001.zip › supporting information/Nozzle/OPEN (5).jpg]

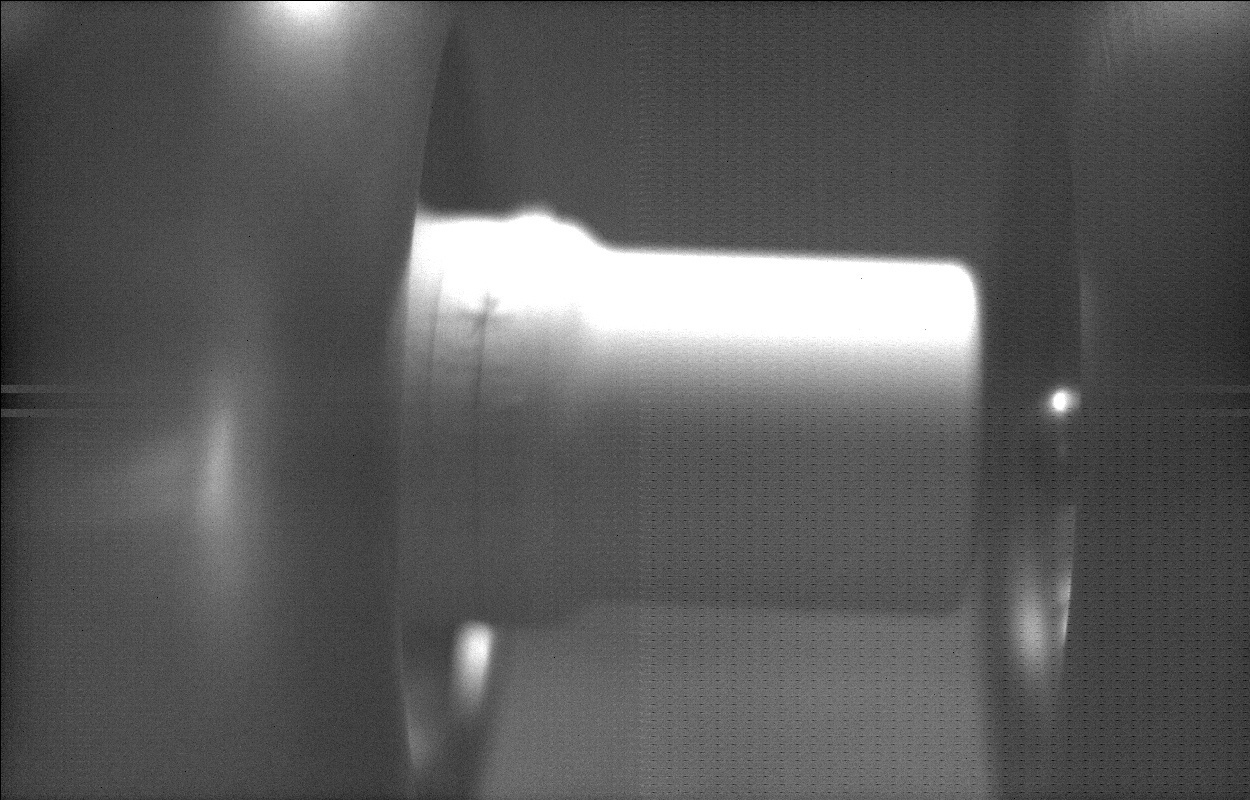

Supplement: S1 File — The relevant data can be obtained from the supporting information. The supporting information is mainly the image data analyzed in this paper. (ZIP) [file pone.0312253.s001.zip › supporting information/Nozzle/OPEN (50).jpg]

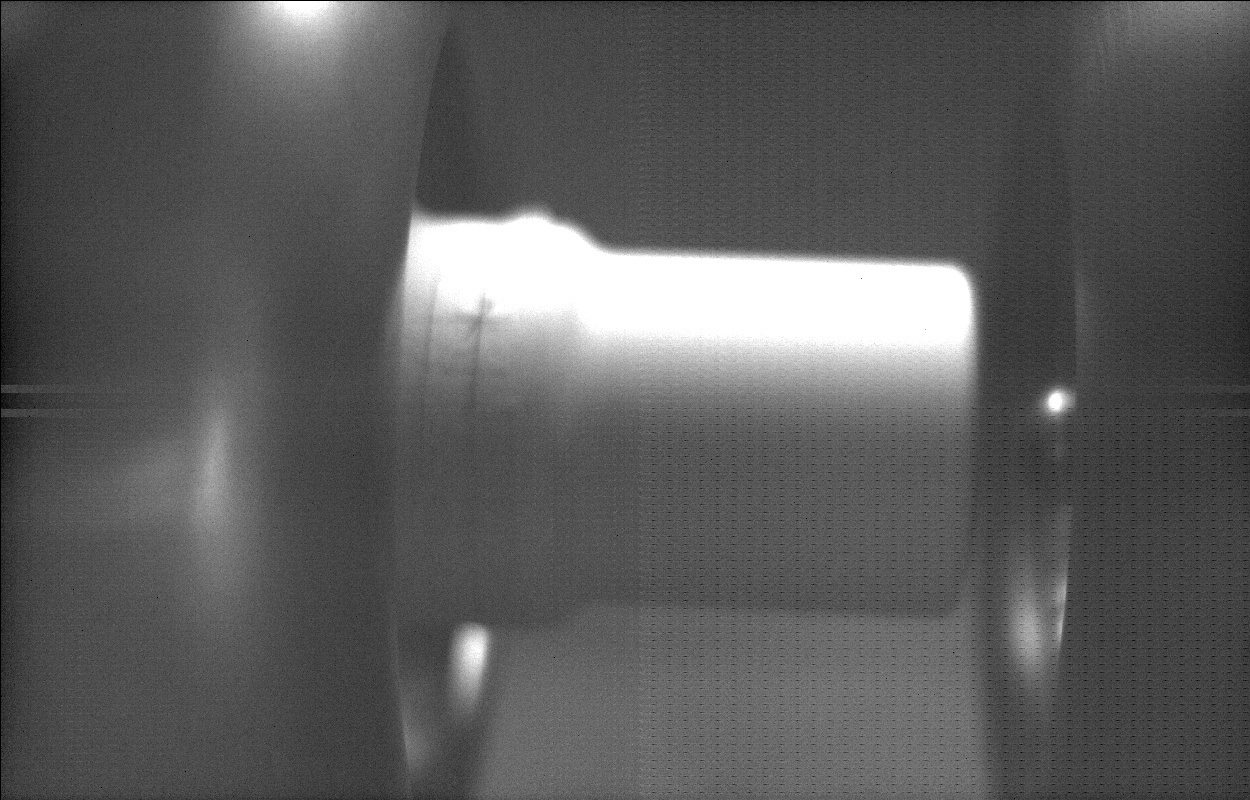

Supplement: S1 File — The relevant data can be obtained from the supporting information. The supporting information is mainly the image data analyzed in this paper. (ZIP) [file pone.0312253.s001.zip › supporting information/Nozzle/OPEN (51).jpg]

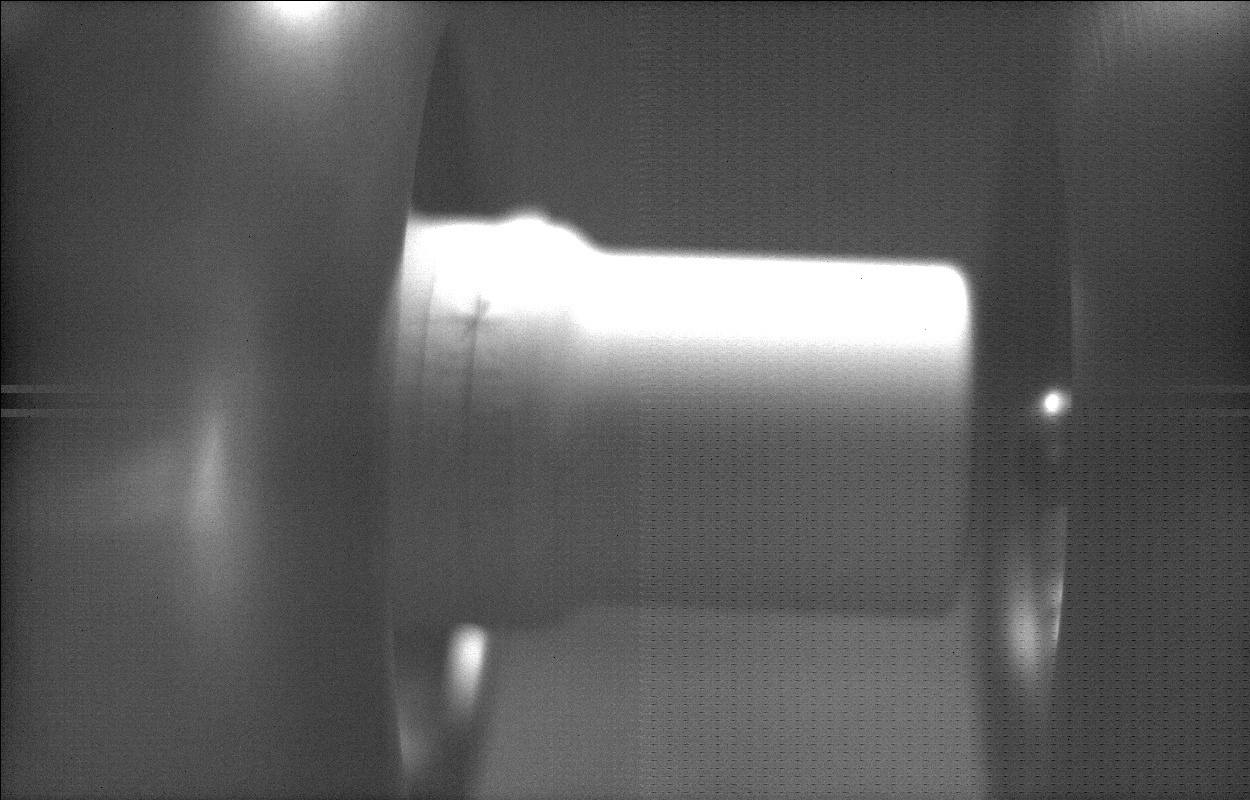

Supplement: S1 File — The relevant data can be obtained from the supporting information. The supporting information is mainly the image data analyzed in this paper. (ZIP) [file pone.0312253.s001.zip › supporting information/Nozzle/OPEN (52).jpg]

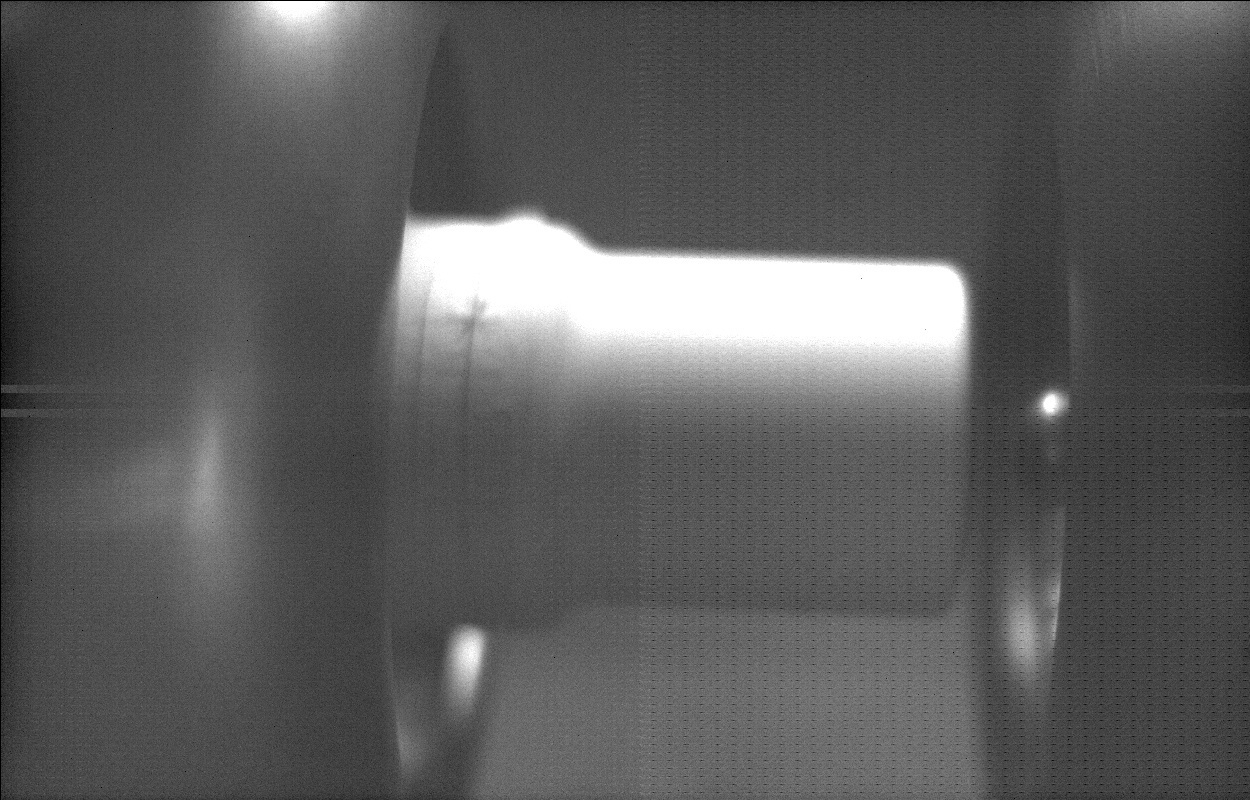

Supplement: S1 File — The relevant data can be obtained from the supporting information. The supporting information is mainly the image data analyzed in this paper. (ZIP) [file pone.0312253.s001.zip › supporting information/Nozzle/OPEN (53).jpg]

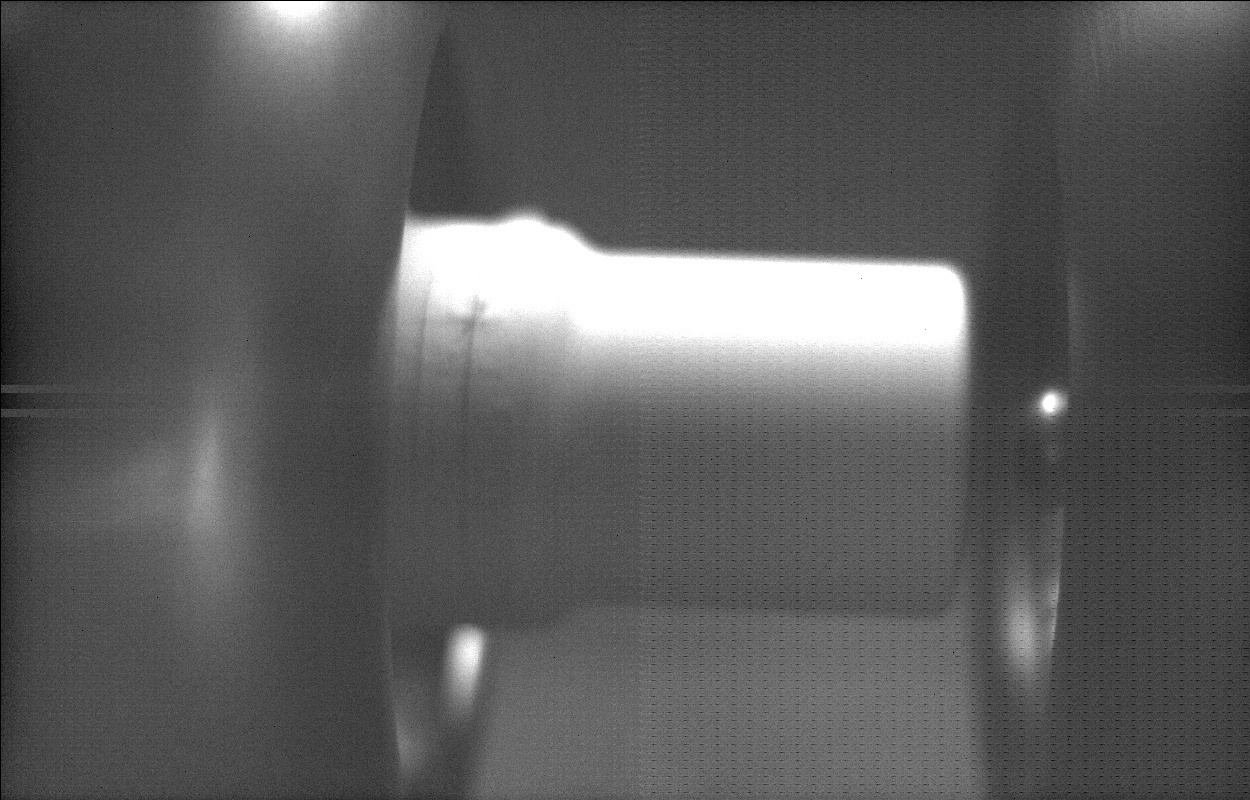

Supplement: S1 File — The relevant data can be obtained from the supporting information. The supporting information is mainly the image data analyzed in this paper. (ZIP) [file pone.0312253.s001.zip › supporting information/Nozzle/OPEN (54).jpg]

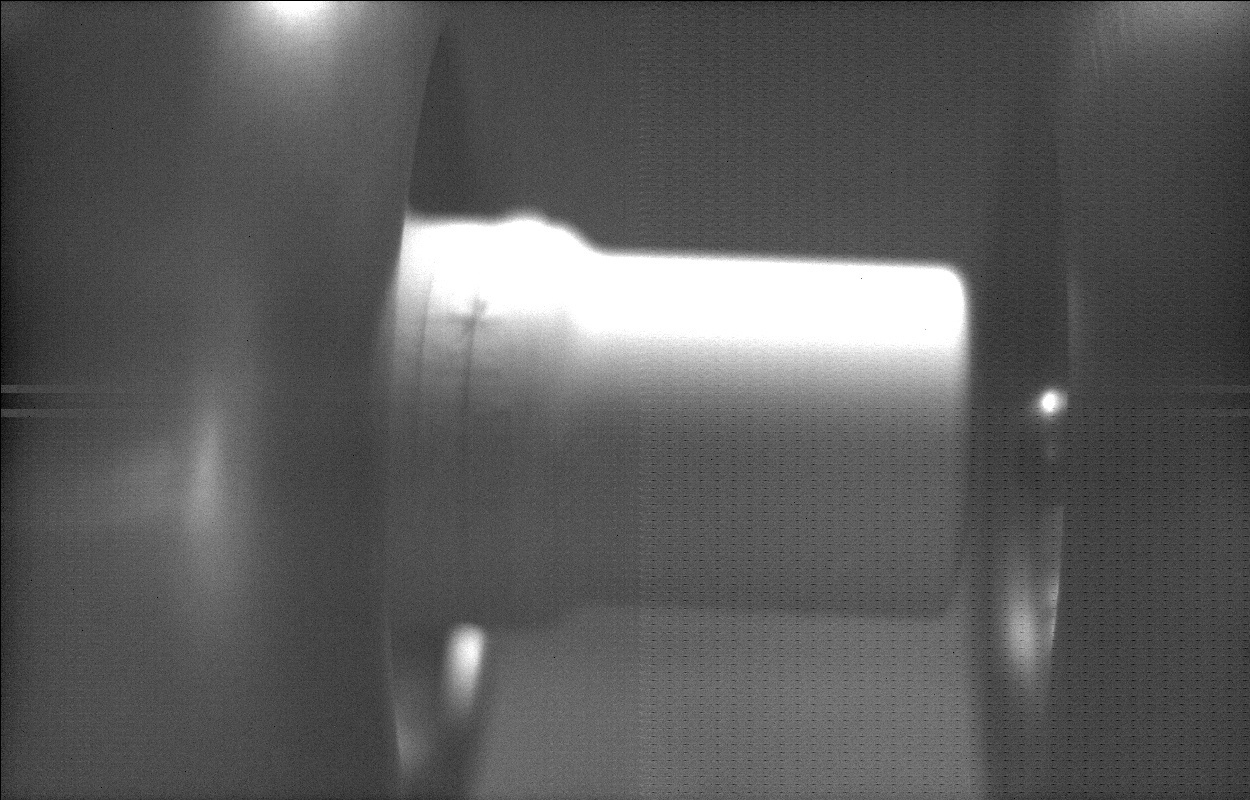

Supplement: S1 File — The relevant data can be obtained from the supporting information. The supporting information is mainly the image data analyzed in this paper. (ZIP) [file pone.0312253.s001.zip › supporting information/Nozzle/OPEN (55).jpg]

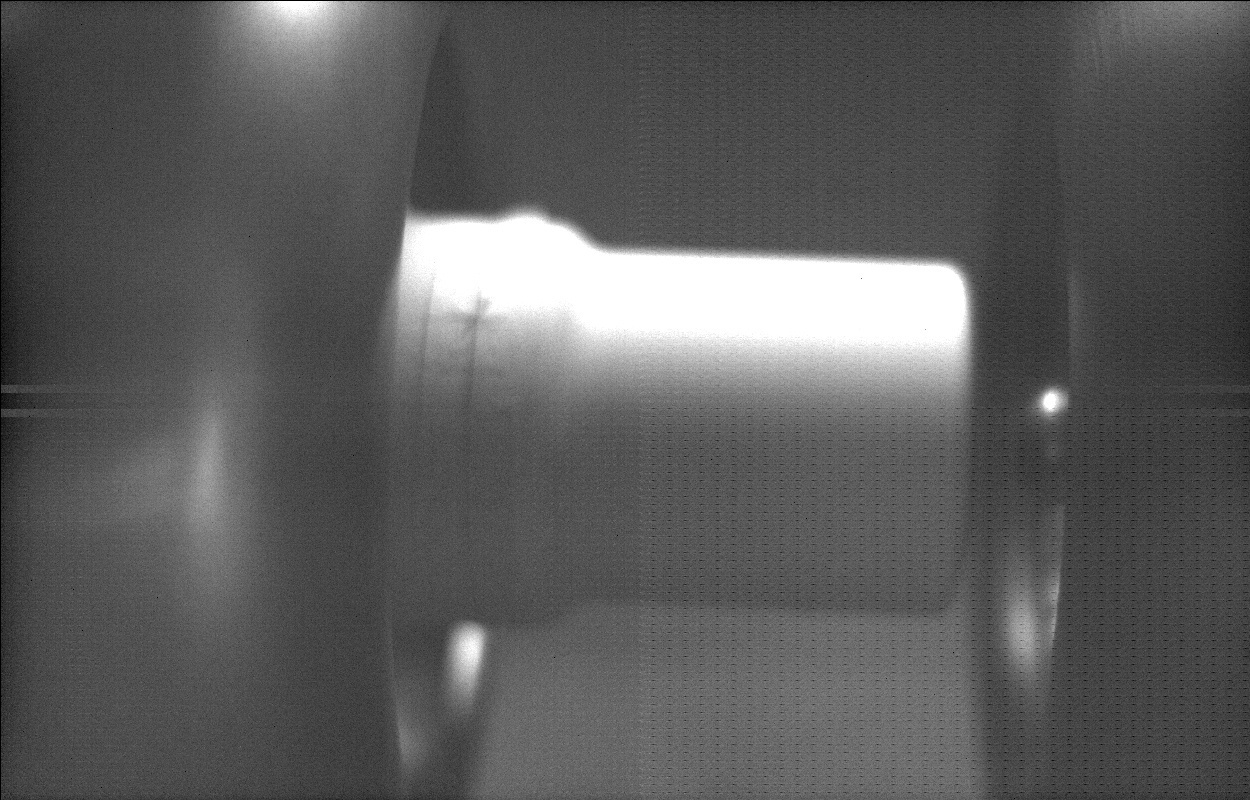

Supplement: S1 File — The relevant data can be obtained from the supporting information. The supporting information is mainly the image data analyzed in this paper. (ZIP) [file pone.0312253.s001.zip › supporting information/Nozzle/OPEN (56).jpg]

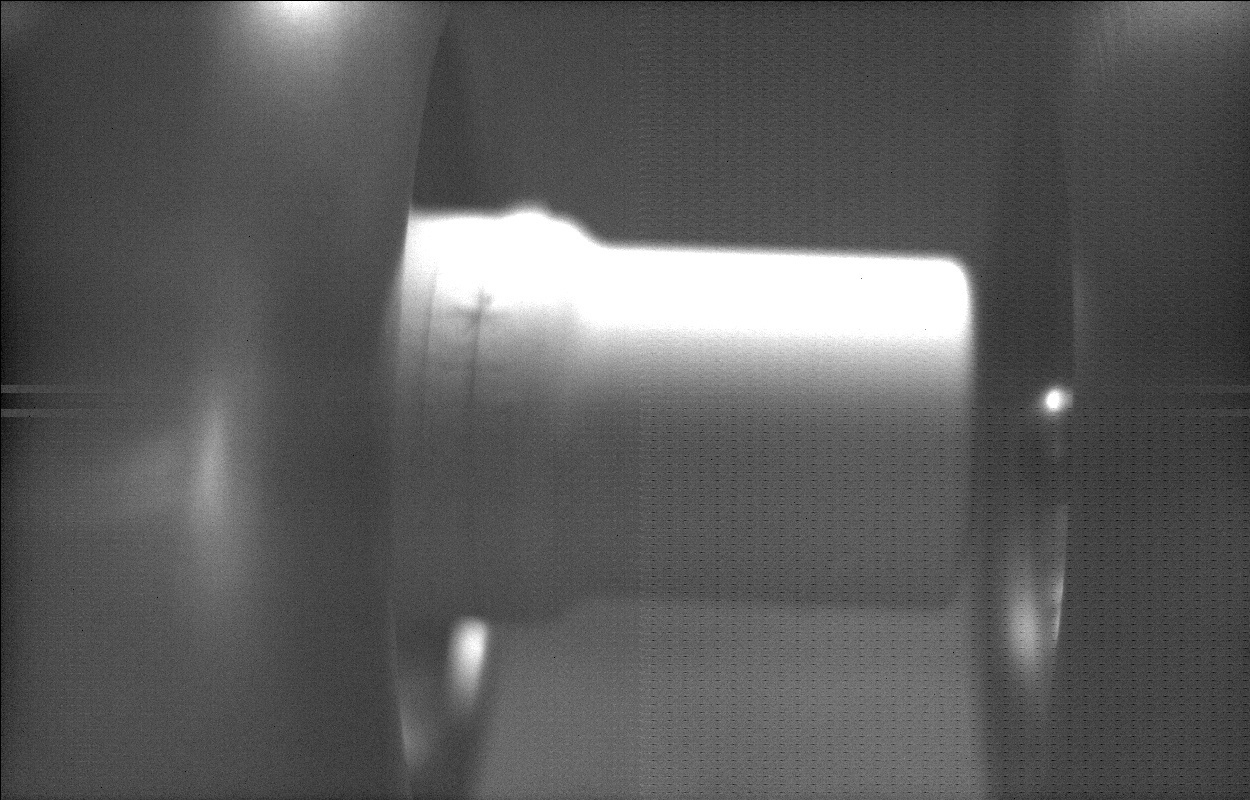

Supplement: S1 File — The relevant data can be obtained from the supporting information. The supporting information is mainly the image data analyzed in this paper. (ZIP) [file pone.0312253.s001.zip › supporting information/Nozzle/OPEN (57).jpg]

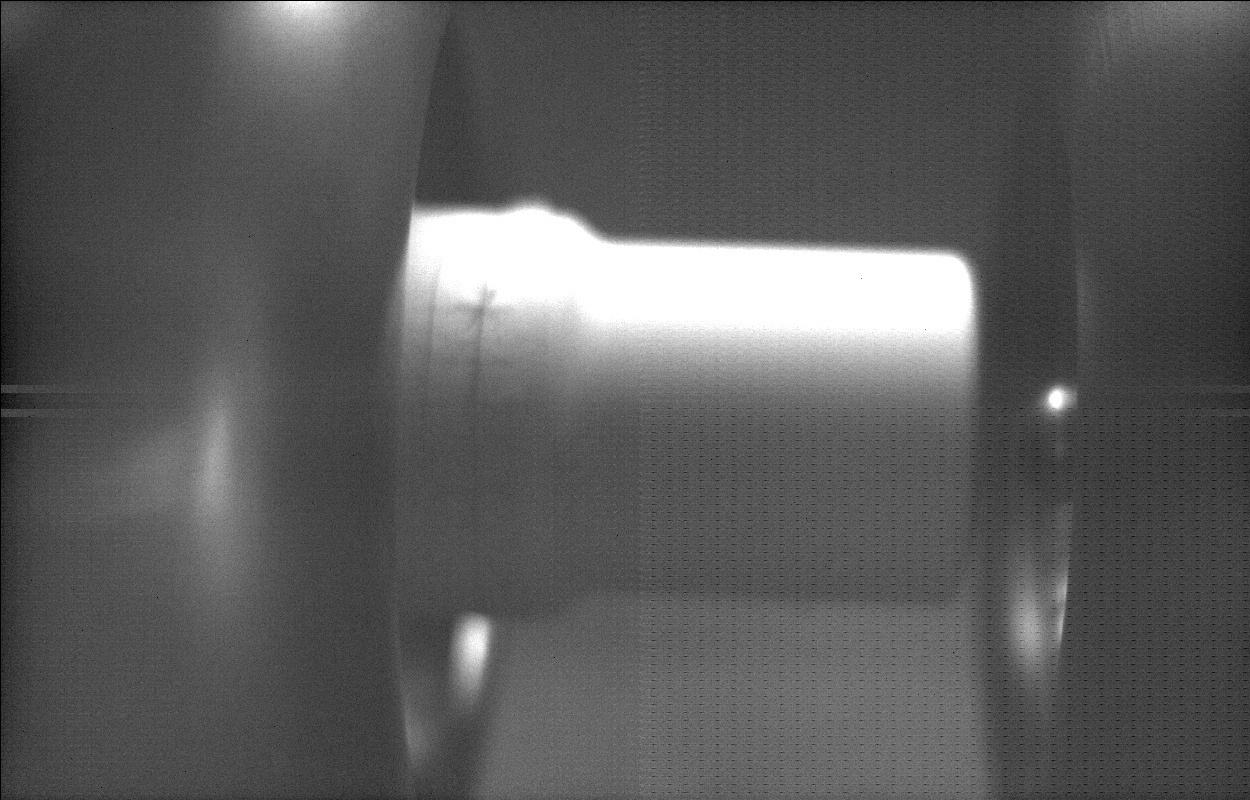

Supplement: S1 File — The relevant data can be obtained from the supporting information. The supporting information is mainly the image data analyzed in this paper. (ZIP) [file pone.0312253.s001.zip › supporting information/Nozzle/OPEN (58).jpg]

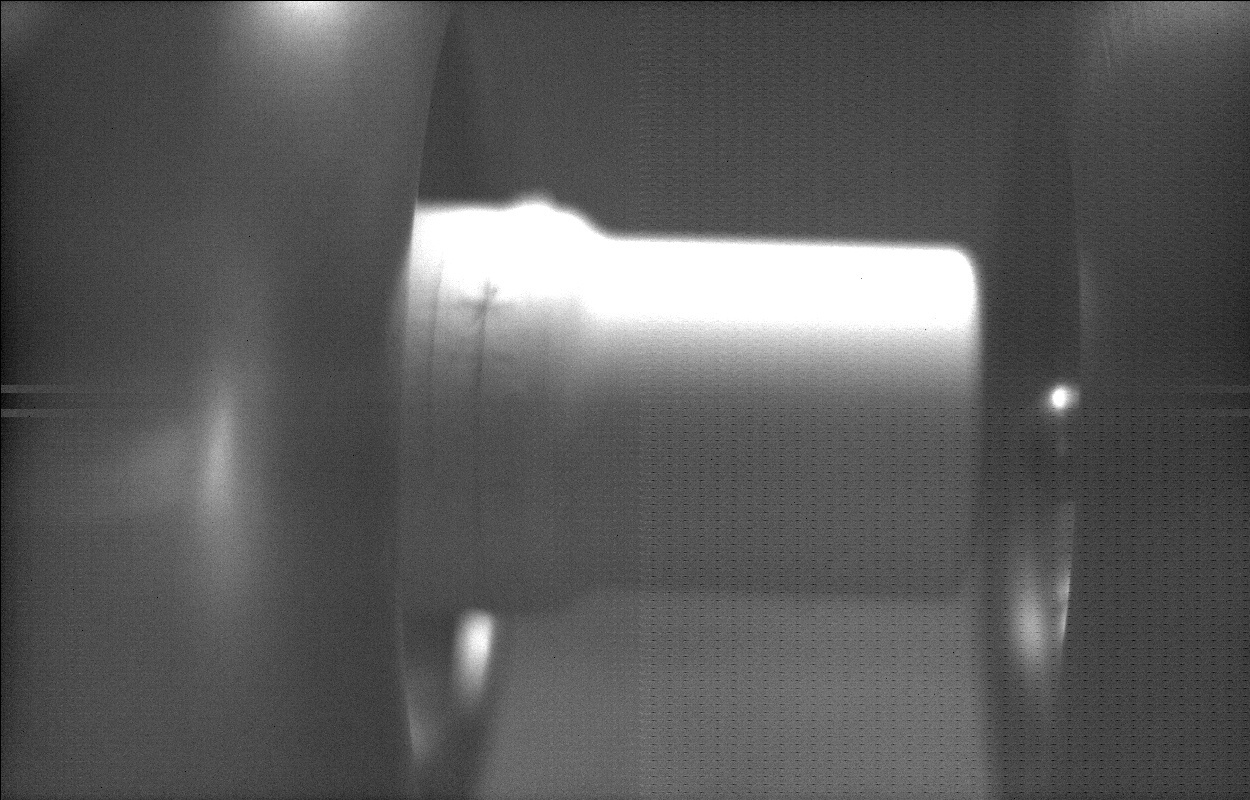

Supplement: S1 File — The relevant data can be obtained from the supporting information. The supporting information is mainly the image data analyzed in this paper. (ZIP) [file pone.0312253.s001.zip › supporting information/Nozzle/OPEN (59).jpg]

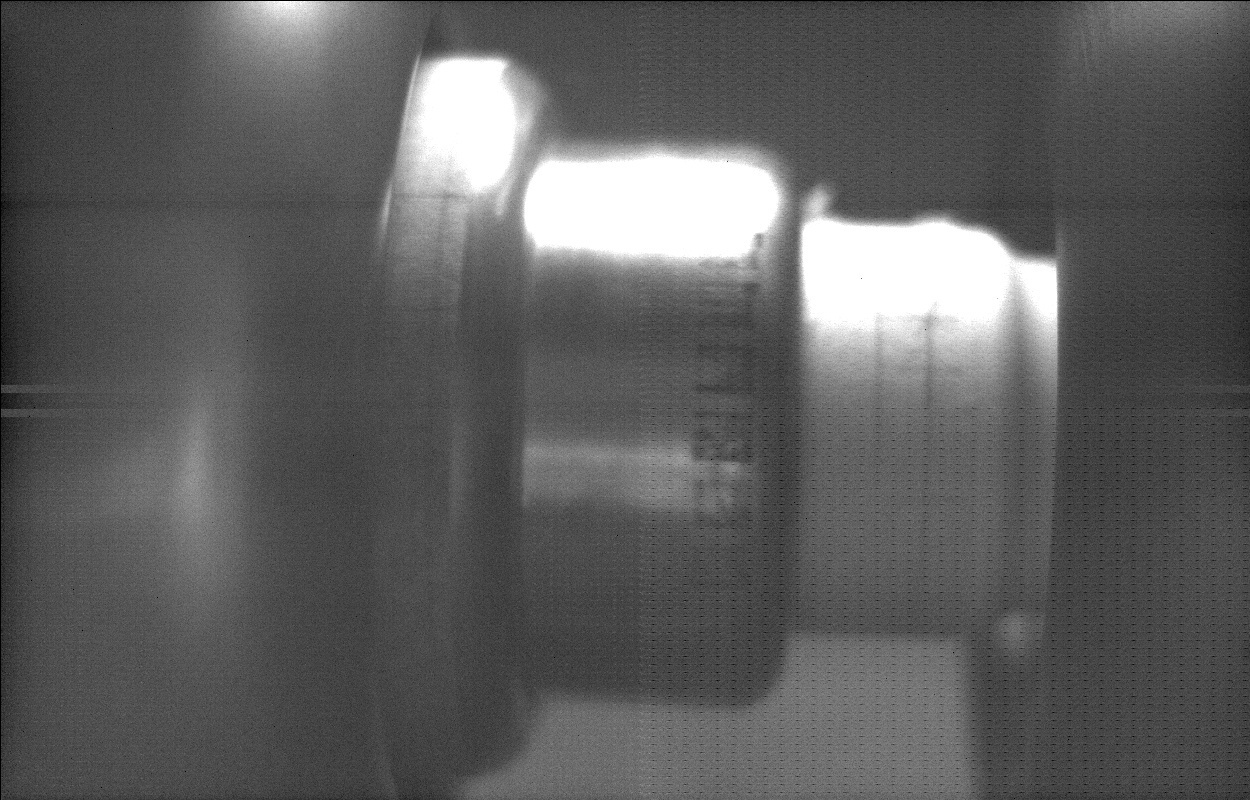

Supplement: S1 File — The relevant data can be obtained from the supporting information. The supporting information is mainly the image data analyzed in this paper. (ZIP) [file pone.0312253.s001.zip › supporting information/Nozzle/OPEN (6).jpg]

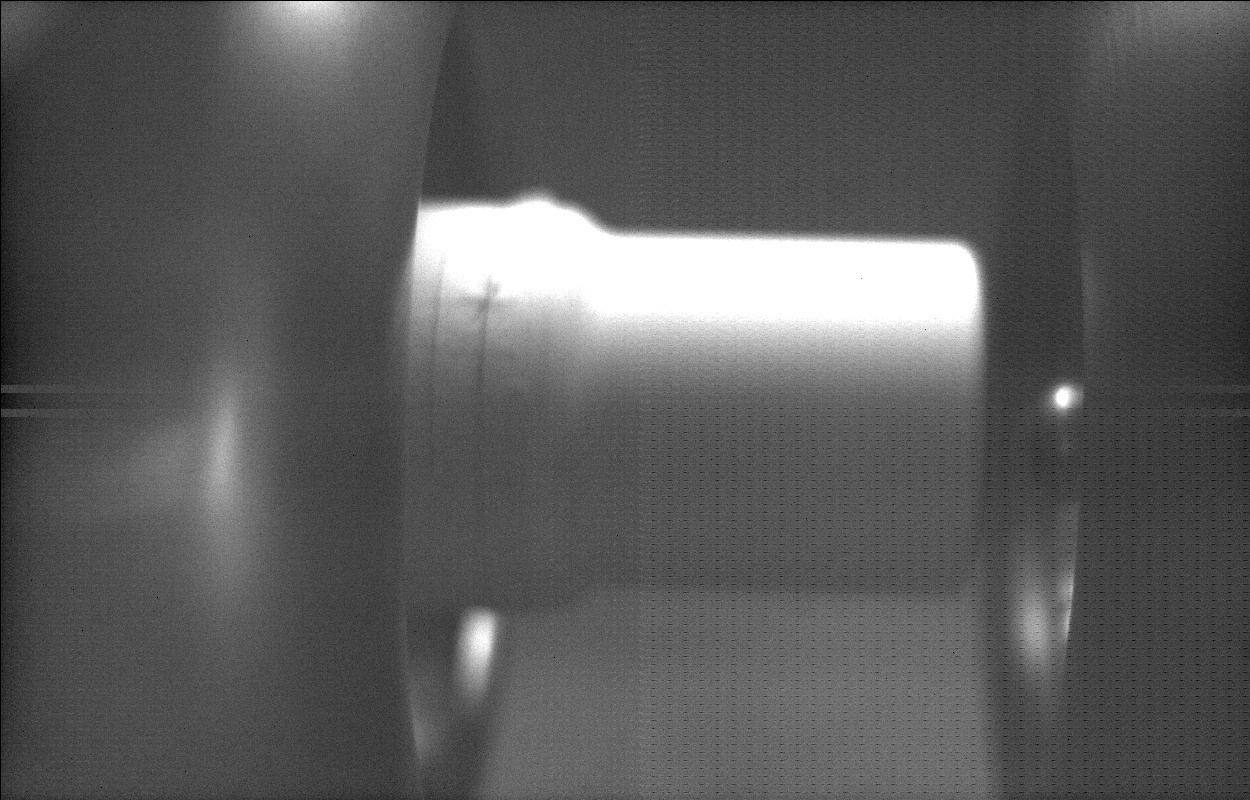

Supplement: S1 File — The relevant data can be obtained from the supporting information. The supporting information is mainly the image data analyzed in this paper. (ZIP) [file pone.0312253.s001.zip › supporting information/Nozzle/OPEN (60).jpg]

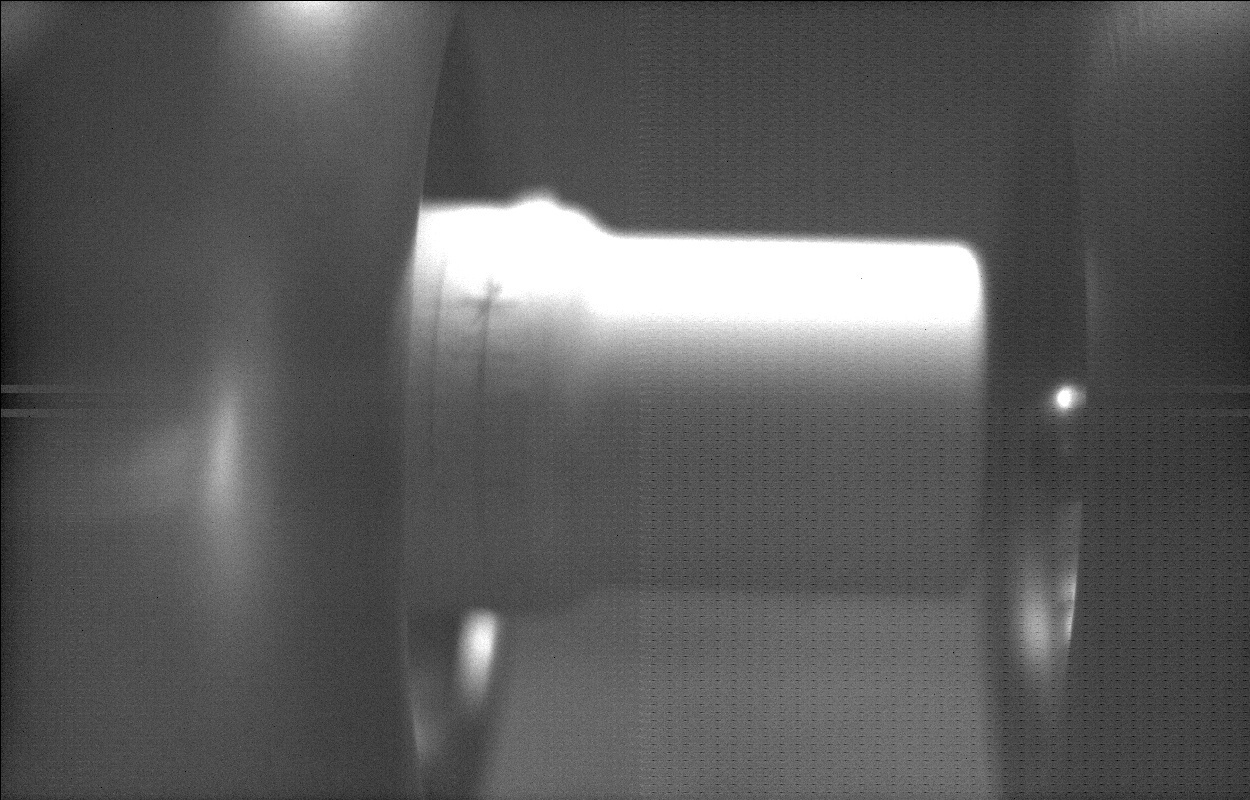

Supplement: S1 File — The relevant data can be obtained from the supporting information. The supporting information is mainly the image data analyzed in this paper. (ZIP) [file pone.0312253.s001.zip › supporting information/Nozzle/OPEN (61).jpg]

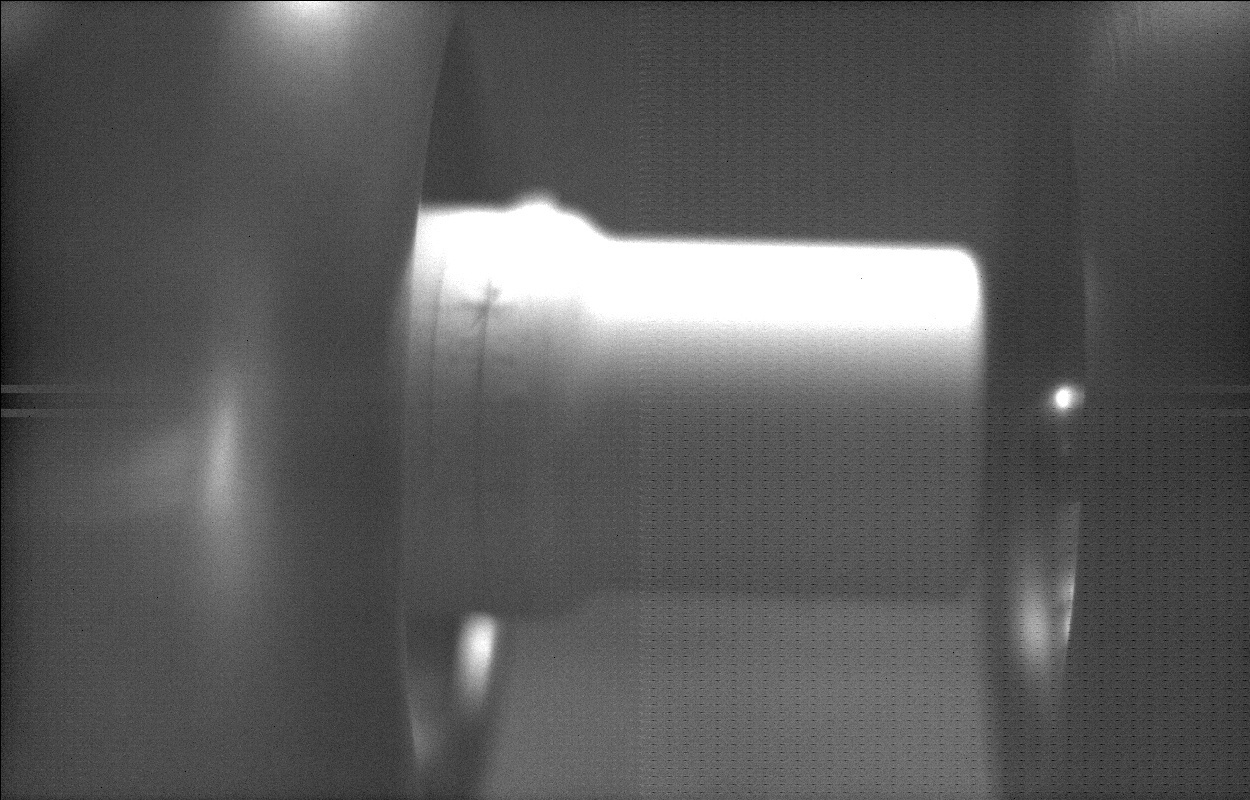

Supplement: S1 File — The relevant data can be obtained from the supporting information. The supporting information is mainly the image data analyzed in this paper. (ZIP) [file pone.0312253.s001.zip › supporting information/Nozzle/OPEN (62).jpg]

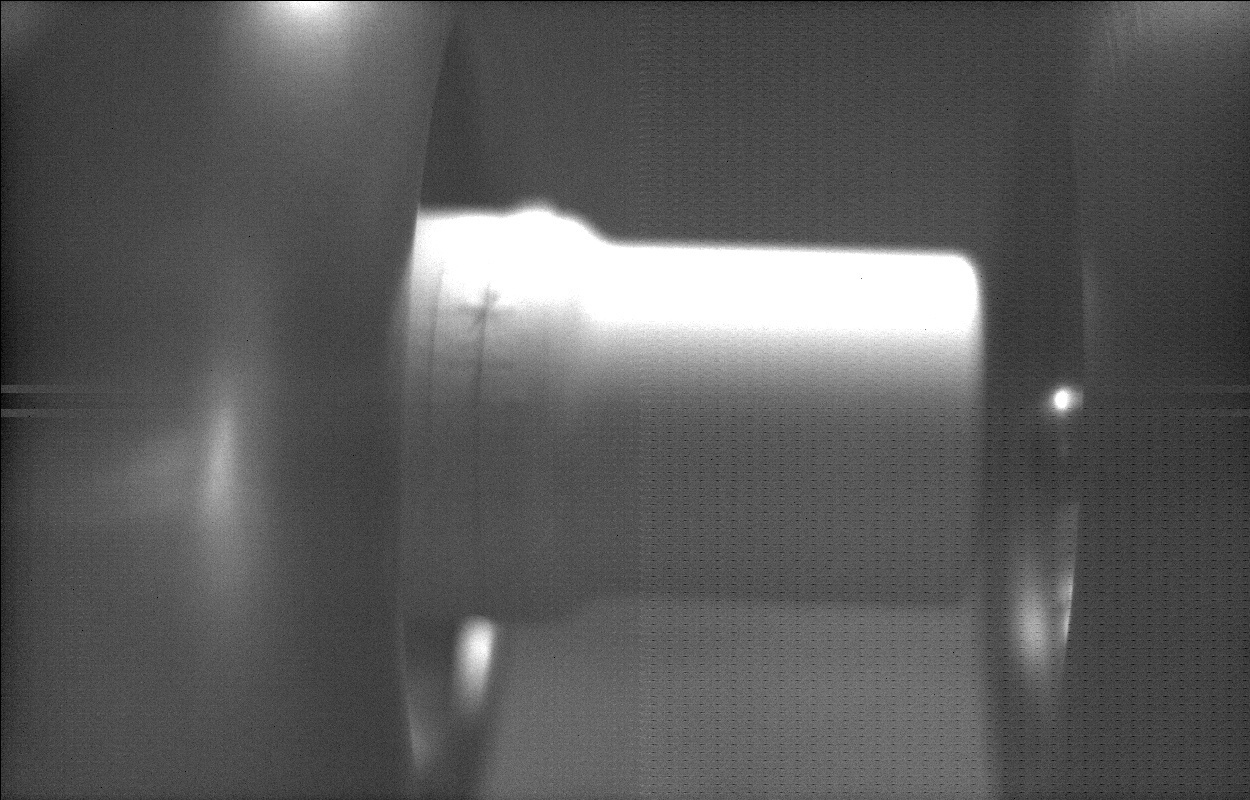

Supplement: S1 File — The relevant data can be obtained from the supporting information. The supporting information is mainly the image data analyzed in this paper. (ZIP) [file pone.0312253.s001.zip › supporting information/Nozzle/OPEN (63).jpg]

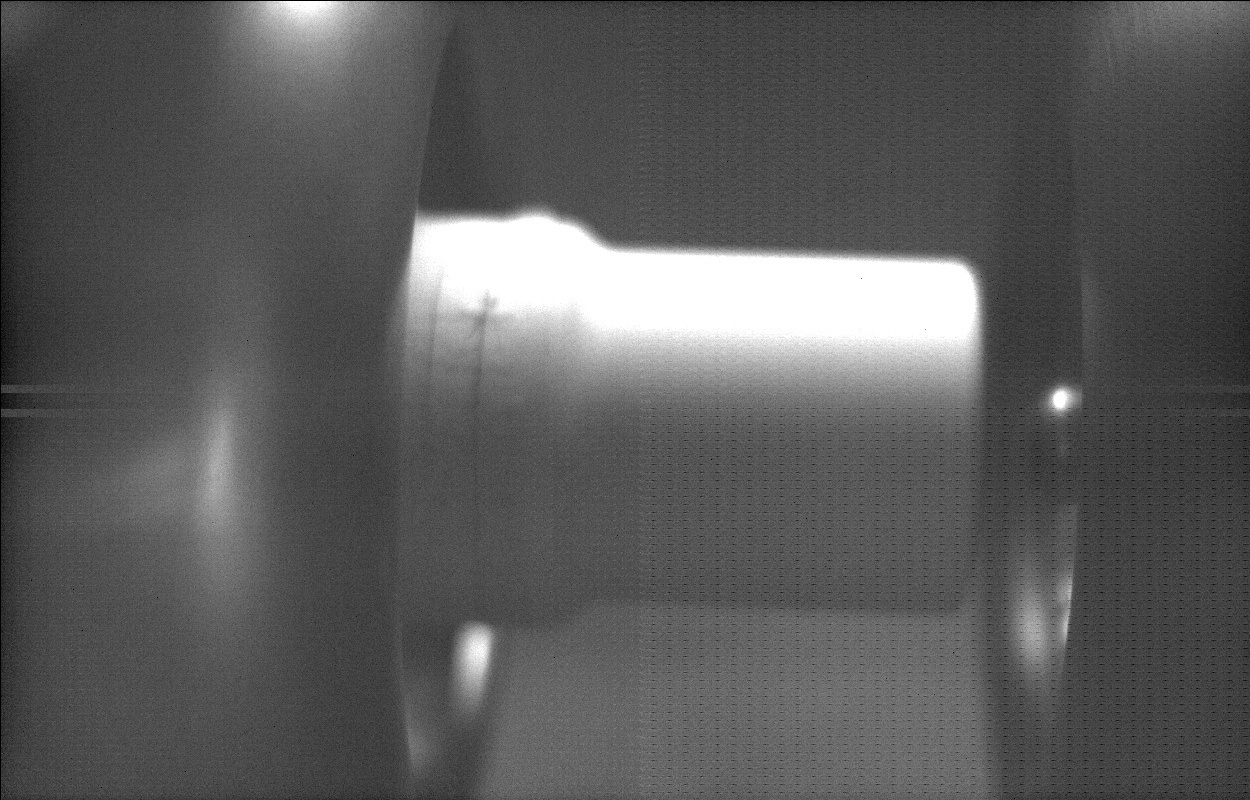

Supplement: S1 File — The relevant data can be obtained from the supporting information. The supporting information is mainly the image data analyzed in this paper. (ZIP) [file pone.0312253.s001.zip › supporting information/Nozzle/OPEN (64).jpg]

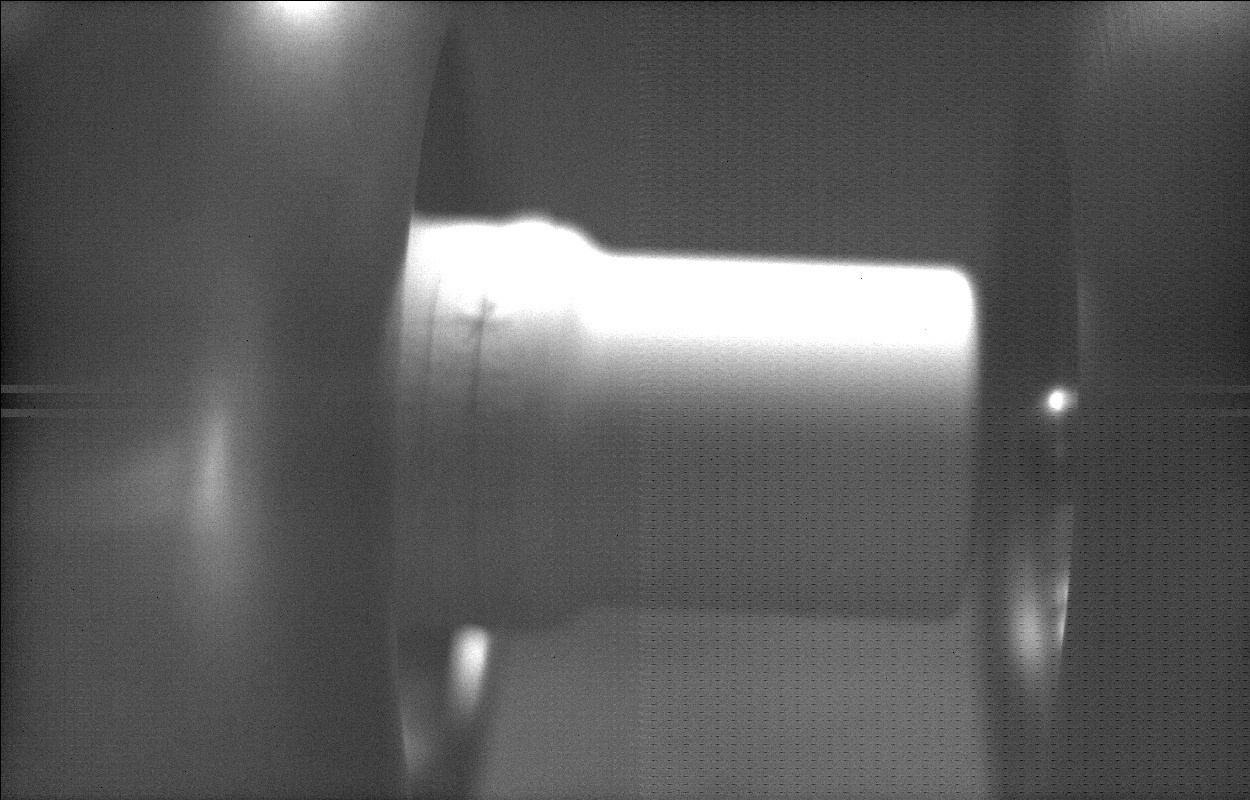

Supplement: S1 File — The relevant data can be obtained from the supporting information. The supporting information is mainly the image data analyzed in this paper. (ZIP) [file pone.0312253.s001.zip › supporting information/Nozzle/OPEN (65).jpg]

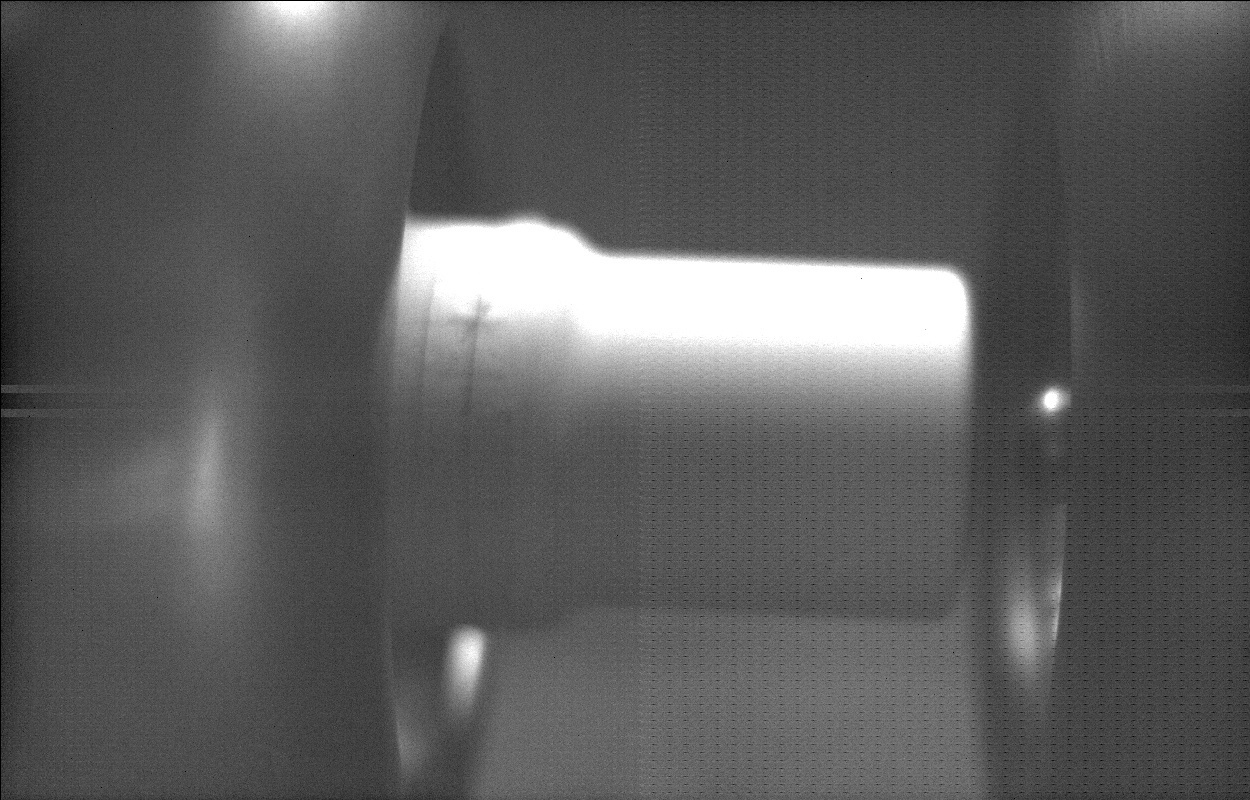

Supplement: S1 File — The relevant data can be obtained from the supporting information. The supporting information is mainly the image data analyzed in this paper. (ZIP) [file pone.0312253.s001.zip › supporting information/Nozzle/OPEN (66).jpg]

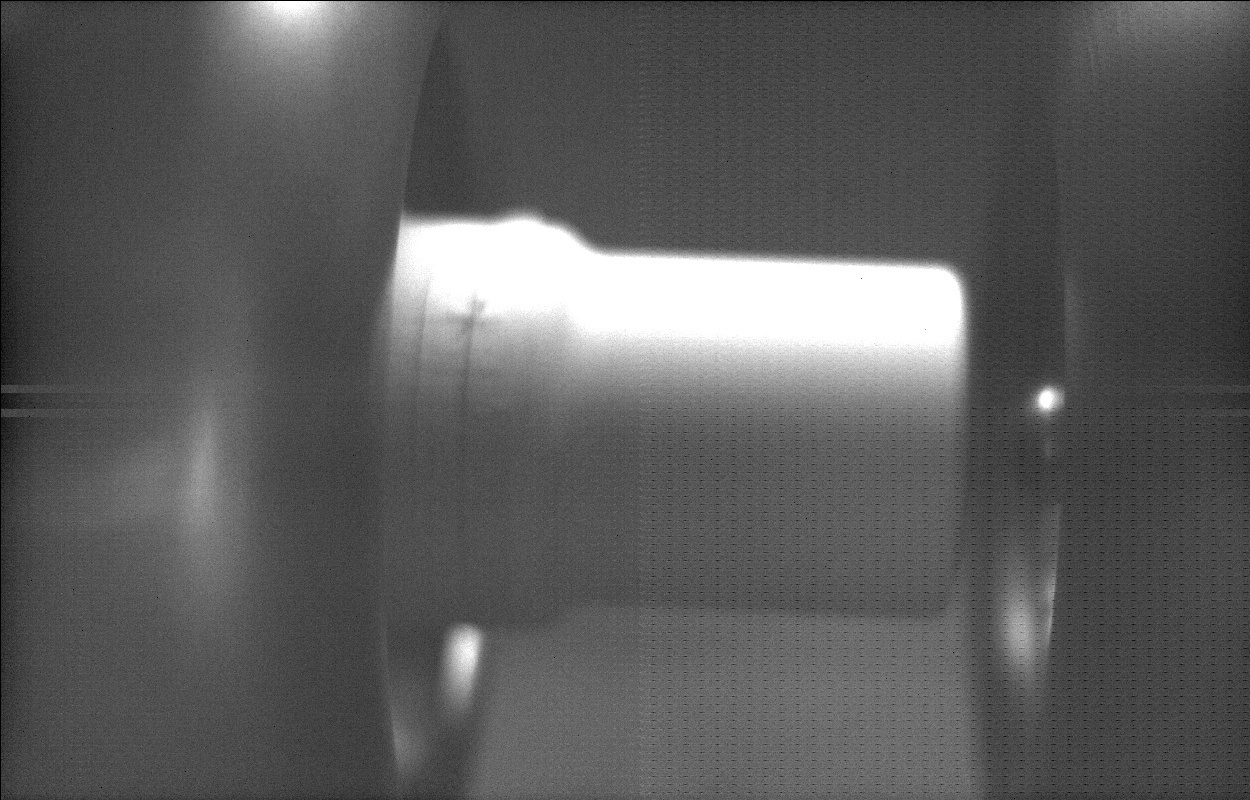

Supplement: S1 File — The relevant data can be obtained from the supporting information. The supporting information is mainly the image data analyzed in this paper. (ZIP) [file pone.0312253.s001.zip › supporting information/Nozzle/OPEN (67).jpg]

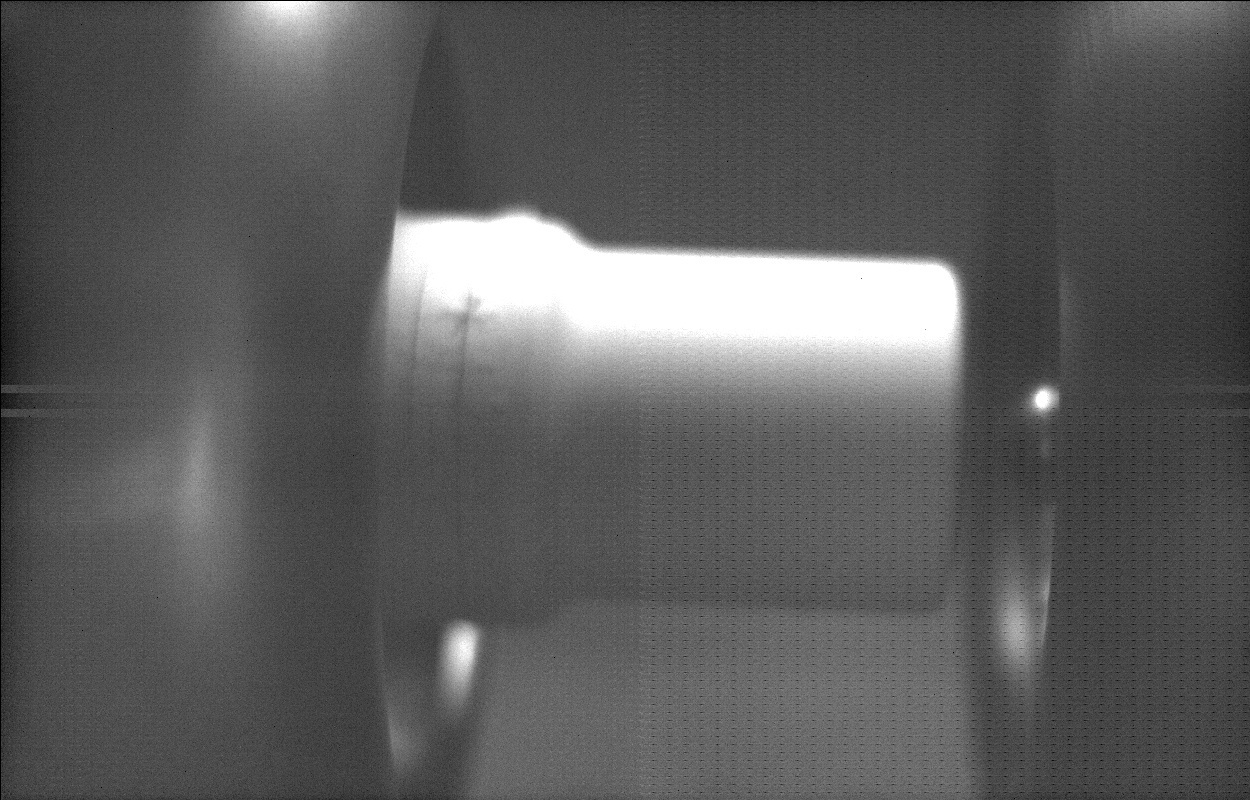

Supplement: S1 File — The relevant data can be obtained from the supporting information. The supporting information is mainly the image data analyzed in this paper. (ZIP) [file pone.0312253.s001.zip › supporting information/Nozzle/OPEN (68).jpg]

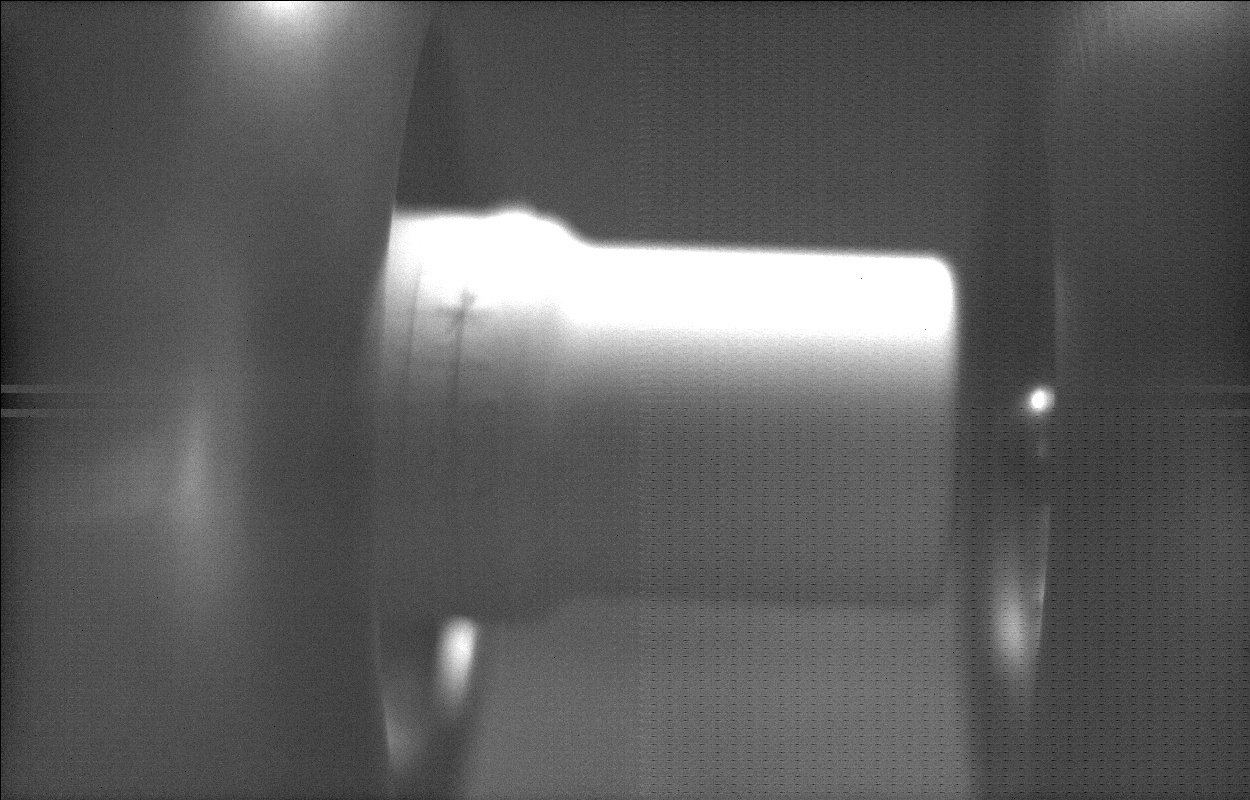

Supplement: S1 File — The relevant data can be obtained from the supporting information. The supporting information is mainly the image data analyzed in this paper. (ZIP) [file pone.0312253.s001.zip › supporting information/Nozzle/OPEN (69).jpg]

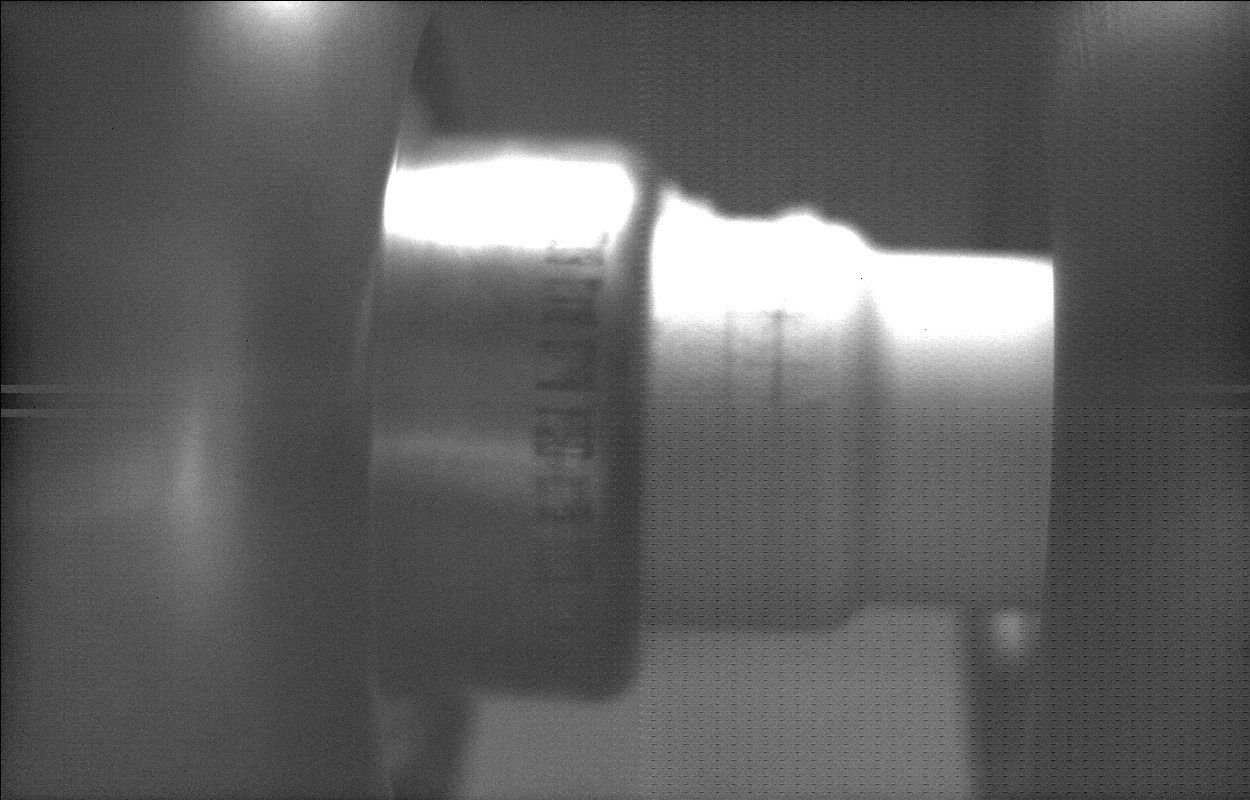

Supplement: S1 File — The relevant data can be obtained from the supporting information. The supporting information is mainly the image data analyzed in this paper. (ZIP) [file pone.0312253.s001.zip › supporting information/Nozzle/OPEN (7).jpg]

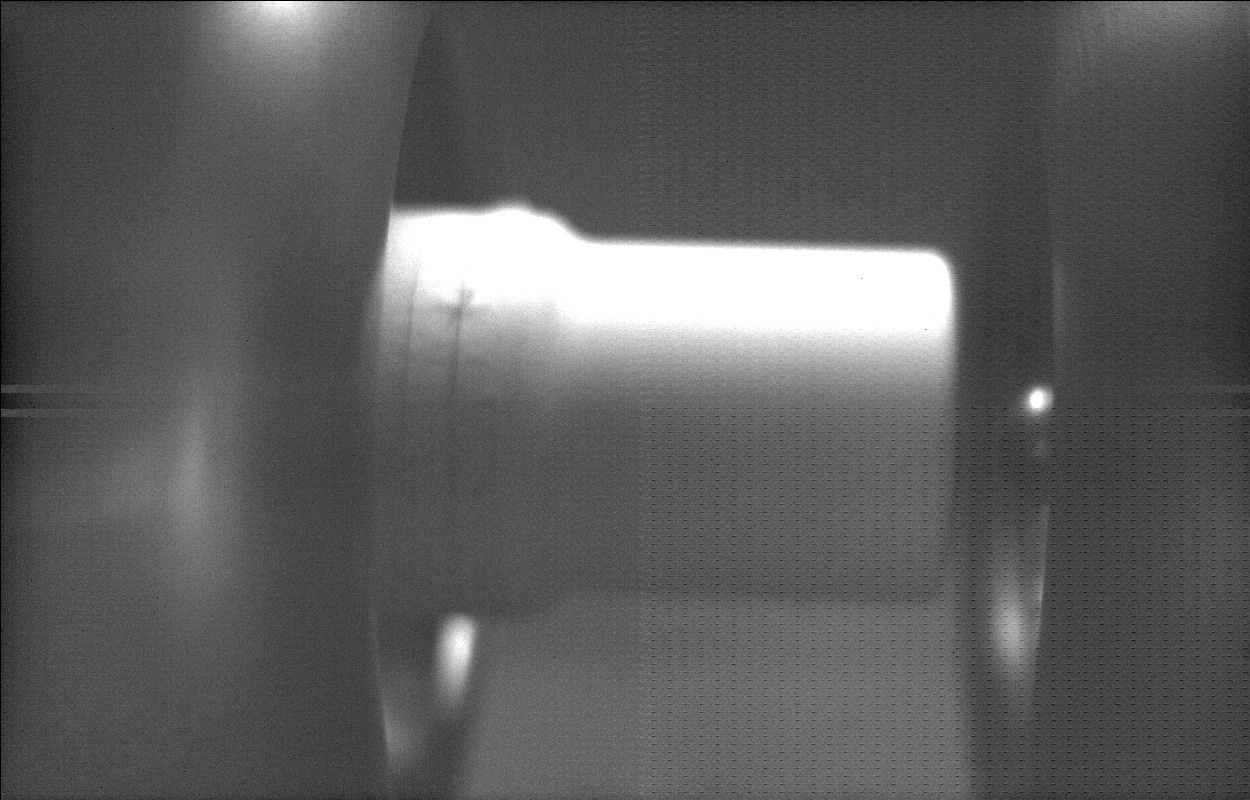

Supplement: S1 File — The relevant data can be obtained from the supporting information. The supporting information is mainly the image data analyzed in this paper. (ZIP) [file pone.0312253.s001.zip › supporting information/Nozzle/OPEN (70).jpg]

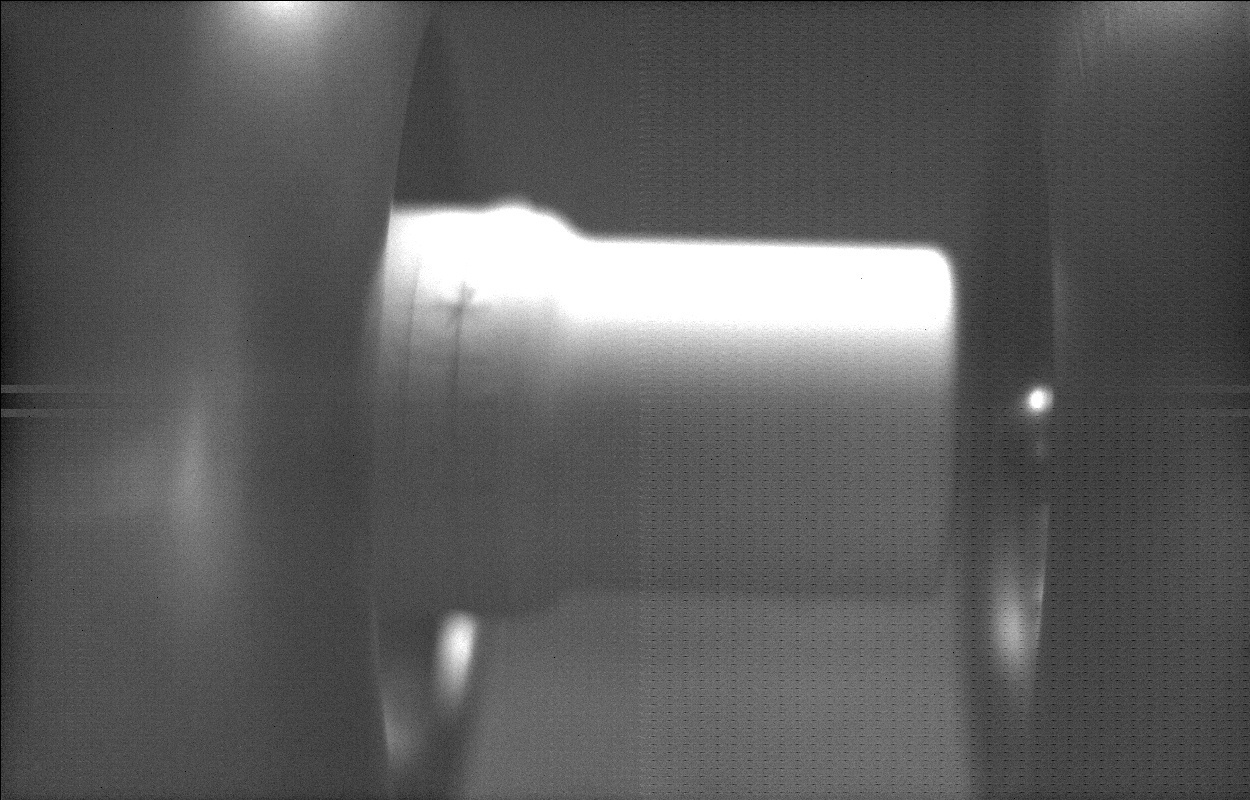

Supplement: S1 File — The relevant data can be obtained from the supporting information. The supporting information is mainly the image data analyzed in this paper. (ZIP) [file pone.0312253.s001.zip › supporting information/Nozzle/OPEN (71).jpg]

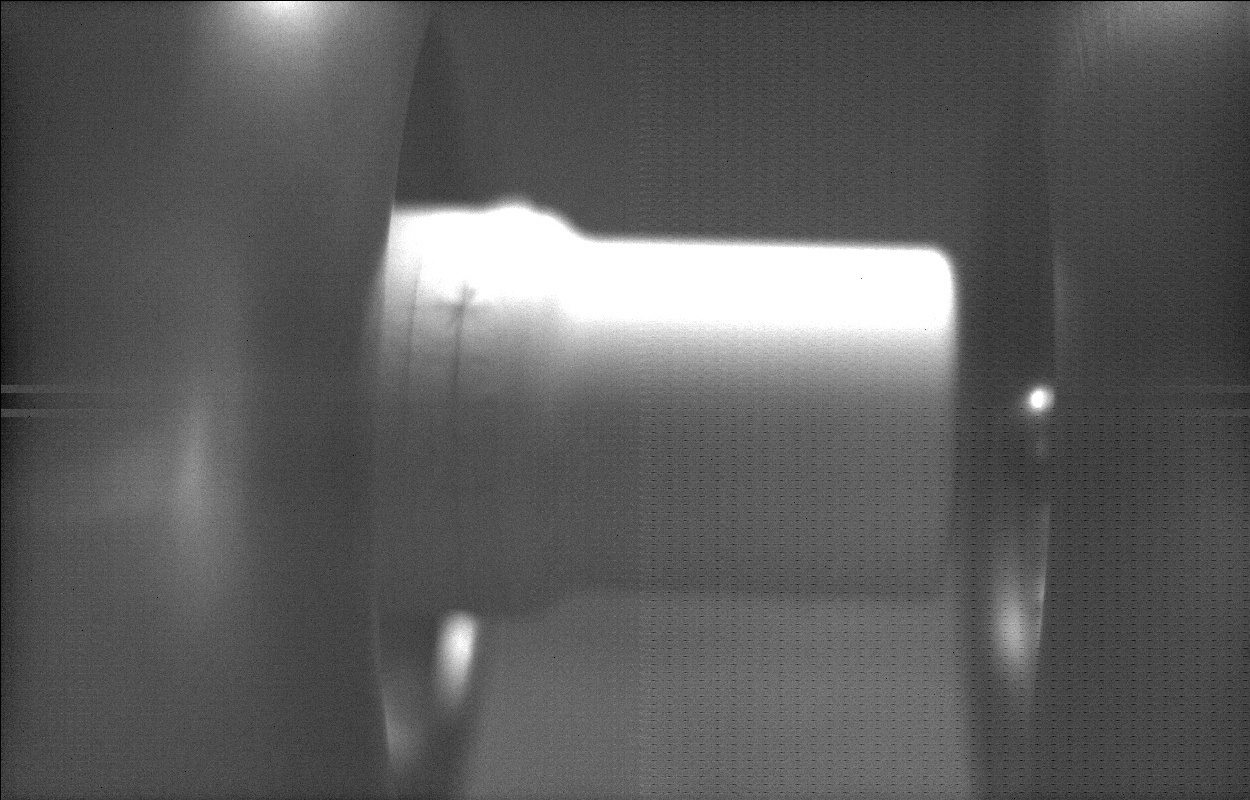

Supplement: S1 File — The relevant data can be obtained from the supporting information. The supporting information is mainly the image data analyzed in this paper. (ZIP) [file pone.0312253.s001.zip › supporting information/Nozzle/OPEN (72).jpg]

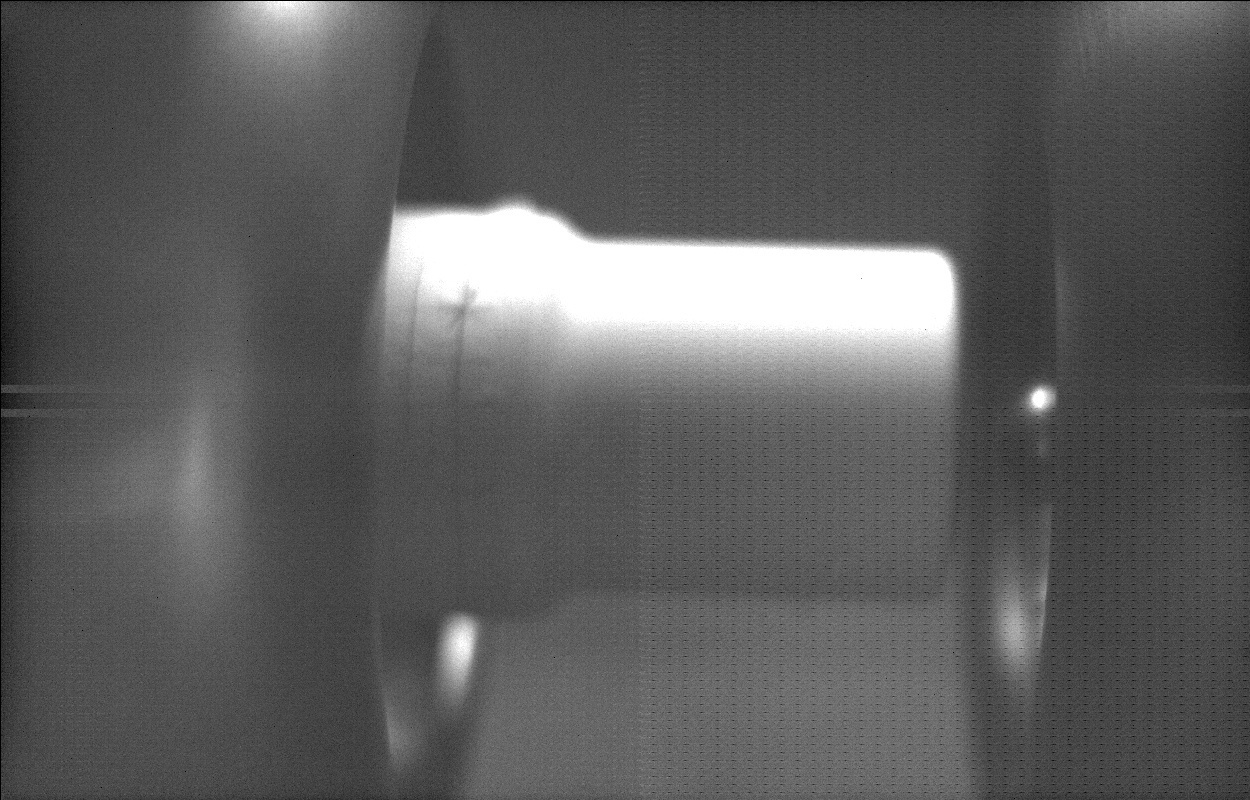

Supplement: S1 File — The relevant data can be obtained from the supporting information. The supporting information is mainly the image data analyzed in this paper. (ZIP) [file pone.0312253.s001.zip › supporting information/Nozzle/OPEN (73).jpg]

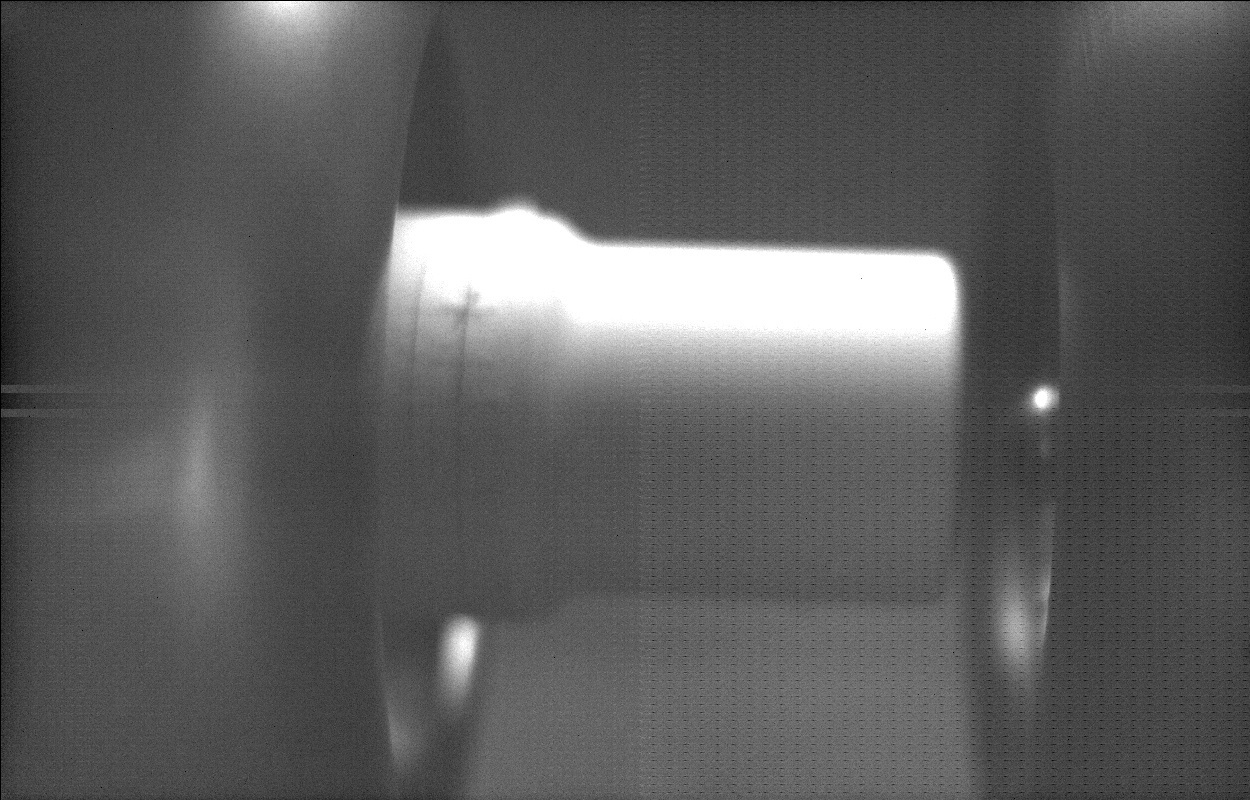

Supplement: S1 File — The relevant data can be obtained from the supporting information. The supporting information is mainly the image data analyzed in this paper. (ZIP) [file pone.0312253.s001.zip › supporting information/Nozzle/OPEN (74).jpg]

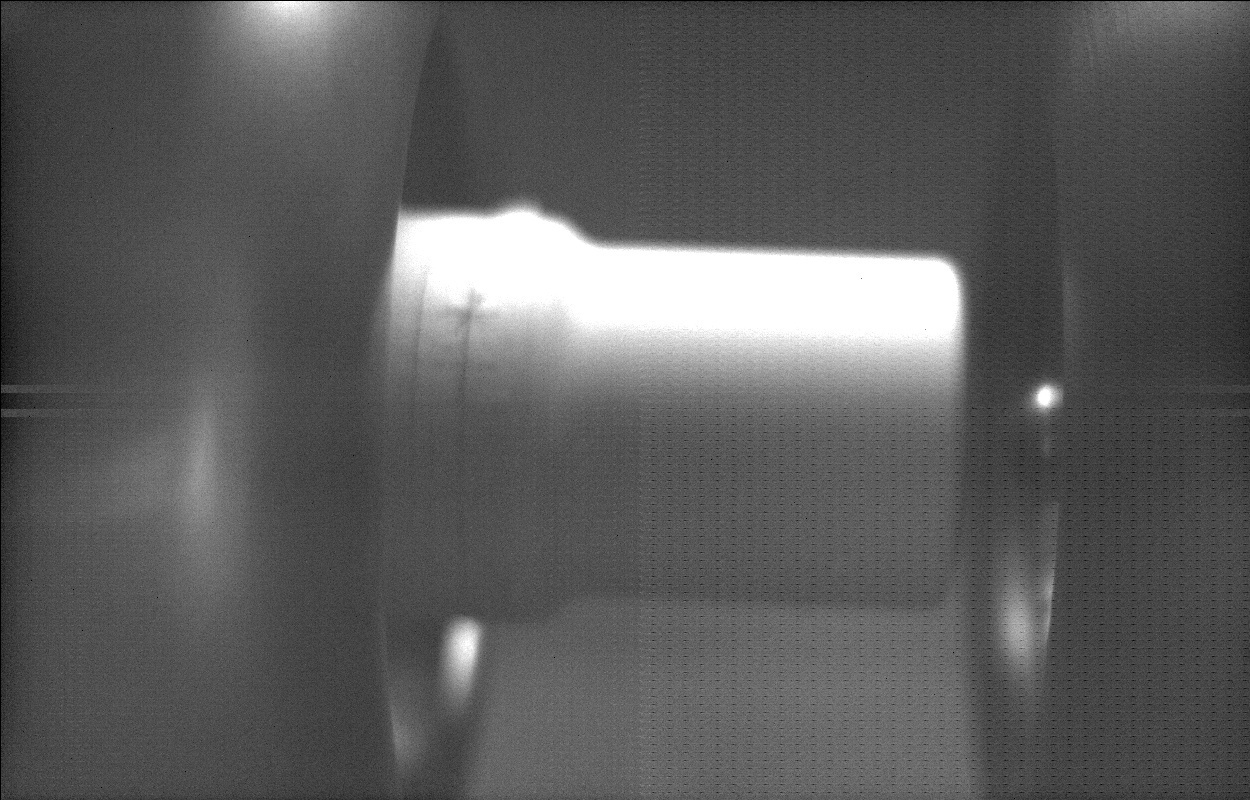

Supplement: S1 File — The relevant data can be obtained from the supporting information. The supporting information is mainly the image data analyzed in this paper. (ZIP) [file pone.0312253.s001.zip › supporting information/Nozzle/OPEN (75).jpg]

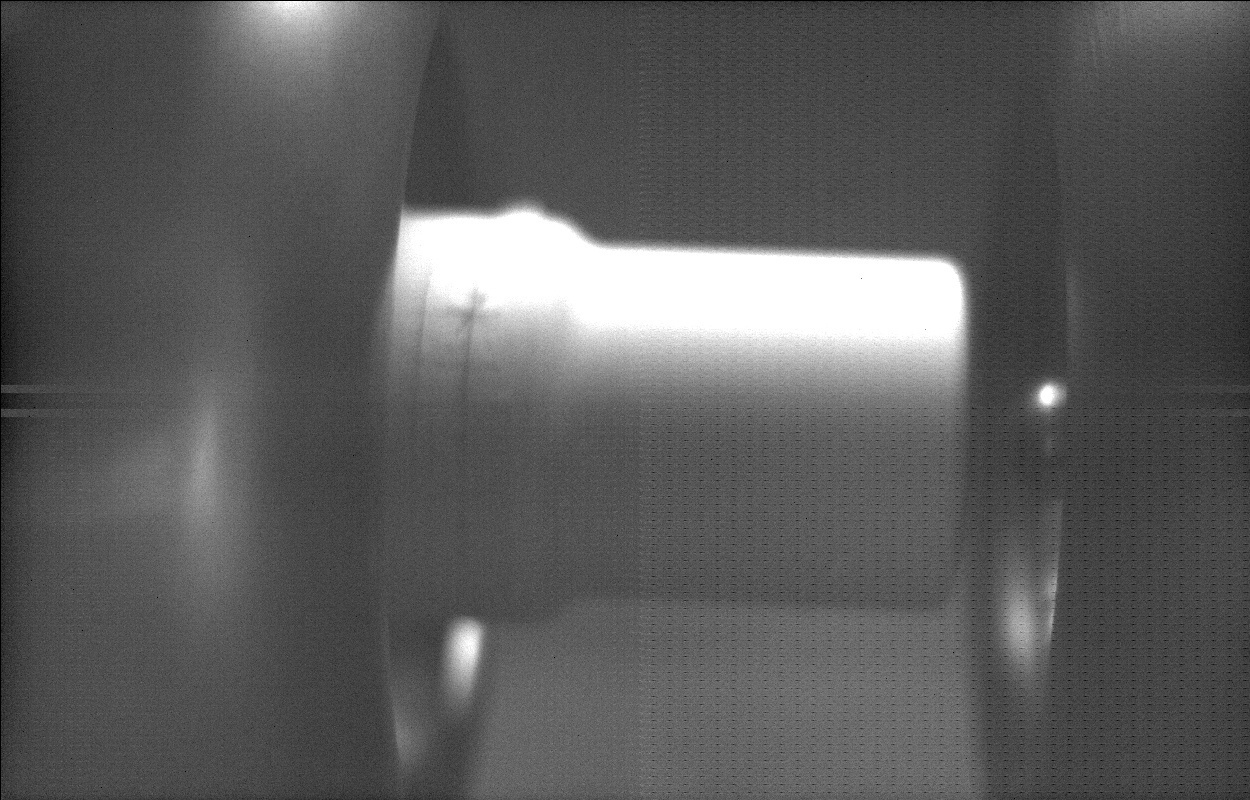

Supplement: S1 File — The relevant data can be obtained from the supporting information. The supporting information is mainly the image data analyzed in this paper. (ZIP) [file pone.0312253.s001.zip › supporting information/Nozzle/OPEN (76).jpg]

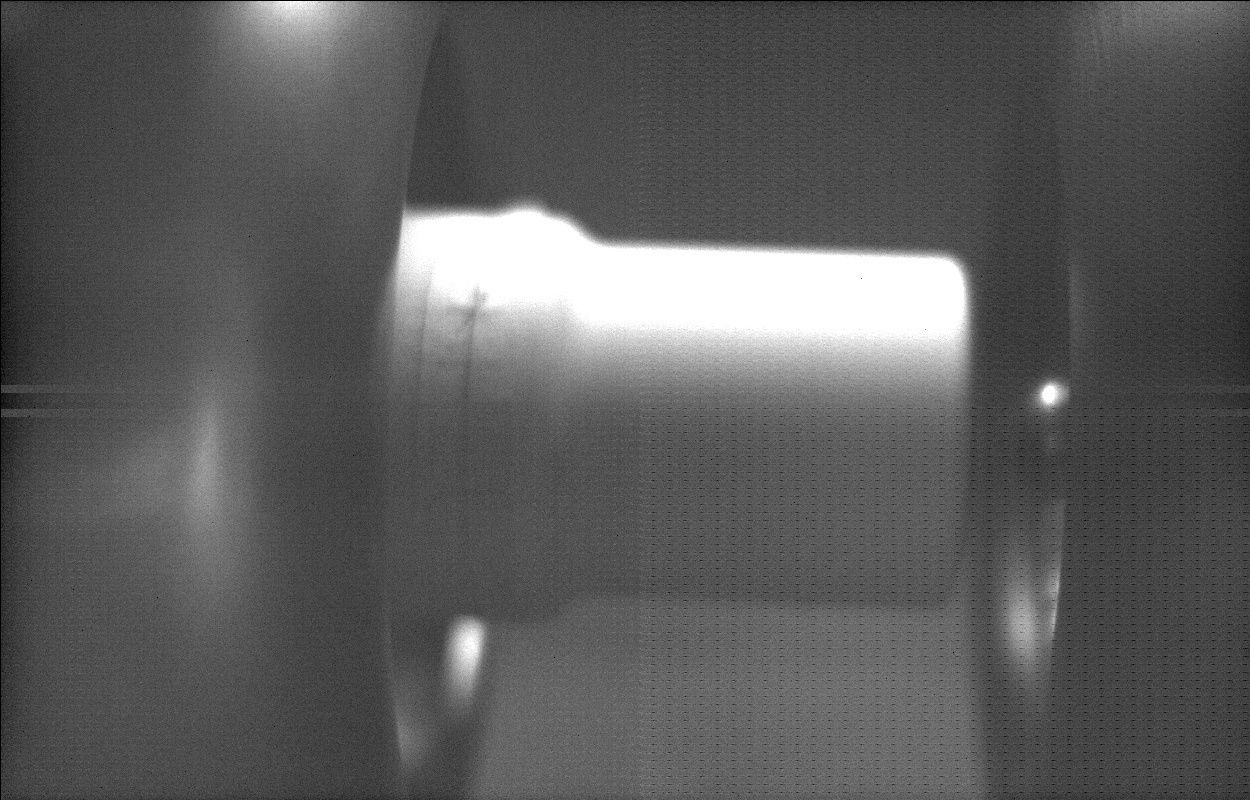

Supplement: S1 File — The relevant data can be obtained from the supporting information. The supporting information is mainly the image data analyzed in this paper. (ZIP) [file pone.0312253.s001.zip › supporting information/Nozzle/OPEN (77).jpg]

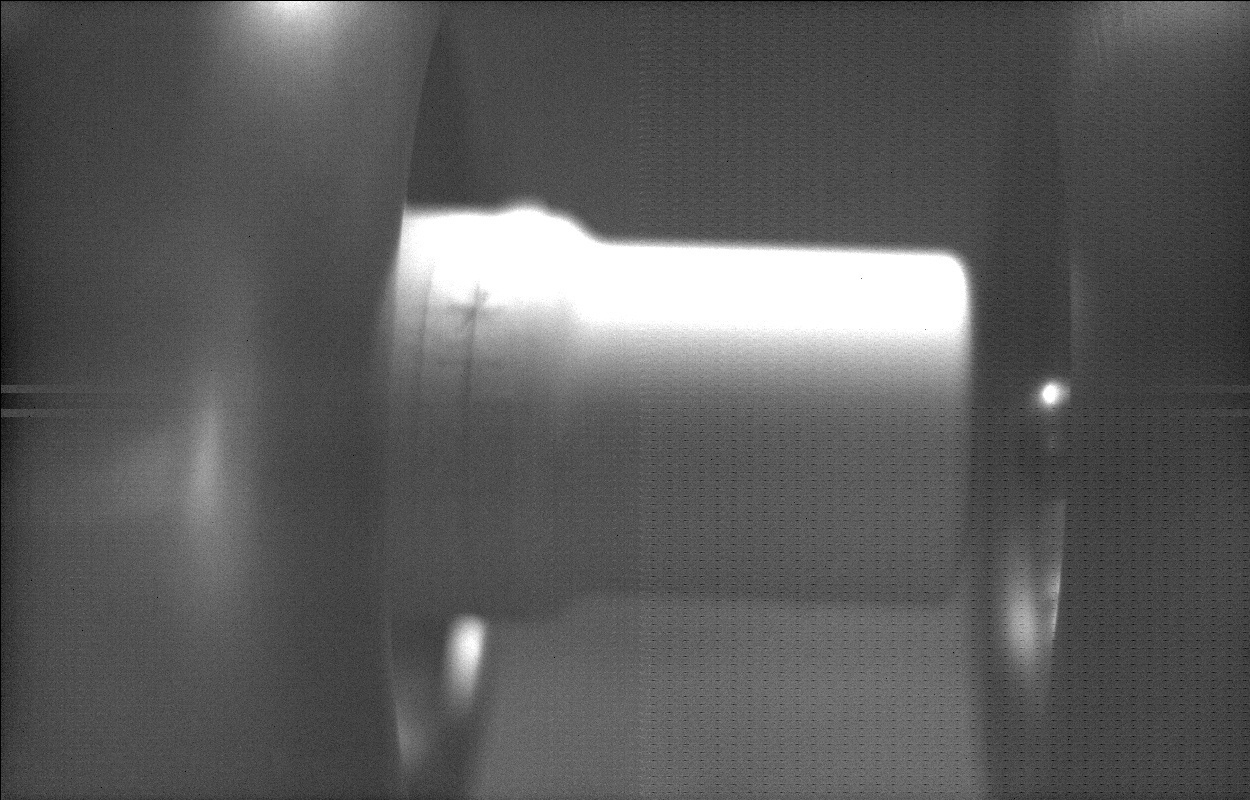

Supplement: S1 File — The relevant data can be obtained from the supporting information. The supporting information is mainly the image data analyzed in this paper. (ZIP) [file pone.0312253.s001.zip › supporting information/Nozzle/OPEN (78).jpg]

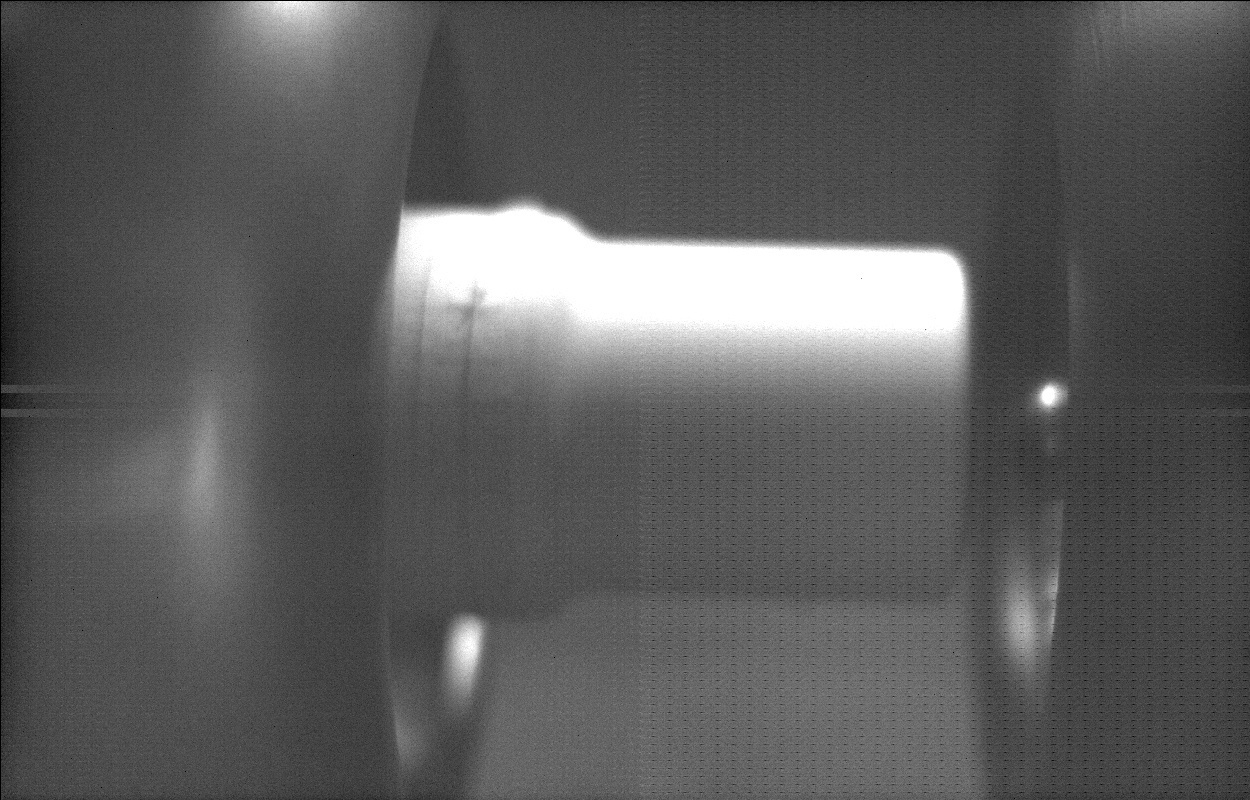

Supplement: S1 File — The relevant data can be obtained from the supporting information. The supporting information is mainly the image data analyzed in this paper. (ZIP) [file pone.0312253.s001.zip › supporting information/Nozzle/OPEN (79).jpg]

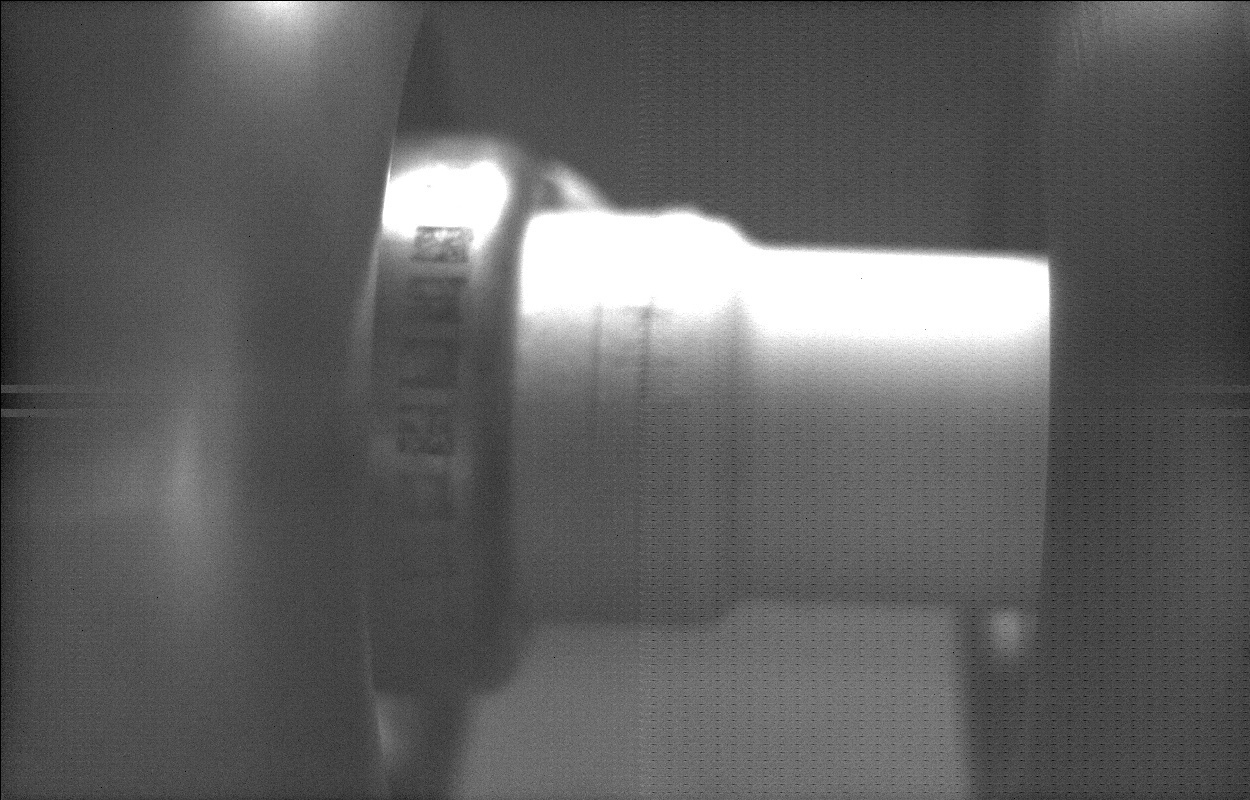

Supplement: S1 File — The relevant data can be obtained from the supporting information. The supporting information is mainly the image data analyzed in this paper. (ZIP) [file pone.0312253.s001.zip › supporting information/Nozzle/OPEN (8).jpg]

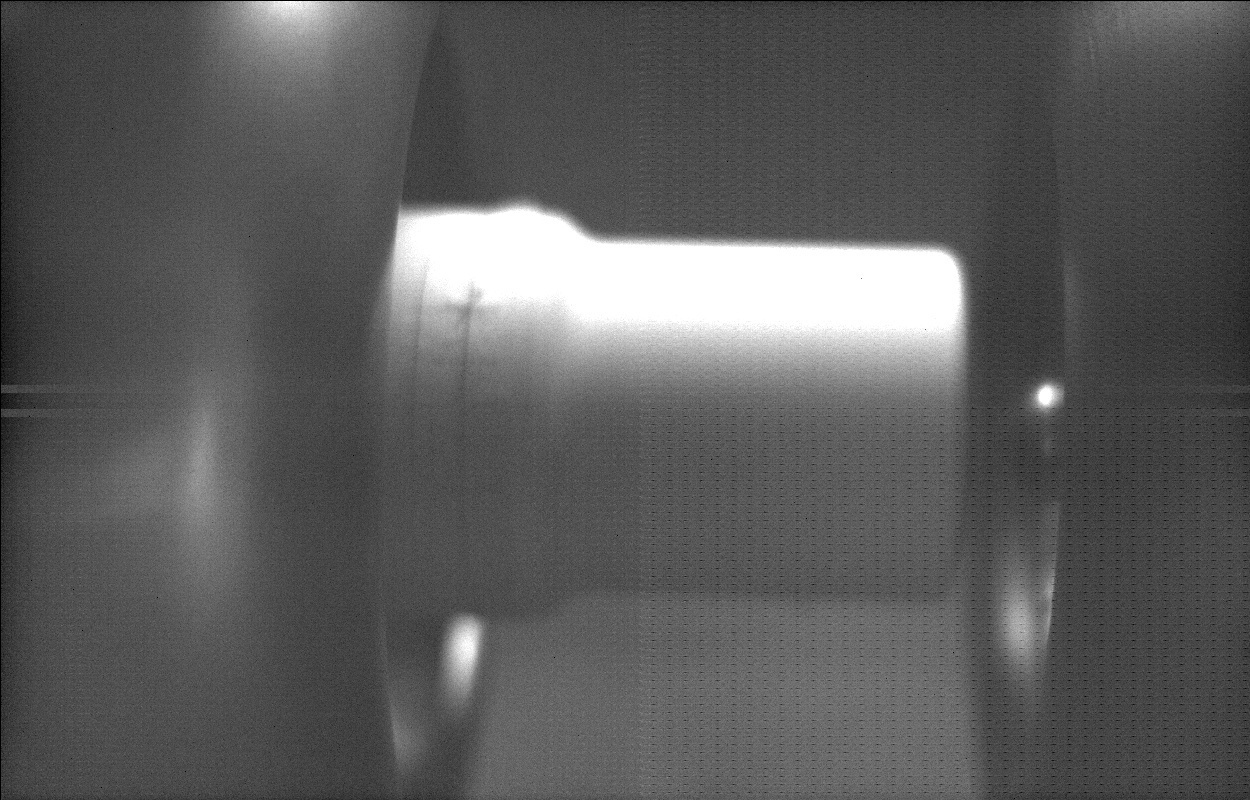

Supplement: S1 File — The relevant data can be obtained from the supporting information. The supporting information is mainly the image data analyzed in this paper. (ZIP) [file pone.0312253.s001.zip › supporting information/Nozzle/OPEN (80).jpg]

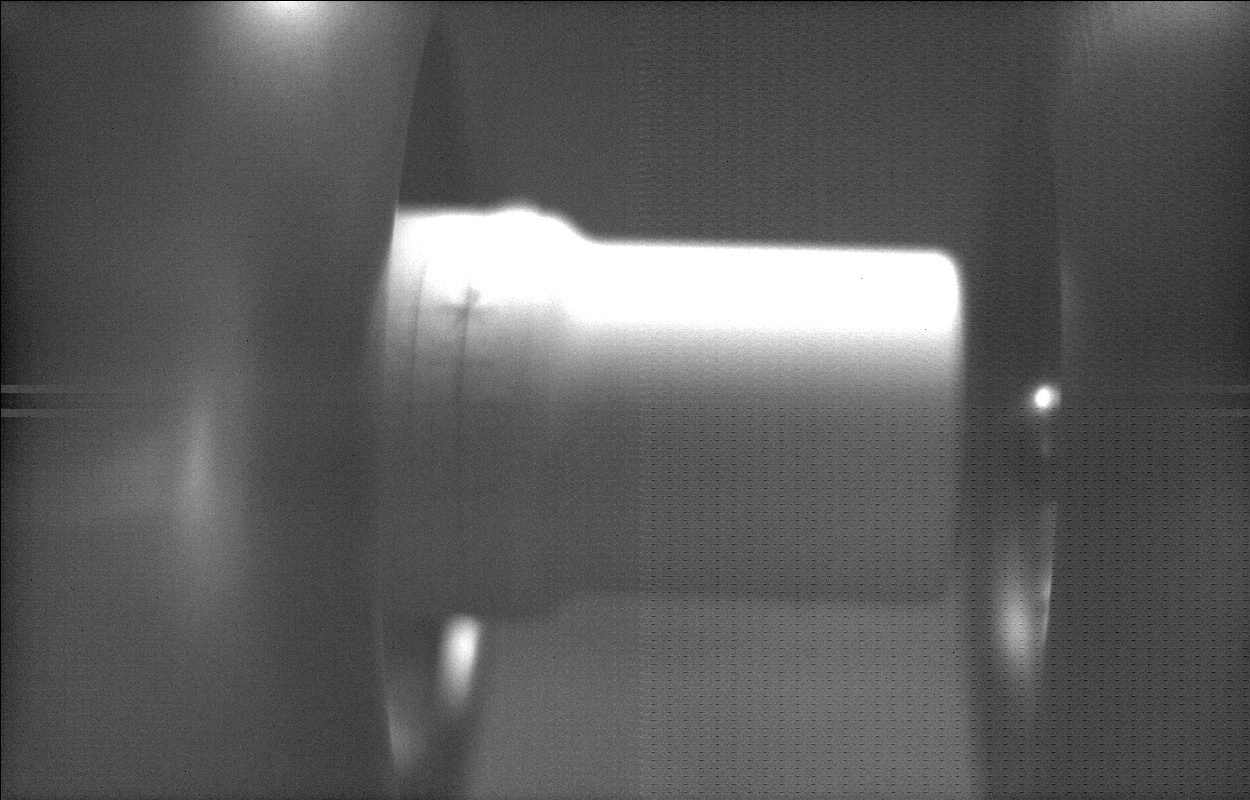

Supplement: S1 File — The relevant data can be obtained from the supporting information. The supporting information is mainly the image data analyzed in this paper. (ZIP) [file pone.0312253.s001.zip › supporting information/Nozzle/OPEN (81).jpg]

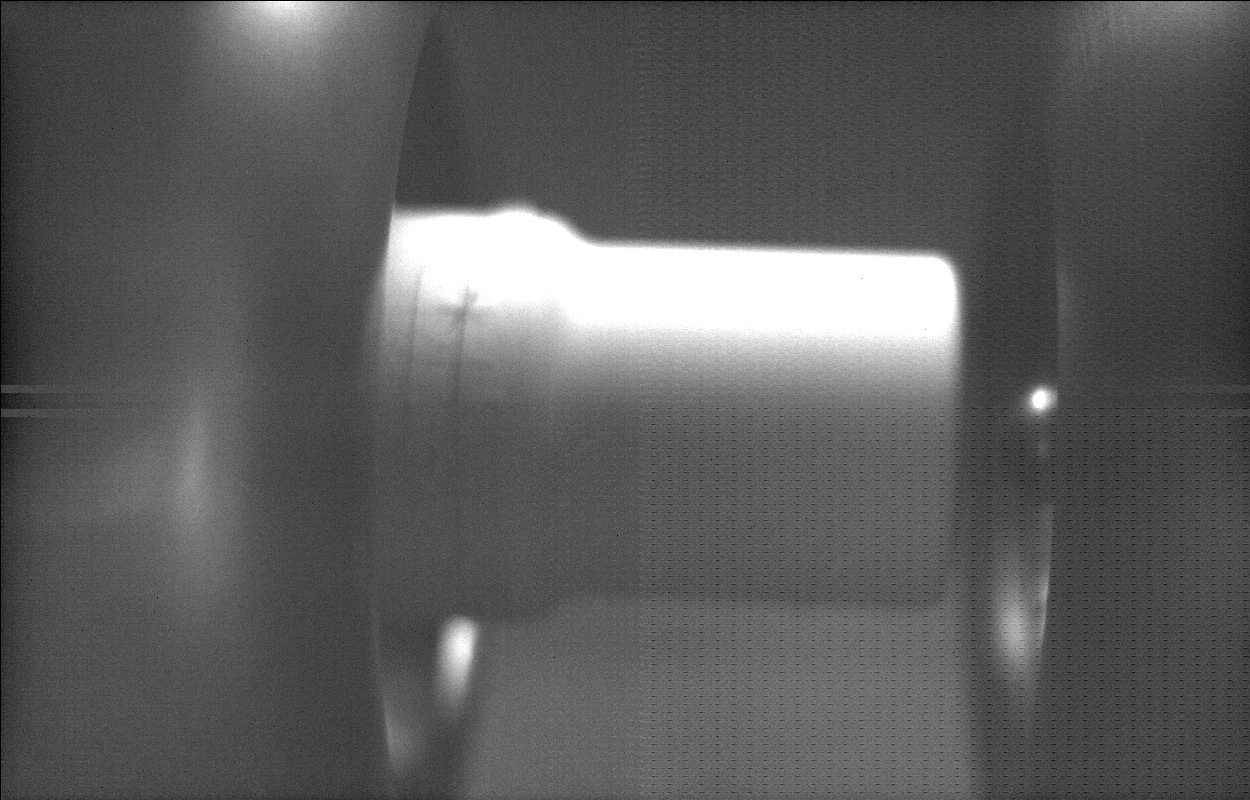

Supplement: S1 File — The relevant data can be obtained from the supporting information. The supporting information is mainly the image data analyzed in this paper. (ZIP) [file pone.0312253.s001.zip › supporting information/Nozzle/OPEN (82).jpg]

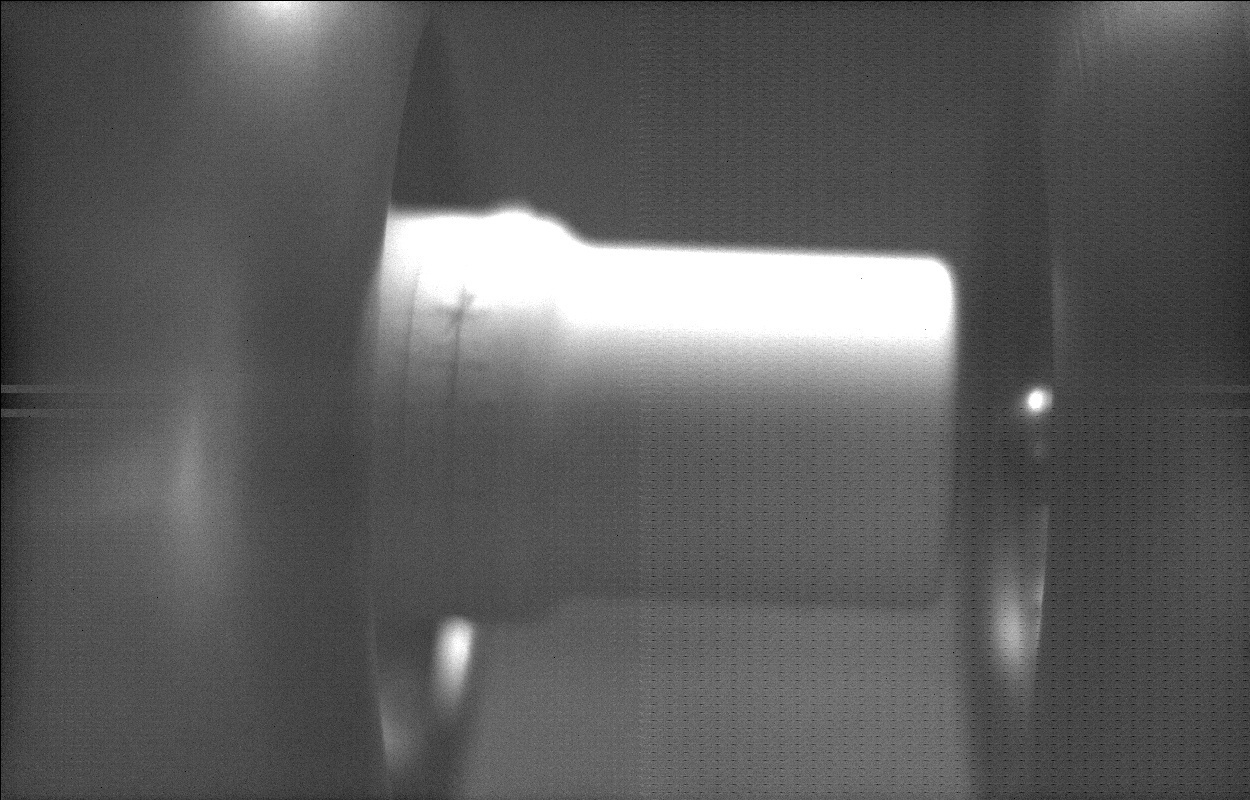

Supplement: S1 File — The relevant data can be obtained from the supporting information. The supporting information is mainly the image data analyzed in this paper. (ZIP) [file pone.0312253.s001.zip › supporting information/Nozzle/OPEN (83).jpg]

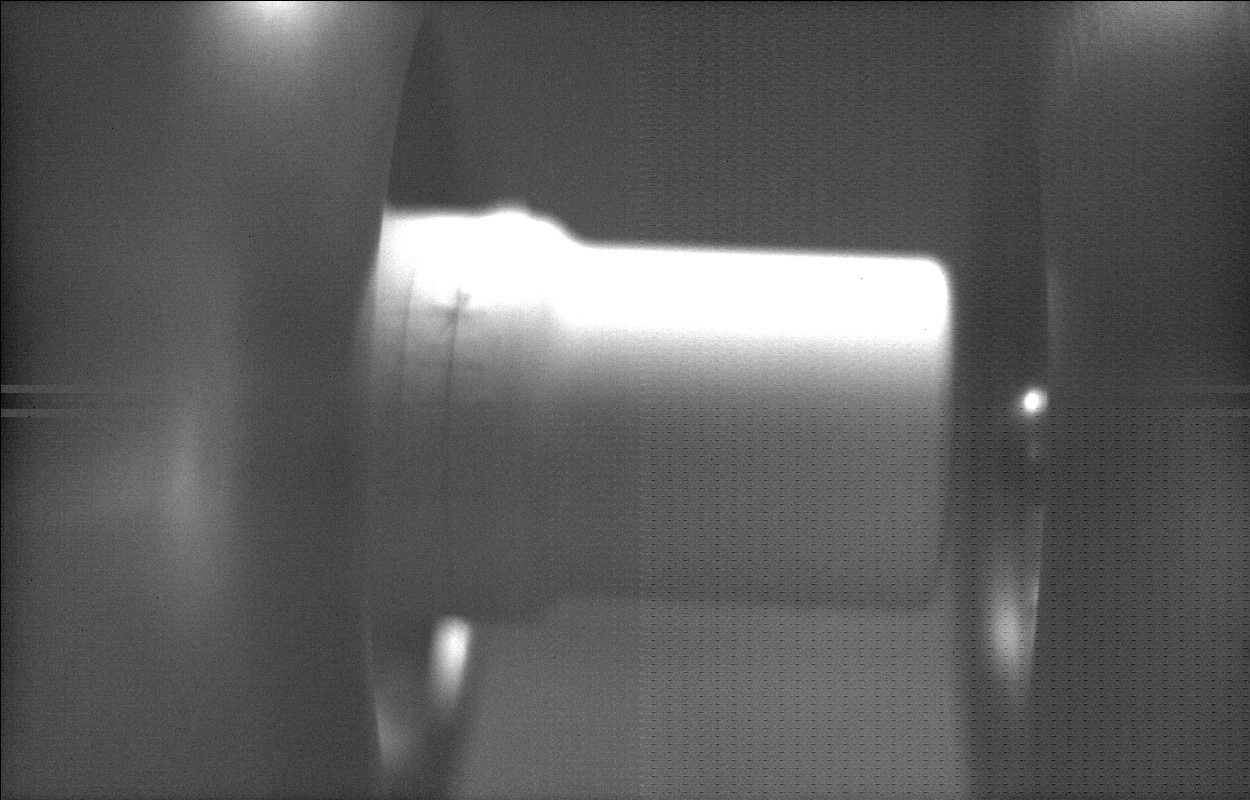

Supplement: S1 File — The relevant data can be obtained from the supporting information. The supporting information is mainly the image data analyzed in this paper. (ZIP) [file pone.0312253.s001.zip › supporting information/Nozzle/OPEN (84).jpg]

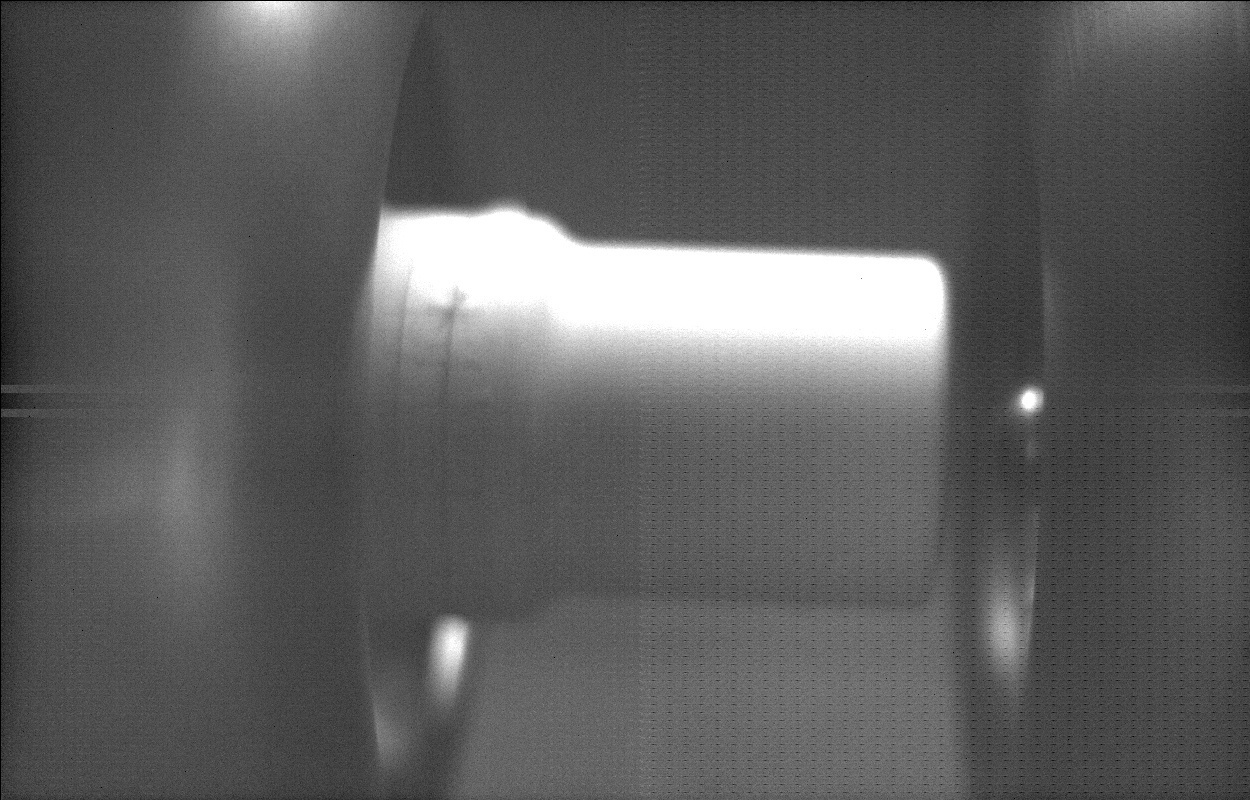

Supplement: S1 File — The relevant data can be obtained from the supporting information. The supporting information is mainly the image data analyzed in this paper. (ZIP) [file pone.0312253.s001.zip › supporting information/Nozzle/OPEN (85).jpg]

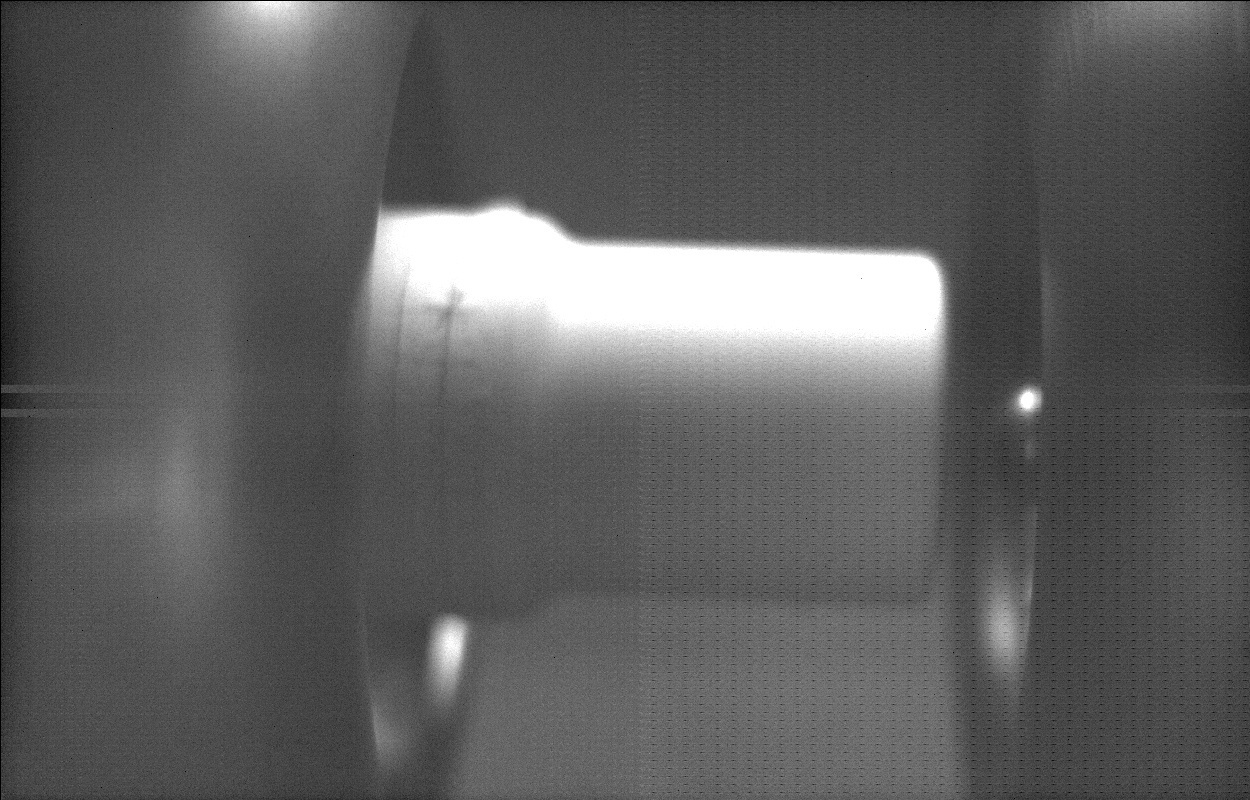

Supplement: S1 File — The relevant data can be obtained from the supporting information. The supporting information is mainly the image data analyzed in this paper. (ZIP) [file pone.0312253.s001.zip › supporting information/Nozzle/OPEN (86).jpg]

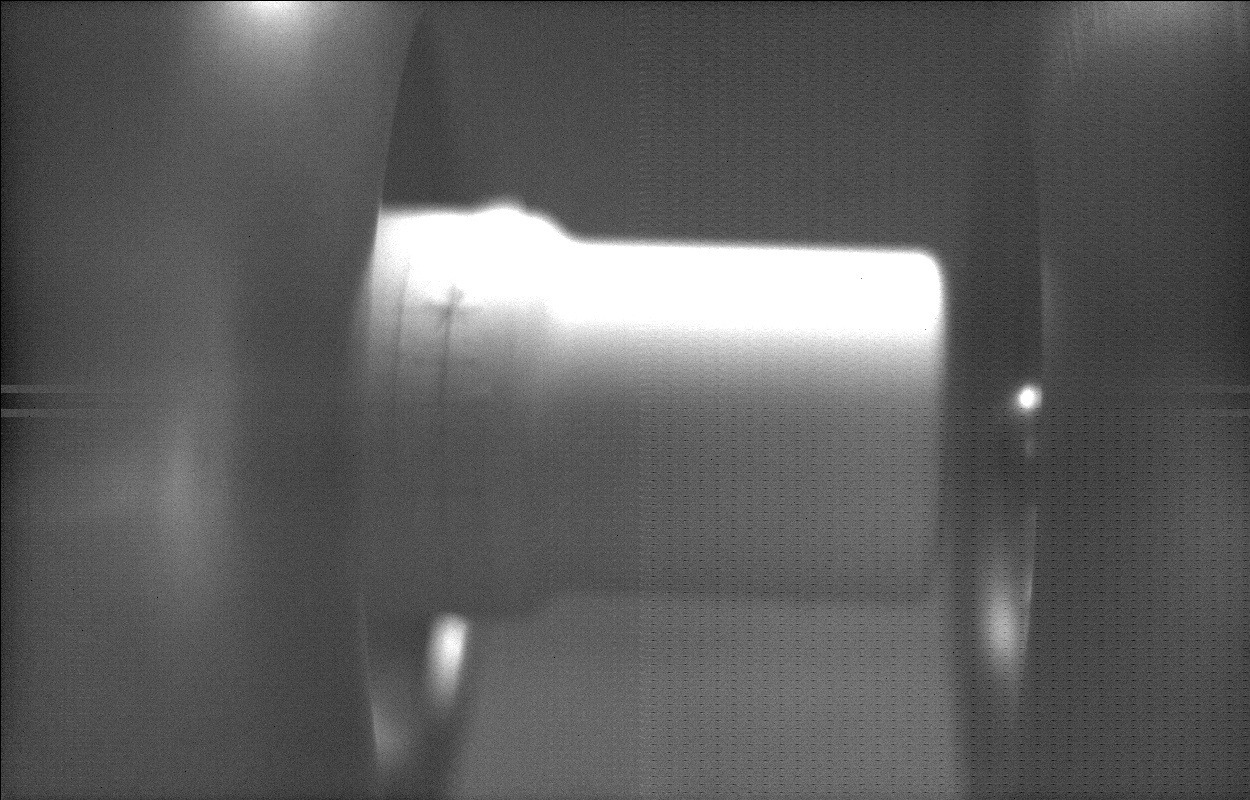

Supplement: S1 File — The relevant data can be obtained from the supporting information. The supporting information is mainly the image data analyzed in this paper. (ZIP) [file pone.0312253.s001.zip › supporting information/Nozzle/OPEN (87).jpg]

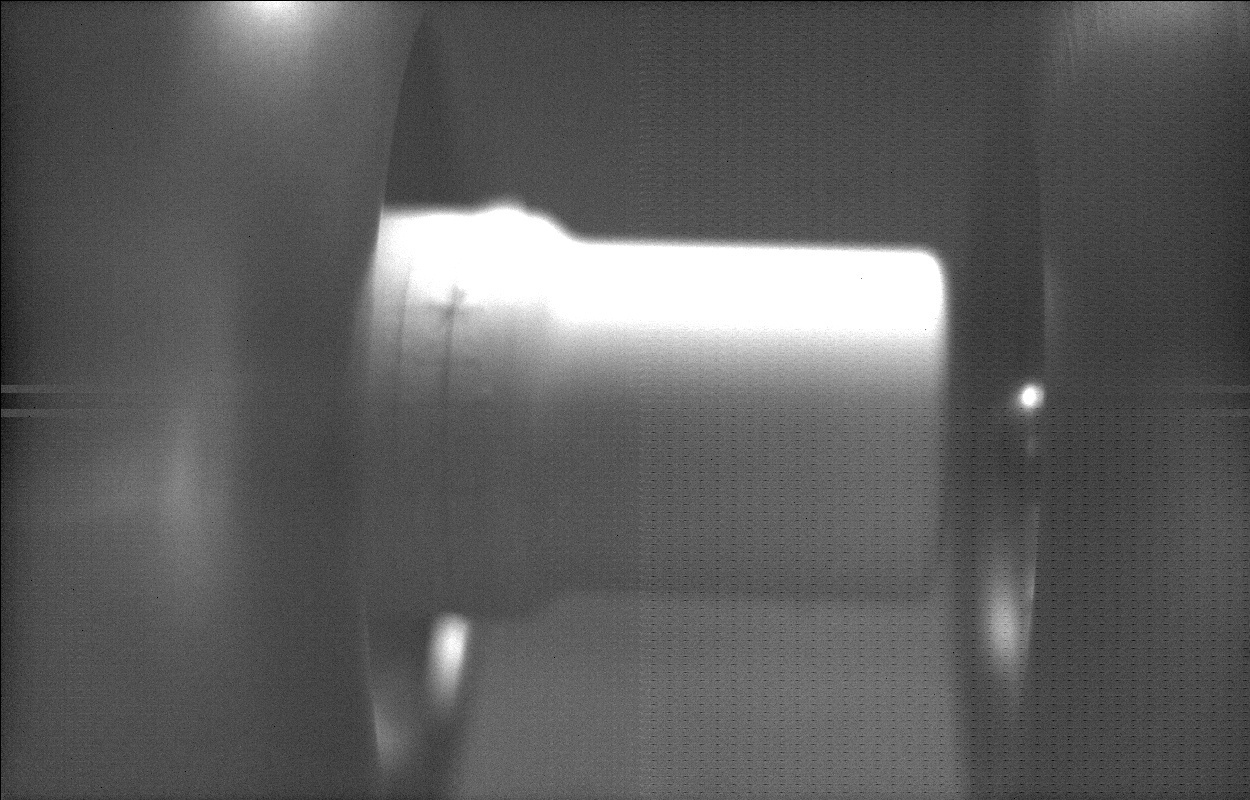

Supplement: S1 File — The relevant data can be obtained from the supporting information. The supporting information is mainly the image data analyzed in this paper. (ZIP) [file pone.0312253.s001.zip › supporting information/Nozzle/OPEN (88).jpg]

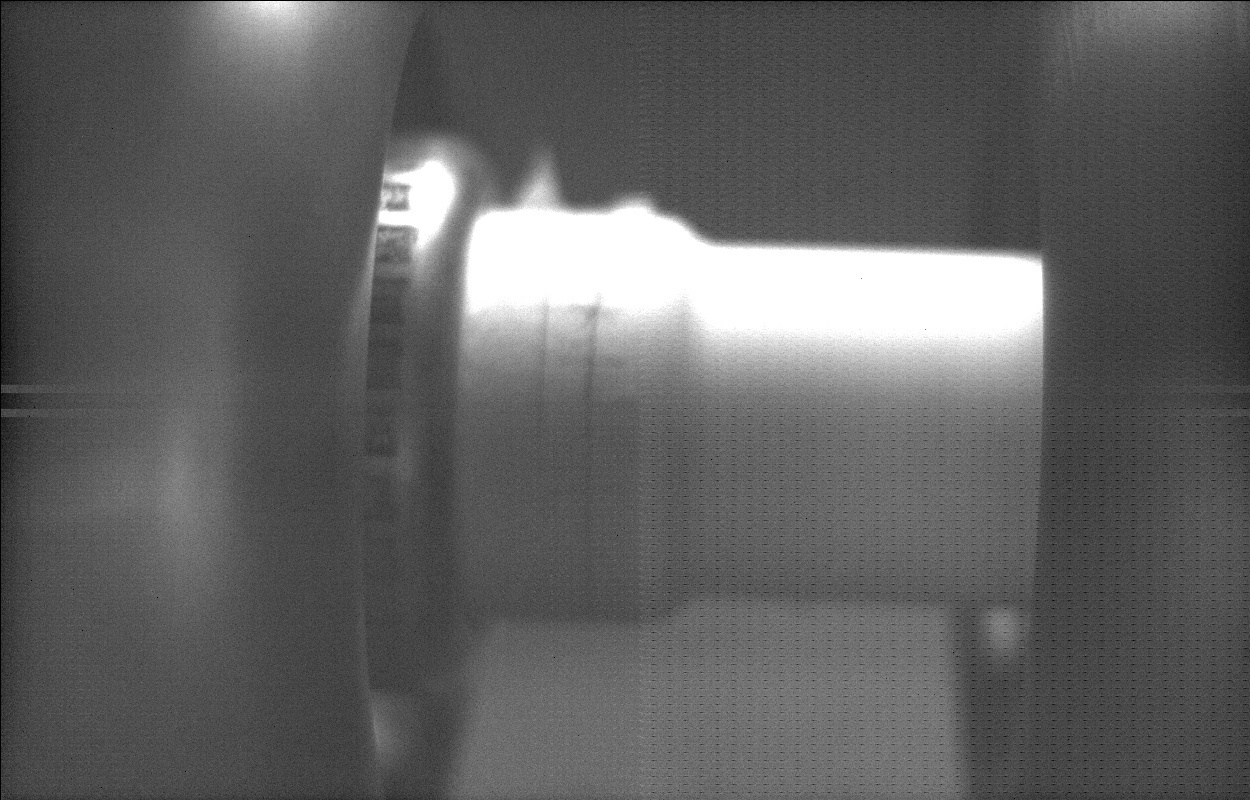

Supplement: S1 File — The relevant data can be obtained from the supporting information. The supporting information is mainly the image data analyzed in this paper. (ZIP) [file pone.0312253.s001.zip › supporting information/Nozzle/OPEN (9).jpg]

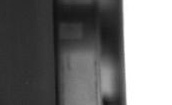

Supplement: S1 File — The relevant data can be obtained from the supporting information. The supporting information is mainly the image data analyzed in this paper. (ZIP) [file pone.0312253.s001.zip › supporting information/repulsion disc/fen1.jpg]

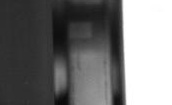

Supplement: S1 File — The relevant data can be obtained from the supporting information. The supporting information is mainly the image data analyzed in this paper. (ZIP) [file pone.0312253.s001.zip › supporting information/repulsion disc/fen10.jpg]

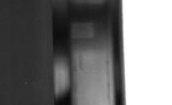

Supplement: S1 File — The relevant data can be obtained from the supporting information. The supporting information is mainly the image data analyzed in this paper. (ZIP) [file pone.0312253.s001.zip › supporting information/repulsion disc/fen11.jpg]

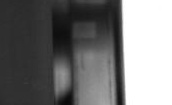

Supplement: S1 File — The relevant data can be obtained from the supporting information. The supporting information is mainly the image data analyzed in this paper. (ZIP) [file pone.0312253.s001.zip › supporting information/repulsion disc/fen12.jpg]

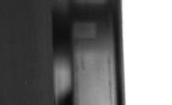

Supplement: S1 File — The relevant data can be obtained from the supporting information. The supporting information is mainly the image data analyzed in this paper. (ZIP) [file pone.0312253.s001.zip › supporting information/repulsion disc/fen13.jpg]

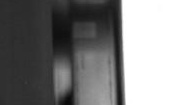

Supplement: S1 File — The relevant data can be obtained from the supporting information. The supporting information is mainly the image data analyzed in this paper. (ZIP) [file pone.0312253.s001.zip › supporting information/repulsion disc/fen14.jpg]

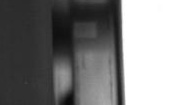

Supplement: S1 File — The relevant data can be obtained from the supporting information. The supporting information is mainly the image data analyzed in this paper. (ZIP) [file pone.0312253.s001.zip › supporting information/repulsion disc/fen15.jpg]

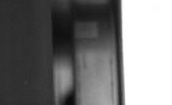

Supplement: S1 File — The relevant data can be obtained from the supporting information. The supporting information is mainly the image data analyzed in this paper. (ZIP) [file pone.0312253.s001.zip › supporting information/repulsion disc/fen16.jpg]

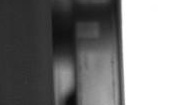

Supplement: S1 File — The relevant data can be obtained from the supporting information. The supporting information is mainly the image data analyzed in this paper. (ZIP) [file pone.0312253.s001.zip › supporting information/repulsion disc/fen17.jpg]

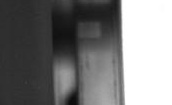

Supplement: S1 File — The relevant data can be obtained from the supporting information. The supporting information is mainly the image data analyzed in this paper. (ZIP) [file pone.0312253.s001.zip › supporting information/repulsion disc/fen18.jpg]

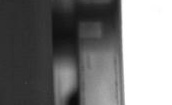

Supplement: S1 File — The relevant data can be obtained from the supporting information. The supporting information is mainly the image data analyzed in this paper. (ZIP) [file pone.0312253.s001.zip › supporting information/repulsion disc/fen19.jpg]

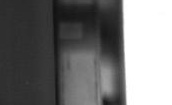

Supplement: S1 File — The relevant data can be obtained from the supporting information. The supporting information is mainly the image data analyzed in this paper. (ZIP) [file pone.0312253.s001.zip › supporting information/repulsion disc/fen2.jpg]
